# Unsupervised Deep Learning of Electronic Health Records to Characterize Heterogeneity Across Alzheimer Disease and Related Dementias: Cross-Sectional Study

**DOI:** 10.2196/65178

**Published:** 2025-03-31

**Authors:** Matthew West, You Cheng, Yingnan He, Yu Leng, Colin Magdamo, Bradley T Hyman, John R Dickson, Alberto Serrano-Pozo, Deborah Blacker, Sudeshna Das

**Affiliations:** 1 Massachusetts General Hospital Cambridge, MA United States

**Keywords:** Alzheimer disease and related dementias, electronic health records, large language models, clustering, unsupervised learning

## Abstract

**Background:**

Alzheimer disease and related dementias (ADRD) exhibit prominent heterogeneity. Identifying clinically meaningful ADRD subtypes is essential for tailoring treatments to specific patient phenotypes.

**Objective:**

We aimed to use unsupervised learning techniques on electronic health records (EHRs) from memory clinic patients to identify ADRD subtypes.

**Methods:**

We used pretrained embeddings of non-ADRD diagnosis codes (*International Classification of Diseases, Ninth Revision*) and large language model (LLM)–derived embeddings of clinical notes from patient EHRs. Hierarchical clustering of these embeddings was used to identify ADRD subtypes. Clusters were characterized regarding their demographic and clinical features.

**Results:**

We analyzed a cohort of 3454 patients with ADRD from a memory clinic at Massachusetts General Hospital, each with a specialist diagnosis. Clustering pretrained embeddings of the non-ADRD diagnosis codes in patient EHRs revealed the following 3 patient subtypes: one with skin conditions, another with psychiatric disorders and an earlier age of onset, and a third with diabetes complications. Similarly, using LLM-derived embeddings of clinical notes, we identified 3 subtypes of patients as follows: one with psychiatric manifestations and higher prevalence of female participants (prevalence ratio: 1.59), another with cardiovascular and motor problems and higher prevalence of male participants (prevalence ratio: 1.75), and a third one with geriatric health disorders. Notably, we observed significant overlap between clusters from both data modalities (*χ*^2^_4_=89.4; *P*<.001).

**Conclusions:**

By integrating *International Classification of Diseases, Ninth Revision* codes and LLM-derived embeddings, our analysis delineated 2 distinct ADRD subtypes with sex-specific comorbid and clinical presentations, offering insights for potential precision medicine approaches.

## Introduction

### Background

Alzheimer disease (AD) is a neurodegenerative condition which affects more than 55 million people globally [1], and it is the seventh leading cause of death in the United States [2]. Despite its substantial public health burden, AD remains poorly understood, with limited treatment options available. AD and related dementias (ADRD) is an umbrella term that refers to multiple dementing illnesses, including AD, frontotemporal dementia (FTD), Lewy body dementia (LBD), and vascular dementia. AD is the most prevalent, accounting for around 60% to 80% of all dementia [2]. While these diseases have distinct clinical and neuropathological criteria, there is substantial overlap in both clinical presentation and autopsy findings at the individual patient level. For example, AD is clinically characterized by an amnestic-predominant dementia and neuropathologically defined by the build-up of amyloid beta (Aβ) plaques and neurofibrillary tangles formed by hyperphosphorylated tau protein [3]; however, these lesions are frequently accompanied by cerebrovascular disease (CVD) [4] or Lewy body pathology [5], which can influence clinical presentation. Likewise, LBD is defined by Lewy bodies but is also associated with plaques and tangles [6], which may accelerate the rate of cognitive decline [7]. This clinical and neuropathological heterogeneity limits our ability to target disease-modifying drugs to each specific neuropathological lesion. The so-called *amyloid hypothesis* has prevailed as the leading explanation of AD disease etiology, where it is held that Aβ toxicity leads to tau hyperphosphorylation, synaptic dysfunction, and neurodegeneration [8]. However, treatments targeting this hypothesis only show limited efficacy, which may stem in part from the clinical and neuropathological comorbidities [9], highlighting the need for a tailored approach to identify potential subtypes of disease and develop more effective targeted treatments.

Previous approaches to AD subtyping have focused on RNA expression, as well as brain imaging and cognitive assessments. Neff et al [10] identified 5 molecular subtypes of AD using RNA-sequencing signatures, characterized by different dysregulated pathways related to tau-mediated neurodegeneration, Aβ neuroinflammation, synaptic signaling, immune activity, mitochondria organization, and myelination. The Subtype and Stage Inference algorithm, applied to magnetic resonance imaging and positron emission tomography imaging data, identified distinct AD trajectories based on the rate and sequence of brain atrophy [11] and tau deposition [12]. Cognitive subtypes have also been identified based on memory, visuospatial and linguistic capabilities, and executive function [13-15]. These studies were limited to research cohorts with specific selection criteria, and it is unclear whether these subtypes can be extended to larger samples.

In contrast, real-world data, such as, electronic health records (EHRs), provide readily accessible large observational datasets and have been used for clustering AD or ADRD subtypes [16]. Unsupervised learning approaches on EHR datasets have revealed latent structure in conditions, such as autism [17,18] and Parkinson disease [19]. For AD subtyping, EHR-based approaches have used the *International Classification of Diseases* (*ICD*) or similar diagnostic codes, showing varying success depending on the methodology and population. Xu et al [20] used hierarchical clustering on EHR data from patients with AD, identifying subtypes related to CVD, mental illness, age of onset, and sensory problems. Alexander et al [21] found the following 5 patient subtypes: mental health, nontypical AD, typical AD, CVD, and men with cancer. They later identified a consistent subtype with early-onset AD, predominantly female participants, with a faster rate of progression using various machine learning methods [22]. Landi et al [23] used unsupervised deep learning to encode EHRs with temporal information, identifying early-onset AD, later-onset AD with mild comorbidities, and typical-onset AD with moderate symptoms. He et al [24] applied spectral clustering to EHRs of patients with AD, discerning 4 subtypes with significant demographic, mortality, and medication use differences. Tang et al [25] analyzed comorbidity patterns in EHRs of patients with AD, revealing sex-dependent variations. In another study, Tang et al [26] used EHRs with knowledge networks to predict AD onset and identify sex-specific genetic markers. These studies collectively highlight the varied methodologies and results in EHR-based research, emphasizing the complexity and potential of these approaches for a deeper understanding of AD.

However, none of these prior studies leveraged the richer representation of EHR data by embedding full sequences of clinical text. Transformers have emerged as state-of-the-art architecture for language modeling and are broadly characterized by the concept of attention [27]. Attention, named for its similarity to cognitive attention, enables the sharing of contextual information among word representations without directly encoding their sequence. The transformer used in this work is a version of the Bidirectional Encoder Representations from Transformers (BERT) architecture [28]. This architecture consists of an encoder which can be fine-tuned on downstream applications and domains. Specifically, we use Clinical BERT [29], which is pretrained on a large corpus of clinical notes from the critical care database Medical Information Mart for Intensive Care (MIMIC) [30].

### Objectives

In this work, we used both pretrained embeddings of *ICD-9* code diagnostic data and transformer-derived embeddings of clinical notes. This dual approach addresses the limitations of previous studies by incorporating structured *ICD* codes, which allow us to study subtypes of patients with similar *ICD* codes (non-ADRD diagnosis in charts), and unstructured clinical notes, which capture detailed clinical history and manifestations provided by specialists. By combining these 2 modalities, we aimed to enhance the clustering of patient ADRD subtypes.

## Methods

### Cohort Selection Process

Patients were selected from the Massachusetts General Hospital (MGH) EHR database. The selection criteria included patients who had at least 2 MGH memory clinic visits (either an in-person office visit or a video telemedicine visit) from August 2015 to June 2022, were aged >50 years at their first visit, and had progress notes of substantial length (≥512 characters). These criteria were chosen due to the richness of the notes for the clustering analysis and the high quality of the ADRD diagnosis from specialists. From the identified patient cohort, 2 datasets were extracted as follows: one containing structured diagnostic *ICD* code data from the patients’ entire medical history and another consisting of unstructured clinical notes authored by memory clinic specialists, limited to the most recent visit. We chose only the most recent visit note because it typically consolidates the patient’s prior history, thereby reducing redundancy and providing a focused, up-to-date clinical snapshot. In addition, the dataset was filtered to exclude patients who did not have ADRD diagnoses (Multimedia Appendix 1), as well as those who lacked non-ADRD *ICD* codes (ie, patients who only had ADRD *ICD* codes were excluded).

### Ethical Considerations

This study was approved by the Mass General Brigham Institutional Review Board (protocol 2015P001915), which granted a waiver of informed consent for secondary analysis of electronic health data. No participant compensation was provided. Electronic health data was queried from Epic and securely stored on servers within the Mass General Brigham firewall. Access was restricted to authorized study personnel, in full compliance with institutional privacy and data security policies.

### Embedding Methodology

#### ICD Codes

Before clustering, it was necessary to derive a patient-level representation that encoded information relevant to phenotype in a single vector. While some prior work has relied on one-hot encoding (where categorical data are converted into binary vectors) of clinical data to represent patient phenotype, we leverage existing pretrained embeddings that capture relevant biomedical semantics in their latent representations of clinical concepts. In particular, we use a set of 300-dimensional embeddings for *ICD-9* codes, derived from prior work by Choi et al [31].

For a count-based encoded representation of *m ICD-9* codes across our cohort, *P* *∈* R^3454^*^×m^*, and an embedding matrix, *E∈*R*^m×^*^300^, our design matrix for clustering, *X* *∈* R^3454^*^×^*^300^, is given by the following matrix multiplication: *X = P · E.*

**Figure 1:**
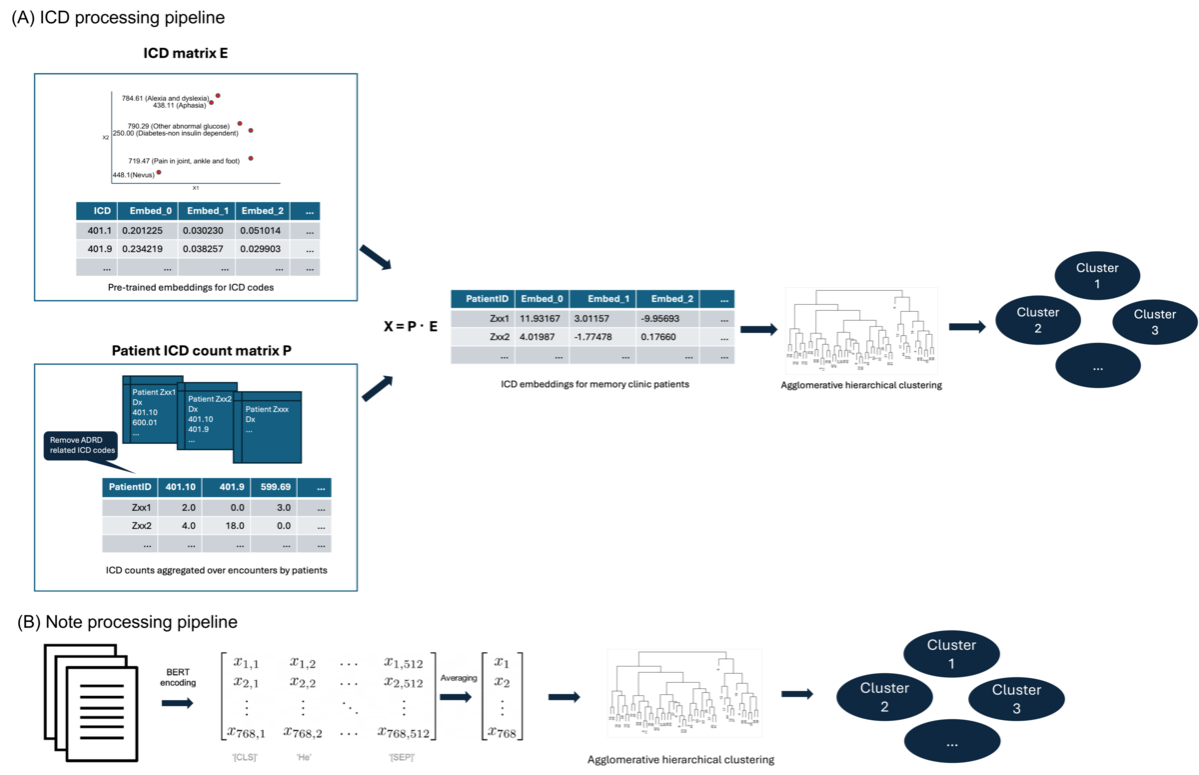
Visualization of the clustering pipeline for (A) International Classification of Diseases (ICD) codes and (B) notes. For each subfigure, the workflow goes from left to right. BERT: Bidirectional Encoder Representations from Transformers.

This matrix multiplication sums the non-ADRD *ICD* embeddings across a patient record, and the resultant embedding is directly affected by the number of times each code appears in a patient’s history. ADRD codes were dropped from the matrix *P* to not confound clustering based on structured ADRD phenotype. A schematic depicting the *ICD* representation pipeline is provided in Figure 1A.

#### Clinical Notes

Clinical notes were encoded using Clinical BERT before clustering. To derive patient-level representations of clinic notes, several preprocessing steps were undertaken. First, unwanted delimiter characters were stripped from patient notes, and notes were chunked into contiguous sections of up to 1024 characters. This resulted in a distribution of token numbers of ∼200 to 300 per note following BERT’s WordPiece encoding of input sequences. After passing through the transformer encoding, we took the final layer representation averaged over the 12 attention heads, such that each note was represented by a matrix of dimension (768, n), corresponding to each of the n input tokens having a 768-dimensional contextual vector representing it. Following this encoding, the representation was averaged over the token dimension to arrive at a single 768-dimensional vector for the whole note. This was explored using both simple averaging (arithmetic mean) over the token dimension, as well as attention-weighted averaging based on row-wise entropy of the final layer attention matrix. Attention-weighted averaging was used in patient representations due to the resultant lower inertia and increased silhouette score on average. A schematic depicting the note representation pipeline is provided in Figure 1B.

### Attention-Weighted Averaging

For a given input sequence of length *n*, the final layer representation in a transformer model has an associated self-attention matrix, *A* *∈* R*^n×^*^n^. For BERT-based models, *n* is *≤*512 due to the constraint on the length of the context window. To provide weights for averaging the embedding, *E* *∈* R^768^*^×n^*, over the token dimension, we compute the row-wise differential entropy of the attention matrix, given in equation (1). Differential entropy is the continuous analog of Shannon entropy, which is usually only defined for discrete random variables [32]. In particular, the method described in Ebrahimi et al [33] is used to approximate the differential entropy, implemented in the Python (Python Software Foundation) library *SciPy version 1.7.3* [34], as the closed-form expression for the attention distribution for a given row *f* (*x*) is not known analytically from the values of attention sampled. The differential entropy for a row *i* is given by



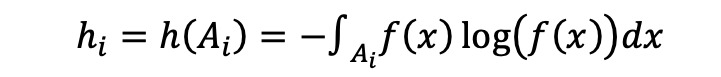
**(1)**


and the corresponding vector *h* *∈* R*^n×^*^1^ corresponds to the entropy across every row. From the row-wise entropy, this vector is softmaxed to obtain the corresponding weights, *w ∈* R*^n×^*^1^, as follows:



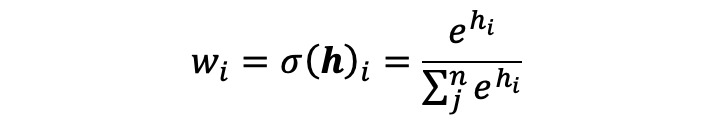
**(2)**


The resultant embedding for a note sequence, *N* *∈* R^768^*^×1^*, is then given by the matrix multiplication as follows:



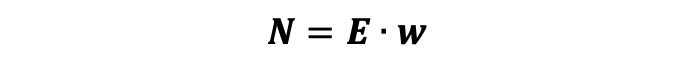
**(3)**


and the final patient-level representation is the simple average over all note fragments for a given patient, for their most recent encounter. A visualization of attention matrices with varying row-wise entropy and thus varying weighting per token is provided in Figures S1 and S2 in Multimedia Appendix 2.

### Hierarchical Clustering

Clustering analysis was performed on *ICD-9* embeddings and clinical text representations to identify ADRD subtypes. We selected hierarchical agglomerative clustering with Ward linkage due to its ability to capture the hierarchical structure of clinical data, as seen in *ICD-9* codes (eg, metabolic disorders branching into type 1 and type 2 diabetes) and clinical notes (eg, cognitive impairment branching into memory loss and aphasia). Cluster quality was evaluated using elbow plots and silhouette scores, with implementation via *Scikit-learn v1.0.1* [35].

### Optimal Transport

To address provider-specific effects in embeddings of clinical notes, we first applied Uniform Manifold Approximation and Projection (UMAP) to reduce the dimensionality of the data and then applied the earth mover distance transport approach using the Python *Optimal Transport* package [36]. *Optimal Transport* provides a mathematical framework to minimize the cost of transforming one distribution into another, which can address the problem of domain adaptation [37]. Domain adaptation involves adjusting data from different sources to make their data distributions more comparable, ensuring that models trained on these data perform well across various settings. In this context, the earth mover distance method was used to align the embeddings from various providers to a standard reference. This alignment ensured that the subsequent clustering analysis was less skewed by provider-related differences, allowing a more accurate interpretation of the underlying phenotypic variation.

### Enrichment Analysis: ICD Clusters

*ICD* clusters were phenotypically characterized by testing for enrichment of *ICD-9* codes within each cluster. For each cluster, a 2×2 contingency table was generated for each *ICD-9* diagnosis code, comparing counts of patients with that code within cluster to counts of patients with that same code in other clusters. A chi-square test for enrichment was performed; the prevalence ratio (PR), calculated as the prevalence of each code in one cluster divided by its prevalence in the rest, was calculated to measure the strength of the association. A Bonferroni correction was applied to the resultant *P* values to correct for multiple comparisons, and *ICD* codes in each cluster were ranked by corrected *P* value to characterize the most significant enrichments. All *P* values in this investigation were two-sided, with a postcorrected α of 0.05 to determine significance. The top-10 significant diagnoses with the highest PR were extracted from each cluster for interpretation. If a cluster lacked significant diagnoses, the top diagnoses with the highest PR among the nonsignificant ones were selected. The enrichment analyses were conducted in Python version 3.8.15.

### Topic Modeling: Note Clusters

We used BERTopic [38], using the Python package *BERTopic* v0.16.0, to identify representative topics and key terms within each note cluster. Embeddings obtained through optimal transport were directly used for clustering and topic assignment, bypassing the need for additional embedding and dimensionality reduction steps. Before conducting cluster-based term frequency–inverse document frequency (TF-IDF) for topic assignment, the clinical text was preprocessed using a vectorizer to remove stop words and exclude common terms that appeared too frequently across most notes. Furthermore, to fine-tune and enhance the word representation of topics, we applied the KeyBERTInspired model, which extracts keywords by leveraging embeddings and cosine similarity to find the words with the closest semantic relationship to the note texts, thereby making them more representative of the topics. Following the extraction of representative terms within each cluster, we used GPT-4 (OpenAI) [39], a state-of-the-art large language model (LLM), to enhance interpretability. GPT-4 summarized the representative words provided by BERTopic into coherent themes with greater clinical significance, such as specific medical conditions, treatments, and medications. The PR, calculated as the prevalence of each word in one cluster divided by its prevalence in the rest, was calculated to measure the strength of the semantic relationship. The BERTopic modeling and analyses were conducted in Python version 3.9.6.

### ADRD Diagnosis Categorization

The categorization of ADRD diagnoses was conducted using an extensive list of diagnostic names based on disease etiology. This list was meticulously reviewed for each unique diagnosis name recorded for the MGH memory clinic patients in the EHR system. The ADRD diagnoses categories included AD; dementia unspecified; FTD; LBD; vascular cognitive impairment (VCI); and others, such as posterior cortical atrophy (PCA), progressive supranuclear palsy, corticobasal degeneration, and primary progressive aphasia. An expert behavioral neurologist (JRD) provided critical input during this process, helping to develop a comprehensive mapping list that correlates specific diagnosis names with their corresponding ADRD categories. The application of this mapping to the data was performed using R version 4.3.2 (R Foundation for Statistical Computing). The full list of diagnosis names corresponding to ADRD diagnosis categories is provided in Multimedia Appendix 1.

### Cluster Characterizations

To assess associations between clusters and sex, as well as ADRD diagnoses, we used the chi-square test. For each cluster, a 2×2 contingency table was generated for each variable, comparing the counts of patients with the characteristic within the cluster to those in other clusters. The PR, defined as the prevalence of a characteristic in one cluster divided by its prevalence in the remaining clusters, was calculated to measure the strength of the association: 1 indicates no difference in prevalence between the 2 groups, >1 indicates higher prevalence in the first group, and <1 indicates lower prevalence in the first group. In addition, to examine variations in the age of onset across clusters, we initially conducted a Kruskal-Wallis Rank Sum Test using the *stats* package from R. *η*^2^ (calculated by subtracting the number of groups from the Kruskal-Wallis H statistic plus one, and then dividing this result by the total number of observations minus the number of groups) based on the H statistic was reported as the effect size: values closer to 0 indicate a smaller effect and values closer to 1 indicate a larger effect. Following significant findings, further post hoc analyses using the Dunn test were performed to delineate differences between groups. The *P* values were adjusted for multiple comparisons using the Benjamini-Hochberg method to control the false discovery rate (FDR). Age-of-onset data were rigorously annotated by human experts reviewing clinical notes; where notes did not specify an exact age of onset, the age at the first clinical visit within the memory clinic was used as an approximation. Finally, we conducted a chi-square test between *ICD* clusters and note clusters to test whether patient cluster assignment was consistent across note and *ICD* representations. If the contingency table was larger than 2×2, Cramér V (calculated as the square root of the chi-square statistic divided by the product of the sample size and the minimum dimension minus one) was reported as the effect size: 0 indicates no association and 1 indicates a strong association. Standardized residuals (standardized differences between the observed count and the expected count) were reported for each cell: values close to 0 indicate the observed count is close to the expected count, positive values indicate the observed count is higher than expected, and negative values indicate the observed count is lower than expected. All statistical analyses were conducted in R version 4.3.2.

## Results

### Study Population

**Figure 2:**
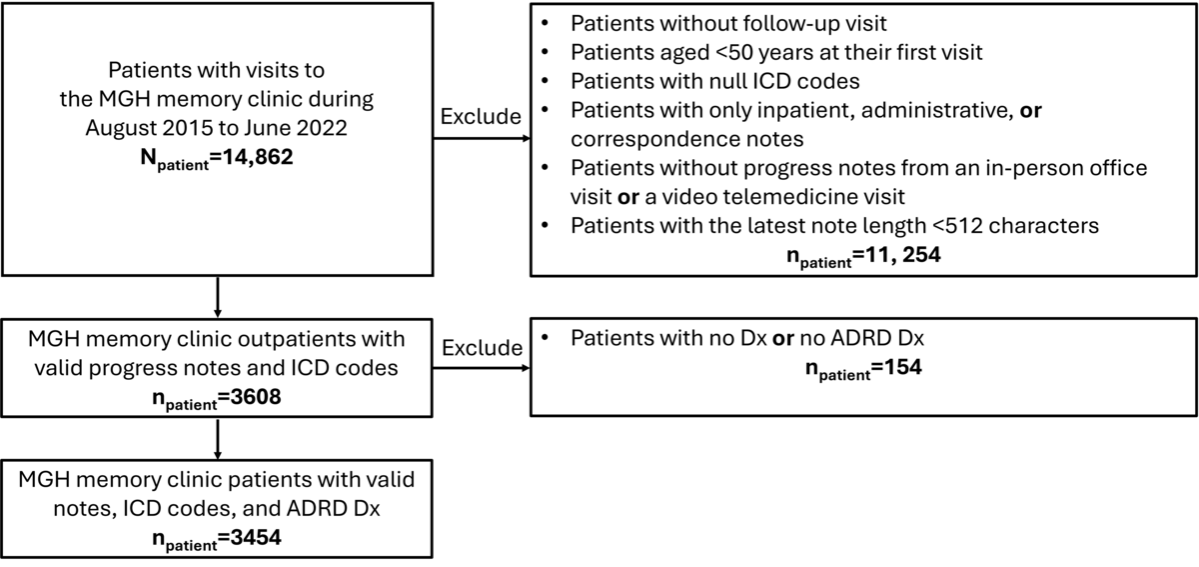
CONSORT (Consolidated Standards of Reporting Trials) diagram illustrating the selection of patients from the Massachusetts General Hospital (MGH) electronic health record (EHR) system. ADRD: Alzheimer disease and related dementias; Dx: diagnosis; ICD: International Classification of Diseases.

Our final study population consisted of 3454 patients from the MGH tertiary care memory clinic with clinical notes and *ICD* codes in the EHR system. The average age of onset for patients was 72.1 (SD 9.5) years, with 1678 (48.58%) being female. The majority were White (n=3059, 88.56%), followed by Asian (n=90, 2.61%), Black or African American (n=77, 2.23%), American Indian or Alaska Native (n=4, 0.12%), and Native Hawaiian or Other Pacific Islander (n=1, 0.03%). In addition, 103 (2.98%) identified as belonging to other races, and race data were not available for 120 (3.47%) patients. Regarding ethnicity, 3020 (87.43%) identified as non-Hispanic, 106 (3.07%) as Hispanic, and ethnicity data were not available for 328 (9.5%) patients. AD was the most prevalent diagnosis, affecting 1317 (38.13%) patients, followed by dementia unspecified, which accounted for 1101 (31.88%) patients. Each encounter recorded only one diagnosis name, and only the most recent encounter was used. The patient selection details are illustrated in Figure 2. The demographic and ADRD diagnosis breakdowns are provided in Table 1 and ADRD categorization details are provided in Multimedia Appendix 1.

**Table 1:** Summary statistics of the final study population.

Characteristics			Total (N=3454)		AD^a^ (n=1317)		Dementia unspecified (n=1101)		FTD^b^ (n=190)		LBD^c^ (n=261)		VCI^d^ (n=96)		Other^e^ (n=489)
Age of onset^f^ (y), mean (SD)			72.1 (9.5)		74.6 (7.8)		73.3 (9.7)		63.8 (8.2)		71.4 (7.7)		76 (7.7)		65.1 (9.4)
**Sex, n (%)**															
	Female	1678 (48.58)		717 (54.44)		538 (48.86)		78 (41.1)		64 (24.5)		41 (43)		240 (49.1)	
	Male	1776 (51.42)		600 (45.56)		563 (51.14)		112 (58.9)		197 (75.5)		55 (57)		249 (50.9)	
**Race, n (%)**															
	White	3059 (88.56)		1149 (87.24)		988 (89.74)		168 (88.4)		227 (87.0)		80 (83)		447 (91.4)	
	Black or African American	77 (2.23)		27 (2.05)		22 (2.00)		6 (3.2)		4 (1.5)		9 (9)		9 (1.8)	
	Asian	90 (2.61)		34 (2.58)		31 (2.82)		6 (3.2)		14 (5.4)		1 (1)		4 (0.8)	
	American Indian or Alaska Native	4 (0.12)		2 (0.15)		1 (0.09)		0 (0.0)		0 (0.0)		0 (0)		1 (0.2)	
	Native Hawaiian or Other Pacific Islander	1 (0.03)		1 (0.08)		0 (0)		0 (0)		0 (0)		0 (0)		0 (0)	
	Other	103 (2.98)		52 (3.95)		32 (2.91)		2 (1.1)		2 (0.8)		3 (3)		12 (2.5)	
	Unavailable	120 (3.47)		52 (3.95)		27 (2.45)		8 (4.2)		14 (5.4)		3 (3)		16 (3.3)	
**Ethnicity, n (%)**															
	Not Hispanic or Latino	3020 (87.43)		1133 (86.03)		987 (89.65)		161 (84.7)		228 (87.4)		83 (87)		428 (87.5)	
	Hispanic or Latino	106 (3.07)		53 (4.02)		37 (3.36)		2 (1.1)		3 (1.1)		3 (3)		8 (1.6)	
	Unavailable	328 (9.50)		131 (9.95)		77 (6.99)		27 (14.2)		30 (11.5)		10 (10)		53 (10.8)	

^a^AD: Alzheimer disease.

^b^FTD: frontotemporal dementia.

^c^LBD: Lewy body dementia.

^d^VCI: vascular cognitive impairment.

^e^Includes posterior cortical atrophy, progressive supranuclear palsy, corticobasal degeneration, and primary progressive aphasia.

^f^Age of onset was manually annotated by experts viewing clinical notes; for notes without age of onset, we approximated with the age of first encounter.

### ICD Clustering

**Figure 3:**
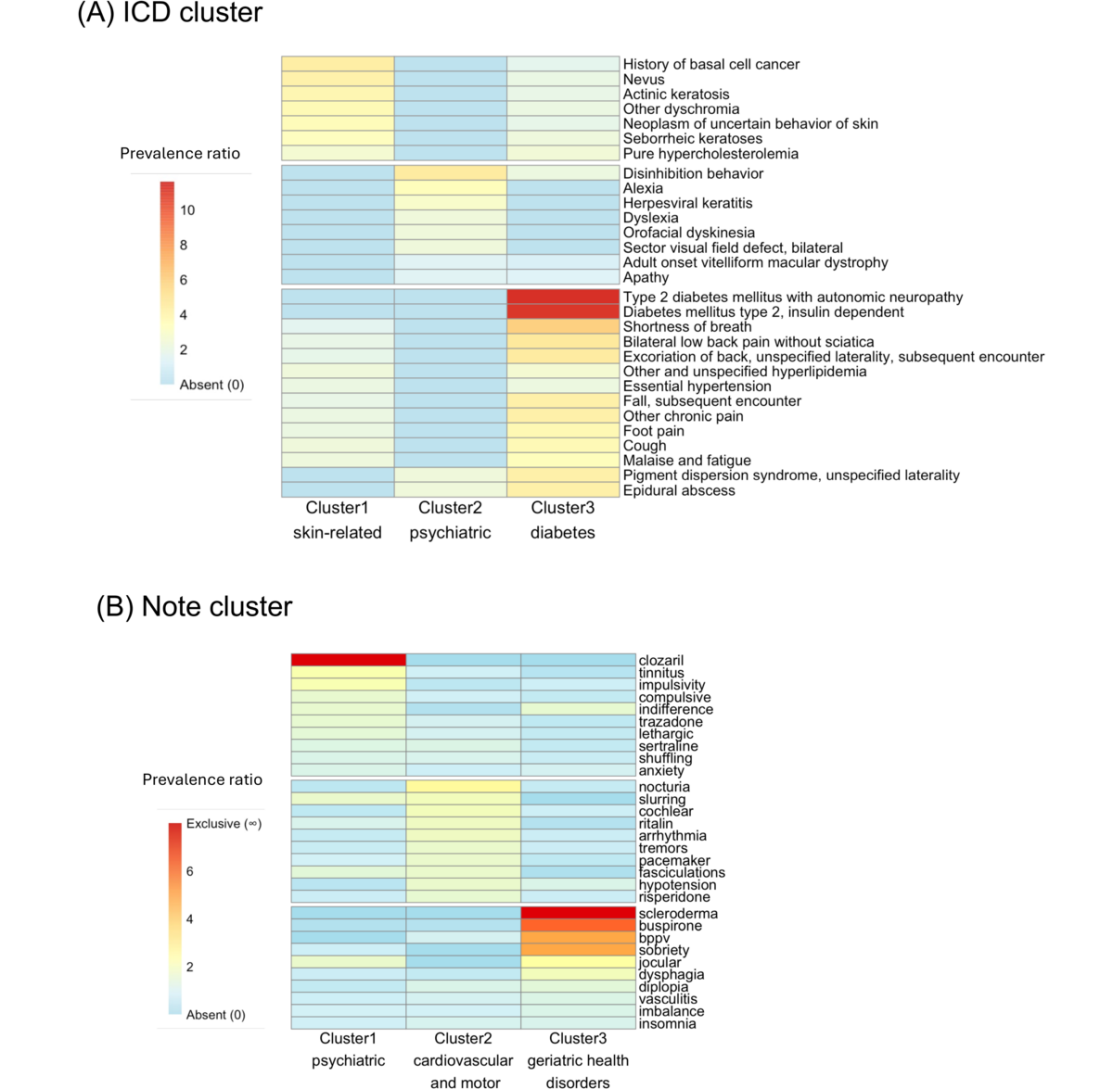
Heatmap of enrichment in International Classification of Diseases (ICD) clusters and topics in note clusters. (A) This heatmap displays the enrichment of ICD-9 codes across ICD embedding clusters. Cluster 1 is primarily dominated by skin-related and certain cardiovascular conditions. Cluster 2 is marked by its exclusive and high prevalence ratios (PR) in psychiatric and behavioral conditions. Cluster 3 shows a diverse set of conditions with a significant prevalence of respiratory, pain-related, and complicated diabetic mellitus. (B) This heatmap displays representative words for each note cluster identified through topic modeling. Cluster 1 is primarily dominated by psychiatric manifestations and medications. Cluster 2 highlights cardiovascular, motor, and sensory issues. Cluster 3 covers a variety of symptoms and conditions, including autoimmune issues, behavioral and movement disorders, sleep disturbances, etc. In both (A) and (B), the color intensity of each code or word-cluster pairing reflects the PR (prevalence of code or word in observed group divided by prevalence in other groups) associated with that code or word. Words colored as exclusive were only present in one cluster.

**Figure 4:**
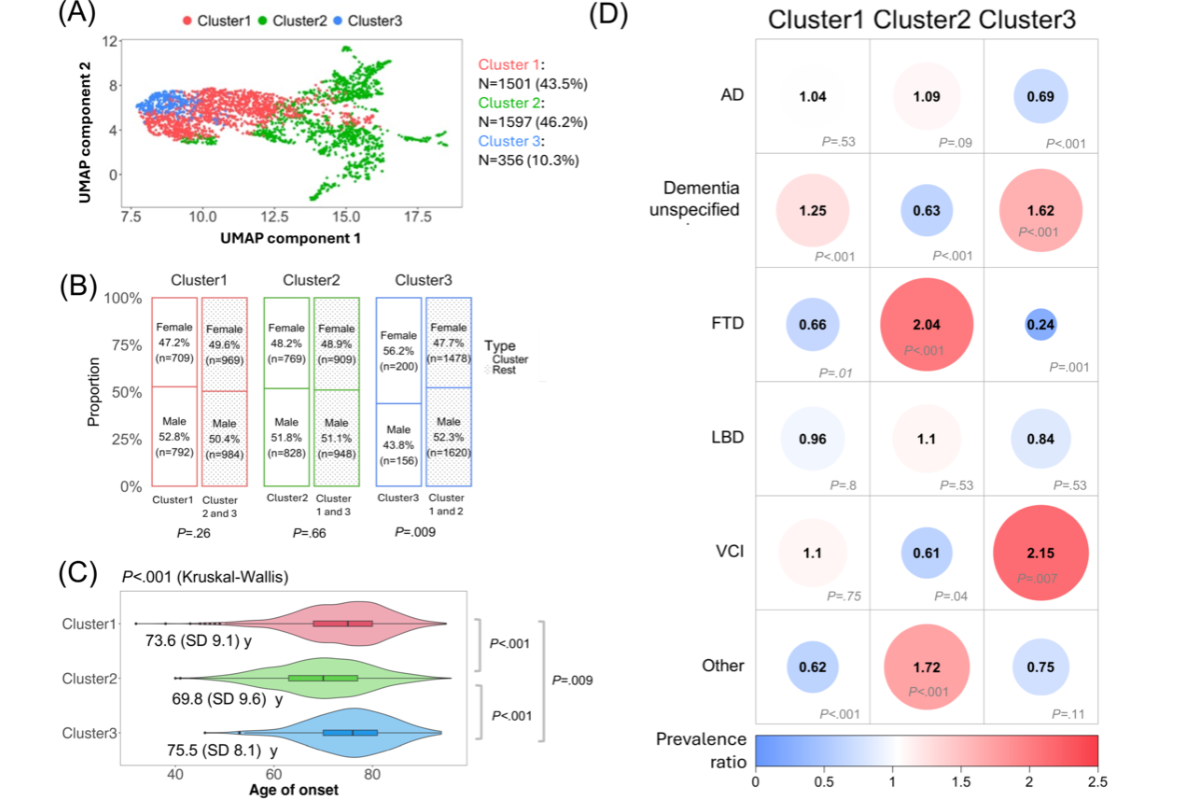
Clustering of International Classification of Diseases (ICD) embeddings and their demographic and diagnostic associations. (A) Uniform Manifold Approximation and Projection (UMAP) visualization of ICD embeddings were characterized by 3 clusters: cluster 1 includes 1501 (43.5%) patients, cluster 2 comprises 1597 (46.2%) patients, and cluster 3 contains 356 (10.3%) patients. (B) Bar plot showing prevalence for sex by cluster, significance based on a chi-square test. Notably, cluster 3 has a significantly higher proportion of female participants compared to male participants (P_FDR_=.009). (C) Violin plot illustrating the distribution of age of onset across clusters. Each violin plot shows the kernel density estimate of the data, with the center line representing the median age of onset. Box plot elements are overlaid, where the box limits indicate the upper and lower quartiles, and the whiskers extend to 1.5 times the IQR. Individual points are hidden for clarity. Significant differences are observed, with cluster 2 showing the earliest average age of onset at 69.8 (SD 9.6) years, and cluster 3 the latest at 75.5 (SD 8.1) years (*P*<.001). (D) Heatmap showing prevalence ratio for Alzheimer disease and related dementias (ADRD) diagnoses across clusters, significance derived from a chi-squared test. Clinical diagnoses include Alzheimer disease (AD); dementia unspecified; frontotemporal dementia (FTD); Lewy body dementia (LBD); vascular cognitive impairment (VCI); and others such as posterior cortical atrophy (PCA), progressive supranuclear palsy (PSP), corticobasal degeneration (CBD), and primary progressive aphasia (PPA). Significant distribution variations are evident across clusters. The circle size, color, and number indicate the magnitude of the prevalence ratio. Blue indicates underrepresentation while red indicates overrepresentation. *P* values were corrected with false discovery rate adjustments.

To investigate the clinical heterogeneity within ADRD, we clustered the embeddings of non-ADRD *ICD* codes assigned to this tertiary care sample from the MGH ADRD cohort. We hypothesized that this approach would reveal distinct clinical subtypes based on clinical comorbidities, which were associated with demographics (ie, age of onset and sex). The hierarchical agglomerative clustering method revealed 3 distinct clusters in the embeddings of non-ADRD *ICD* codes, as determined by the silhouette score. Figure 3A depicts a heatmap of enriched *ICD* codes across each cluster, and a 2D UMAP projection of these *ICD* code embeddings, colored by cluster, is displayed in Figure 4A. The distribution of patients across the clusters was as follows: cluster 1 included 1501 (43.46%) patients, cluster 2 included 1597 (46.24%) patients, and cluster 3 included 356 (10.31%) patients. Detailed summary statistics for these clusters are presented in Table 2.

We examined differences in non-ADRD *ICD* code frequency across patient *ICD* embedding clusters (Figure 3A). In cluster 1, diagnoses, such as seborrheic keratoses (*χ*^2^_1_=243.15; *P*_FDR_<.001; PR=3.32), actinic keratosis (*χ*^2^_1_=236.53; *P*_FDR_<.001; PR=3.91), pure hypercholesterolemia (*χ*^2^_1_=199.13; *P*_FDR_<.001; PR=2.71), history of basal cell cancer (*χ*^2^_1_=196.22; *P*_FDR_<.001; PR=4.45), and nevus (*χ*^2^_1_=184.53; *P*_FDR_<.001; PR=4.23), show notably high PRs. These diagnoses largely fall into skin-related disorders (such as various types of skin cancer and keratoses). Cluster 2 appears to be unique, with top PRs noted only for clinical signs, such as disinhibition behavior (*χ*^2^_1_=1.14; *P*_FDR_=.99; PR=4.65), alexia (*χ*^2^_1_=0.426; *P*_FDR_=.99; PR=3.49), and orofacial dyskinesia (*χ*^2^_1_=0.017; *P*_FDR_=.99; PR=2.33), suggesting behavioral and psychiatric manifestations which are consistent with the FTD enrichment and earlier onset noted above. Notably, although these diagnoses did not reach significance in cluster 2, they were absent in the other clusters. Moreover, many diagnoses in cluster 2 were marked with a lack of PR, indicating a lower relevance of these diagnoses compared to clusters 1 or 3. Cluster 3 exhibited significant increases in diagnoses from a variety of categories, including respiratory issues (eg, cough: *χ*^2^_1_=339.47; *P*_FDR_<.001; PR=3.77 and shortness of breath: *χ*^2^_1_=306.60; *P*_FDR_<.001; PR=6.03), chronic pain (*χ*^2^_1_=460.55; *P*_FDR_<.001; PR=4.80), musculoskeletal problems (eg, bilateral low back pain without sciatica: *χ*^2^_1_=456.91; *P*_FDR_<.001; PR=4.80 and foot pain: *χ*^2^_1_=372.91; *P*_FDR_<.001; PR=3.86), and complications of diabetes mellitus. Notably, diabetes mellitus (type 2 with autonomic neuropathy: *χ*^2^_1_=322.98; *P*_FDR_<.001; PR=11.60 and insulin-dependent diabetes: *χ*^2^_1_=308.96; *P*_FDR_<.001; PR=11.30) had exceptionally high PRs, suggesting a very strong association with these severe diabetes conditions in cluster 3.

**Table 2:** Summary statistics of International Classification of Diseases clusters.

Characteristics			Total (N=3454)	Cluster 1 (n=1501)	Cluster 2 (n=1597)	Cluster 3 (n=356)
Age of onset^a^ (y), mean (SD)			72.1 (9.5)	73.6 (9.1)	69.8 (9.6)	75.5 (8.1)
**Sex, n (%)**						
	Female		1678 (48.58)	709 (47.24)	769 (48.15)	200 (56.2)
	Male		1776 (51.42)	792 (52.76)	828 (51.85)	156 (43.8)
**Race, n (%)**						
	White		3059 (88.56)	1335 (88.94)	1421 (88.98)	303 (85.1)
	Black or African American		77 (2.23)	37 (2.47)	25 (1.57)	15 (4.2)
	Asian		90 (2.61)	40 (2.66)	40 (2.5)	10 (2.8)
	American Indian or Alaska Native		4 (0.12)	0 (0)	2 (0.13)	2 (0.6)
	Native Hawaiian or Other Pacific Islander		1 (0.03)	0 (0)	1 (0.06)	0 (0)
	Other		103 (2.98)	51 (3.4)	34 (2.13)	18 (5.1)
	Unavailable		120 (3.47)	38 (2.53)	74 (4.63)	8 (2.2)
**Ethnicity, n (%)**						
	Not Hispanic or Latino		3020 (87.43)	1358 (90.47)	1326 (83.03)	336 (94.4)
	Hispanic or Latino		106 (3.07)	45 (3)	42 (2.63)	19 (5.3)
	Unavailable		328 (9.5)	98 (6.53)	229 (14.34)	1 (0.3)
**ADRD Dx^b^, n (%)**						
	AD^c^		1317 (38.13)	584 (38.91)	636 (39.82)	97 (27.2)
	Dementia unspecified		1101 (31.88)	540 (35.98)	388 (24.3)	173 (48.6)
	FTD^d^		190 (5.5)	64 (4.26)	121 (7.58)	5 (1.4)
	LBD^e^		261 (7.56)	111 (7.4)	127 (7.95)	23 (6.5)
		VCI^f^	96 (2.78)	44 (2.93)	33 (2.07)	19 (5.3)
	Other^g^		489 (14.16)	158 (10.53)	292 (18.28)	39 (11)

^a^Age of onset was manually annotated by experts viewing clinical notes; for notes without age of onset, we approximated with the age of first encounter.

^b^Dx: diagnosis.

^c^AD: Alzheimer disease.

^d^FTD: frontotemporal dementia.

^e^LBD: Lewy body dementia.

^f^VCI: vascular cognitive impairment.

^g^Includes posterior cortical atrophy, progressive supranuclear palsy, corticobasal degeneration, and primary progressive aphasia.

Furthermore, statistical analyses revealed significant associations between ICD cluster membership, demographic variables, and diagnostic categories. Cluster 3 had an overrepresentation of female participants relative to clusters 1 and 2 (*χ*^2^_1_=8.8; *P*_FDR_=.009; PR=1.178); however, clusters 1 and 2 showed no significant differences in sex distribution (cluster 1: *χ*^2^_1_=1.8; *P*_FDR_=.26 and cluster 2: *χ*^2^_1_=0.2; *P*_FDR_=.66; Figure 4B). In addition, the age of onset varied significantly among the ICD clusters (Kruskal-Wallis *χ*^2^_2_=182.6; *P*_FDR_<.001, *η*^2^=0.052). Cluster 2, with a mean age of onset of 69.8 (SD 9.6) years, had a significantly earlier age of onset compared with clusters 1 (*Z*=10.13; *P*_FDR_<.001) and 3 (*Z*=−8.83; *P*_FDR_<.001). Moreover, cluster 1 (mean 73.6, SD 9.1 years) had an earlier onset than cluster 3 (mean 75.5, SD 8.1 years; *Z*=−2.61; *P*_FDR_=.009; Figure 4C). Cluster 1 was significantly enriched by dementia unspecified (*χ*^2^_1_=20.2; 𝑃_FDR_<.001; PR*=*1.252); cluster 2 was significantly enriched by FTD (*χ*^2^_1_=23.9; 𝑃_FDR_<.001; PR*=*2.039) and other rare ADRDs (*χ*^2^_1_=41; 𝑃_FDR_ <.001; PR*=*1.724); and cluster 3 was significantly enriched by VCI (*χ*^2^_1_=8.6; 𝑃_FDR_=.007; PR*=*2.147) and dementia unspecified (*χ*^2^_1_=50.2; 𝑃_FDR_<.001; PR*=*1.622). In contrast, no cluster was significantly enriched by AD, though AD was significantly underrepresented in cluster 3 (cluster 1: *χ*^2^_1_=0.6; 𝑃_FDR_=.53; cluster 2: *χ*^2^_1_=3.5; 𝑃_FDR_=.09; and cluster 3: *χ*^2^_1_=19.4; 𝑃_FDR_<.001; Figure 4D) or LBD (cluster 1: *χ*^2^_1_=0.1; 𝑃_FDR_=.80; cluster 2: *χ*^2^_1_=0.5; 𝑃_FDR_=.53; and cluster 3: *χ*^2^_1_=0.5; 𝑃_FDR_=.53; Figure 4D). Additional visualizations of the UMAP projections colored by sex, age of onset, and ADRD diagnoses are available in Figures S3A, S3B, and S3C, respectively, in Multimedia Appendix 2.

### Note Clustering

**Figure 5:**
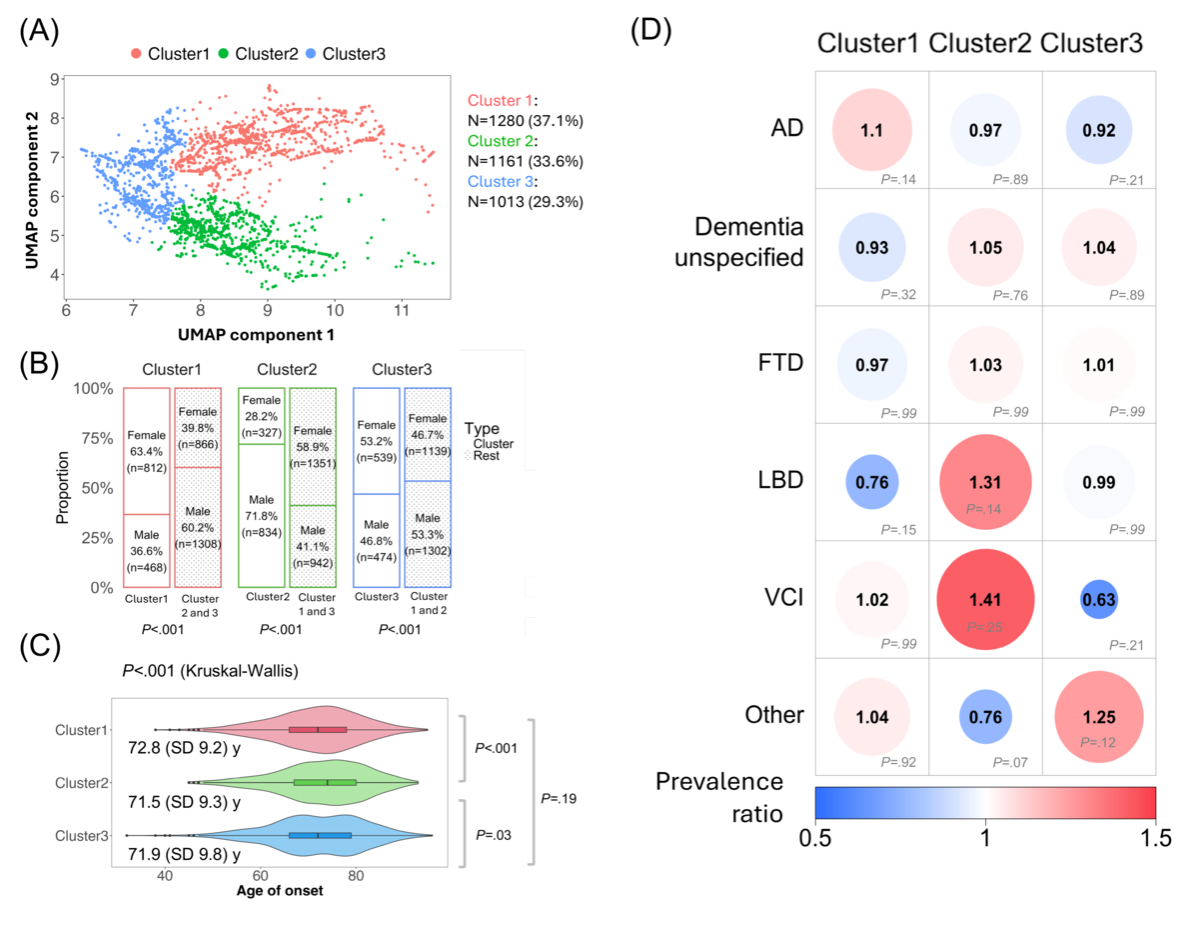
Clustering of note embeddings and their demographic and Alzheimer disease and related dementias (ADRD) diagnosis associations. (A) Uniform Manifold Approximation and Projection (UMAP) visualization of note embeddings characterized by 3 clusters: cluster 1 includes 1280 (37.1%) patients, cluster 2 includes 1161 (33.6%) patients, and cluster 3 includes 1013 (29.3%) patients. (B) Bar plot showing prevalence for sex by cluster, with significance based on chi-square tests. Notably, cluster 1 and cluster 3 were both enriched by female participants (*P*<.001) while cluster 2 was enriched by male participants (*P*<.001). (C) Violin plot illustrating the distribution of age of onset across clusters. Each violin plot shows the kernel density estimate of the data, with the center line representing the median age of onset. Box plot elements are overlaid, where the box limits indicate the upper and lower quartiles, and the whiskers extend to 1.5 times the IQR. Individual points are hidden for clarity. Significant differences are observed, with cluster 2 showing the latest average age of onset at 72.8 (SD 9.2) years, and cluster 1 the earliest at 71.5 (SD 9.3) years (*P*<.001). (D) Heatmap showing prevalence ratio for ADRD diagnoses across clusters, with significance derived from a chi-square test. Diagnoses include Alzheimer disease (AD); dementia unspecified; frontotemporal dementia (FTD); Lewy body dementia (LBD); vascular cognitive impairment (VCI); and others such as posterior cortical atrophy (PCA), progressive supranuclear palsy (PSP), corticobasal degeneration (CBD), and primary progressive aphasia (PPA). No significant distribution variations are observed across clusters (*P*>.05). The circle size, color, and number indicate the magnitude of the prevalence ratio. Blue indicates underrepresentation while red indicates overrepresentation. *P* values were corrected with false discovery rate (FDR) adjustments.

Initially, a provider effect was detected in the UMAP projection of note embeddings from the latest clinical notes of 3454 patients (Figure S4A in Multimedia Appendix 2). To address this, we used an optimal transport method, aligning the embeddings from all providers to the 2D embedding of a selected reference provider (Figure S4B in Multimedia Appendix 2). Following this alignment, hierarchical agglomerative clustering was applied to the adjusted note embeddings, revealing 3 distinct clusters, as determined by the silhouette score. The adjusted UMAP projection, color coded by cluster, is presented in Figure 5A. The patient distribution within these clusters was as follows: cluster 1 included 1280 (37.06%) patients, cluster 2 included 1161 (33.61%) patients, and cluster 3 included 1013 (29.33%) patients. Detailed summary statistics for each cluster are outlined in Table 3.

**Table 3:** Summary statistics of note clusters.

Characteristics			Total (N=3454)		Cluster 1 (n=1280)		Cluster 2 (n=1161)		Cluster 3 (n=1013)
Age of onset^a^ (y), mean (SD)			72.1 (9.5)		71.5 (9.3)		72.8 (9.2)		71.9 (9.8)
**Sex, n (%)**									
	Female	1678 (48.58)		812 (63.44)		327 (28.17)		539 (53.21)	
	Male	1776 (51.42)		468 (36.56)		834 (71.83)		474 (46.79)	
**Race, n (%)**									
	White	3059 (88.56)		1129 (88.20)		1032 (88.89)		898 (88.56)	
	Black or African American	77 (2.23)		30 (2.34)		26 (2.24)		21 (2.07)	
	Asian	90 (2.61)		36 (2.81)		25 (2.15)		29 (2.86)	
	American Indian or Alaska Native	4 (0.12)		1 (0.08)		1 (0.09)		2 (0.2)	
	Native Hawaiian or Other Pacific Islander	1 (0.03)		0 (0)		0 (0)		1 (0.1)	
	Other	103 (2.98)		34 (2.66)		44 (3.79)		25 (2.47)	
	Unavailable	120 (3.47)		50 (3.91)		33 (2.84)		37 (3.65)	
**Ethnicity, n (%)**									
	Not Hispanic or Latino	3020 (87.43)		1128 (88.13)		1004 (86.48)		888 (87.66)	
	Hispanic or Latino	106 (3.07)		36 (2.81)		39 (3.36)		31 (3.06)	
	Unavailable	328 (9.5)		116 (9.06)		118 (10.16)		94 (9.28)	
**ADRD Dx^b^, n (%)**									
	AD^c^	1317 (38.13)		519 (40.55)		435 (37.47)		363 (35.83)	
	Dementia unspecified	1101 (31.88)		389 (30.39)		381 (32.82)		331 (32.68)	
	FTD^d^	190 (5.5)		69 (5.39)		65 (5.6)		56 (5.53)	
	LBD^e^	261 (7.56)		81 (6.33)		104 (8.96)		76 (7.5)	
	VCI^f^	96 (2.78)		36 (2.81)		40 (3.45)		20 (1.97)	
	Other^g^	489 (14.16)		186 (14.53)		136 (11.71)		167 (16.49)	

^a^Age of onset was manually annotated by experts viewing clinical notes; for notes without age of onset, we approximated with the age of first encounter.

^b^Dx: diagnosis.

^c^AD: Alzheimer disease.

^d^FTD: frontotemporal dementia.

^e^LBD: Lewy body dementia.

^f^VCI: vascular cognitive impairment.

^g^Includes posterior.

We extracted common topics from each note cluster using topic modeling and examined the distribution of ADRD diagnoses across these clusters. In cluster 1, we found more terms related to psychiatric manifestations (eg, compulsive, indifference, and anxiety) and medications (eg, clozaril, trazodone, and sertraline), with a slight but nonsignificant enrichment in AD diagnosis (*χ*^2^_1_=4.9; *P*_FDR_=.14; PR=1.10). Cluster 2 had more terms related to cardiovascular issues (eg, pacemaker, hypotension, and arrhythmia) and motor and sensory issues (eg, slurring, cochlear, and tremors), with a slight but nonsignificant enrichment in LBD (*χ*^2^_1_=4.6; *P*_FDR_=.14; PR=1.31) and VCI (*χ*^2^_1_=2.5; *P*_FDR_=.25; PR=1.41) diagnoses. Cluster 3 encompassed a wide variety of symptoms and conditions common in geriatric populations, including autoimmune (eg, scleroderma and vasculitis), behavioral changes and movement (eg, usual jocular behavior and imbalance, dysphagia), sleep (eg, insomnia), and sensory (eg, diplopia) problems, with a slight but not-significant enrichment in rare ADRD diagnoses (*χ*^2^_1_=3; *P*_FDR_=.12; PR=1.25). Figure 3B depicts the list of representative words from each cluster and their PR, and Figure S5D in Multimedia Appendix 2 illustrates sentence examples from each cluster.

Furthermore, statistical analyses revealed significant associations of note cluster membership, with demographic variables, but not with ADRD diagnoses. First, both cluster 1 and 3 were significantly enriched by female participants (cluster 1: *χ*^2^_1_=178.7; *P*_FDR_<.001; PR=1.593 and cluster 3: *χ*^2^_1_=12; *P*_FDR_<.001; PR=1.14) while cluster 2 was enriched by male participants (*χ*^2^_1_=290.5; *P*_FDR_<.001; PR=1.749; Figure 5B). In addition, age of onset varied significantly among the note clusters (Kruskal-Wallis *χ*^2^_2_=14.9; *P*<.001; *η^2^*=0.004), with cluster 2 (mean 72.8, SD 9.2 years) having a significantly later age of onset compared to cluster 1 (*Z*=−3.82; *P*_FDR_<.001) and cluster 3 (*Z*=2.31; *P*_FDR_=.03), while cluster 1 (mean 71.5, SD 9.3 years) and cluster 3 (mean 71.9, SD 9.8 years) did not differ (*Z*=−1.33; *P*_FDR_=.19; Figure 5C). However, no association was observed between note cluster membership and ADRD diagnoses (Cluster 1-AD: *χ*^2^_1_=4.9; *P*_FDR_=.14; Cluster 1-dementia unspecified: *χ*^2^_1_=1.9; *P*_FDR_=.32; Cluster 1-FTD: *χ*^2^_1_=0.02; *P*_FDR_=.99; Cluster 1-LBD: *χ*^2^_1_=4.1; *P*_FDR_=.15; Cluster 1-other: *χ*^2^_1_=0.19; *P*_FDR_=.92; Cluster 1-VCI: *χ*^2^_1_<0.001, *P*_FDR_=.99; Cluster 2-AD: *χ*^2^_1_=0.3; *P*_FDR_=.89; Cluster 2-dementia unspecified: *χ*^2^_1_=0.6; *P*_FDR_=.76; Cluster 2-FTD: *χ*^2^_1_=0.01; *P*_FDR_=.99; Cluster 2-LBD: *χ*^2^_1_=4.6; *P*_FDR_=.14; Cluster 2-other: *χ*^2^_1_=8.3; *P*_FDR_=.07; Cluster 2-VCI: *χ*^2^_1_=2.5; *P*_FDR_=.25; Cluster 3-AD: *χ*^2^_1_=3.1; *P*_FDR_=.21; Cluster 3-dementia unspecified: *χ*^2^_1_=0.4; *P*_FDR_=.89; Cluster 3-FTD: *χ*^2^_1_<0.001; *P*_FDR_=.99; Cluster 3-LBD: *χ*^2^_1_<0.001; *P*_FDR_=.99; Cluster 3-other: *χ*^2^_1_=6.1; *P*_FDR_=.12; Cluster 3-VCI: *χ*^2^_1_=3; *P*_FDR_=.21; Figure 5D). Additional visualizations of the UMAP projections colored by sex, age of onset, and ADRD diagnoses are provided in Figures S5A, S5B, and S5C, respectively, in Multimedia Appendix 2.

### Comparison Between ICD Clusters and Note Clusters

**Figure 6:**
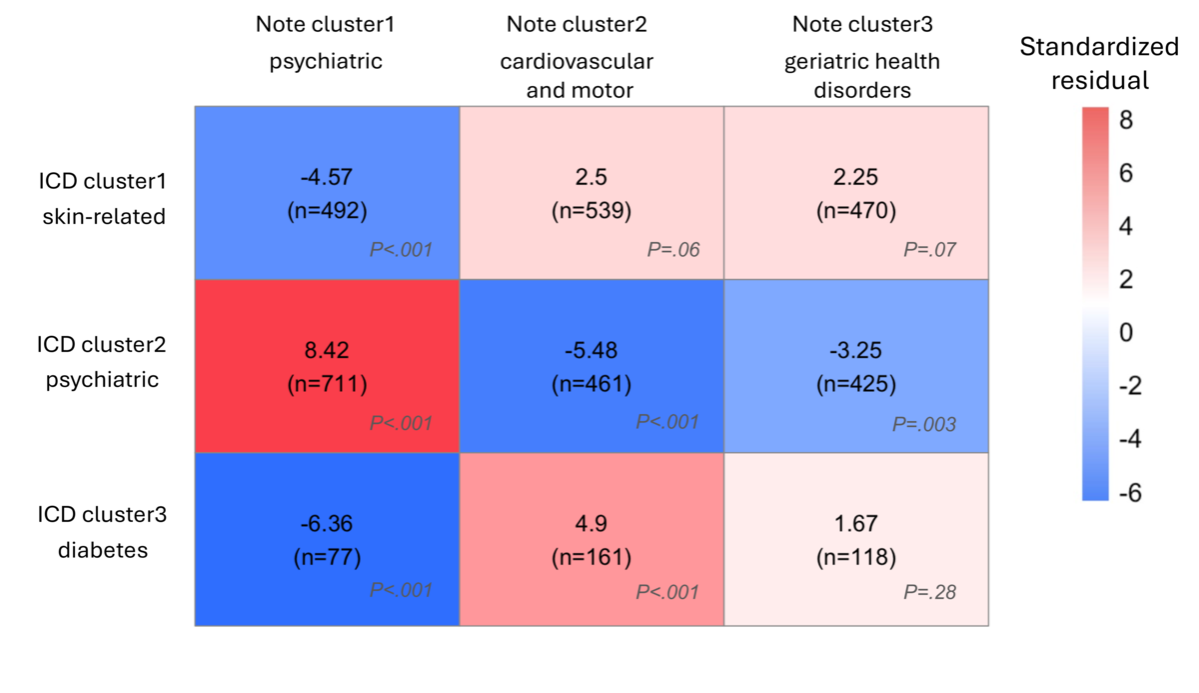
Heatmap of the association of International Classification of Diseases (ICD) clusters with note clusters. Heatmap of the association of ICD clusters with note clusters. ICD clusters were significantly associated with note clusters (*P*<.001). Post hoc analyses revealed that note cluster 1 was positively associated with ICD cluster 2 (standardized residual=8.42, *P*<.001) and negatively associated with ICD clusters 1 and 3 (cluster 1: standardized residual=−4.57, *P*<.001; cluster 3: standardized residual=−6.36, *P*<.001). Furthermore, note cluster 2 was positively associated with ICD cluster 3 (standardized residual=4.9, *P*<.001) and negatively associated with ICD cluster 2 (standardized residual=−5.48, *P*<.001). Finally, note cluster 3 was negatively associated with ICD cluster 2 (standardized residual=−3.25, *P*=.003). Each cell displays standardized residuals (standardized differences between the observed count and the expected count) along with the count of overlapping patients (n). Color bar represents the value of the standardized residual. *P* values were corrected with false discovery rate adjustments.

Statistical analysis demonstrated significant associations between *ICD* and note clusters (*χ*^2^_4_=89.43; *P*<.001; Cramér *V*=0.114). Specifically, note cluster 1, characterized by more female participants (PR=1.593) and terms related to psychiatric manifestations and medications, significantly overlapped (standardized residual=8.42; *P*_FDR_<.001) with *ICD* cluster 2, which is noted for the earliest onset of disease (mean 69.8, SD 9.6 years) and a higher prevalence of psychiatric disorders and higher proportion of patients with FTD (PR*=*2.039). In addition, note cluster 2, which had higher proportion of male participants (PR*=*1.749) and terms related to cardiovascular and motor issues, overlapped significantly (standardized residual=4.90; *P*_FDR_<.001) with *ICD* cluster 3, which is marked by the oldest onset of disease (mean 75.5, SD 8.1 years), a higher occurrence of VCI (PR*=*2.147), and dementia unspecified (PR*=*1.622) and had high prevalence of diabetes. These findings suggest a meaningful pattern of cluster correspondence across modalities (Figure 6).

**Figure 9:**
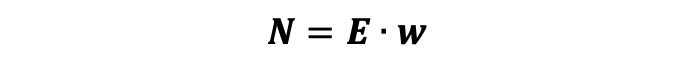
Inline graphic 3.

## Discussion

### Principal Findings

This study aimed to characterize the clinical heterogeneity across ADRD by applying representation learning techniques on patient EHRs in a tertiary care clinic. We used pretrained *ICD* code embeddings, a transformer architecture for encoding clinical notes, and unsupervised learning to identify distinct ADRD subtypes. This work represents the first example of clustering patients with ADRD using embeddings derived directly from an LLM, without prior rule-based extraction of relevant medical concepts from the clinical note. The *ICD* codes allowed us to investigate subtypes of patients with similar *ICD* codes (non-ADRD diagnosis in charts), while the clinical notes allowed us to capture clinical history and presentation recorded by memory specialists. Our results demonstrate distinct patterns of disease manifestation with significant overlap between the *ICD* and note clusters. The overlap of clusters between the 2 approaches suggests that the subtypes may reflect common underlying clinical heterogeneity, as distinct subtypes can be identified through different data modalities.

Our choice of hierarchical agglomerative clustering was guided by the hierarchical nature of our data, empirical evidence from prior dementia subtyping studies, and theoretical limitations of alternative algorithms. Alternative methods, such as K-means, Gaussian mixture models [40], and density-based spatial clustering of applications with noise (DBSCAN) [41], face theoretical limitations: K-means assumes spherical clusters that is difficult to satisfy in high-dimensional embeddings; DBSCAN relies on density thresholds that break down in such spaces; and Gaussian mixture models can be unstable with overlapping subtypes. In contrast, hierarchical clustering preserves these nested relationships, resulting in more homogeneous [42] and reproducible [43] clusters.

In our study, we identified 3 clusters from *ICD* embeddings, each characterized by distinct health conditions. Cluster 1 predominantly featured issues related to skin health, with a commonality of dementia unspecified diagnoses and an average age of onset in the early 70s. The association between skin health and dementia in this cluster may be attributed to age, as both conditions become more prevalent with advancing age. This aligns with findings from previous studies, which reported an increase in the prevalence of actinic keratosis [44] and seborrheic keratosis [45], as well as AD [46], with age. Cluster 2 is marked by early-onset, psychiatric and behavioral manifestations; enrichment in FTD and other less common forms of ADRD; and the earliest age of onset among our clusters, typically in the late 60s. This cluster extends the characterization found in previous studies that described a behavioral symptom subtype in patients [20,21] by demonstrating similar characteristics in a broader population of patients with ADRD. Cluster 3 encompasses a broad array of conditions like respiratory issues and severe diabetes, affecting older patients, more female participants than male participants, with a higher incidence of VCI and dementia unspecified, and an age of onset in the mid-70s. Reflecting the AD subtype identified by previous researchers, this group was characterized as being overall older and having more comorbidities [20]. Landi et al [23] further differentiated patients with AD by onset timing, which we also observed but across a more diverse set of ADRD diagnoses. Notably, our *ICD*-based clustering did not reveal clearly separated clusters in the 2D UMAP projections. While UMAP aims to preserve both local and global relationships when reducing high-dimensional data to a lower-dimensional space, some distortions may inevitably occur during dimensionality reduction. Alternatively, the overlapping clusters could reflect the complexity of comorbid conditions in ADRD, which may not form clearly distinguishable subgroups.

In addition, our analysis of clinical notes revealed 3 distinct subtypes. Cluster 1 featured terms related to psychiatric manifestations and medications, aligning with findings from previous studies [20,21]. Cluster 2 included terms related to cardiovascular and various motor and sensory issues, supported by previous studies that identified subtypes of CVD [20,21] and aligning with the predominant diagnoses of VCI and LBD within this cluster. Cluster 3 covered a wide array of health conditions, consistent with the higher occurrence of rare ADRD diagnoses, which tend to involve more heterogeneous health conditions. Notably, we observed significant overlap between *ICD* and note clusters, identifying 2 ADRD subtypes of interest that were concordant across the 2 data modalities: the first subtype, “psychiatric manifestations,” and the second subtype, “diabetes with cardiovascular or motor issues.” Thus, our analysis delineated 2 distinct ADRD subtypes with specific diagnostic and symptomatic profiles.

Our study identified sex differences across all clinical note clusters with substantial effects observed in note clusters 1 and 2. For example, note cluster 1 was significantly overrepresented in female participants and had a higher prevalence of AD (PR=1.1). It also overlapped significantly (*P*<.001) with *ICD* cluster 2, which was enriched for psychiatric and behavioral symptoms (eg, apathy). This aligns with Tang et al [25], who reported stronger psychiatric associations in female patients with AD, including greater links to depression. This pattern may be partially attributed to women’s greater likelihood of seeking mental health care [47,48]. In contrast, note cluster 2 was overrepresented in male participants, with higher prevalence of VCI (PR=1.41) and LBD (PR=1.31), consistent with Tang et al [25], who found vascular dementia was more common in male participants. The higher prevalence of VCI in male participants may be related to a greater burden of hypertension, particularly in early life [49]. In addition, the increased representation of male participants with LBD may reflect potential underdiagnosis in female participants [50,51]. Given the clinical impact of sex disparities in ADRD—particularly in AD and LBD [52]—future studies integrating longitudinal data and clinicopathological evidence will be crucial to disentangling biological influences from health care–seeking behaviors.

Another interesting observation relates to variations in age of onset, a key indicator of disease severity, across the identified subtypes in both clinical notes and *ICD*-based clusters, with notable differences in the *ICD*-derived subtypes. For instance, the early-onset *ICD* cluster 2 was enriched with psychiatric disorders and included diseases known with early-onset, including FTD [53] and other rare ADRD categories, such as PCA [54]. In contrast, the late-onset *ICD* cluster 3, exhibited a higher prevalence of diverse health conditions, including respiratory issues (eg, cough and shortness of breath), chronic pain, musculoskeletal conditions, such as bilateral low back pain and foot pain, as well as diabetes mellitus. While chronic pain is not typically associated with ADRD, pain could indicate the general aging process [55], and relate to osteoporosis and osteoarthritis, which likely contribute to chronic pain in older adults. Other symptoms, such as foot pain and shortness of breath, may reflect comorbidities of diabetes.

### Limitations

Our study has a few limitations. First, there are the challenges associated with using real-world EHR data. Differences in how health care providers document information, stemming from variations in training, personal documentation habits, and clinical judgment, may have contributed to inconsistencies. Furthermore, health care use patterns, such as visit regularity, may influence our clustering results. For example, variations in visit frequency could lead to overrepresentation of certain symptom clusters or skewed associations. Future studies adjusting for health care use patterns may help address this limitation. Second, the inclusion of long-term patient histories in clinical notes—where recent notes may capture both current and past symptoms—could introduce extraneous information, making it difficult to isolate content relevant to the latest diagnosis. This mixture of historical and recent data may have diluted the association between documented ADRD diagnoses and their actual clinical significance, leading to observed trends rather than clear associations. Furthermore, the repetition of relevant language across multiple encounters may have influenced the clustering process, potentially reflecting the frequency of patient visits to the memory clinic, rather than clinical characteristics. Third, our study is constrained by the absence of an independent validation cohort to confirm the identified clusters. While the relative overlap in clusters identified through *ICD* codes and note contents, along with the alignment with findings from previous research, offers some validation, the results could be strengthened by applying the same encoding and clustering techniques to an external validation cohort. To ensure external validity, these results need to be validated at other health care institutions. Fourth, another limitation of this study is that our subtyping characterization only focused on ADRD diagnoses based on etiology, but did not address the stage of disease, which clearly affects the neuropsychological profile. This calls for a focus on the heterogeneity of disease stage in future research. Moreover, there may be sex differences in who receives health care at different stages and ages, adding another layer of complexity to our findings. Finally, our study is limited by the lack of racial diversity in the cohort, with 88.6% of participants being White. Given known racial differences in AD incidence, comorbidities, and health care access [56-59]—and particularly the heightened impact of hypertension on AD risk in some minoritized groups [59,60]—our findings may not fully capture the spectrum of ADRD subtypes in these populations. Future studies with broader representation are necessary to improve the generalizability of our subtyping approach.

### Future Directions

In future work, the preprocessing of clinical notes could be enhanced by implementing multiple methods, such as medspaCy [61], with a focus on targeting sections most relevant to diagnoses, such as medical history. To further improve the extraction and analysis of pertinent data, the use of emerging LLMs, such as GPT [39], should be explored. In addition, validating an independent dataset and enriching the patient population would help increase the robustness and reliability of the identified ADRD subtypes. To advance this work, we will use a dual-modality approach that leverages both structured and unstructured data sources, such as medications and imaging. A deep autoencoder that uses multiple modalities simultaneously could offer methodological improvements over our current practice of conducting parallel clustering analyses and relying on heuristic averaging of embeddings. Furthermore, explicitly using the temporal or graph properties of EHRs could yield more informative representations, enhancing unsupervised clustering capabilities, as has been shown in prior approaches in a supervised learning setting [62,63]. Ultimately, our goal is to develop machine learning models capable of predicting these ADRD subtypes from real-word health care systems. Such models may aid in more precise diagnostics, prognostics, and the formulation of targeted treatment strategies.

## Appendix

Multimedia Appendix 1 List of diagnosis names to Alzheimer disease and related dementias diagnosis categories. This document provides a list of diagnosis names from the electronic health records of patients at a memory clinic to various Alzheimer disease and related dementias diagnosis categories.

Multimedia Appendix 2 Visualizations of model attention and embedding representations. This appendix includes attention heatmaps from the final transformer layer across heads, as well as Uniform Manifold Approximation and Projection projections of International Classification of Diseases code, and clinical note embeddings, characterized by sex, age of onset, Alzheimer disease and related dementia diagnosis, and provider information.

## Abbreviations

AD: Alzheimer disease
ADRD: Alzheimer disease and related dementia
Aβ: amyloid beta
BERT: Bidirectional Encoder Representations from Transformers
CVD: cerebrovascular disease
DBSCAN: density-based spatial clustering of applications with noise
EHR: electronic health record
FDR: false discovery rate
FTD: frontotemporal dementia
ICD: International Classification of Diseases
LBD: Lewy body dementia
LLM: large language model
MGH: Massachusetts General Hospital
MIMIC: Medical Information Mart for Intensive Care
PCA: posterior cortical atrophy
PR: prevalence ratio
TF-IDF: term frequency–inverse document frequency
UMAP: Uniform Manifold Approximation and Projection
VCI: vascular cognitive impairment

## Data Availability

The data used for this study are available from the Mass General Brigham Healthcare System, but restrictions apply to the availability of these data, which were used under permission for this study, and so are not publicly available. Code will be available on GitHub upon publication.
