# Supplementary material for: Unsupervised Deep Learning of Electronic Health Records to Characterize Heterogeneity Across Alzheimer Disease and Related Dementias: Cross-Sectional Study
Source: JMIR Aging. 2025 Mar 31;8:e65178. doi: 10.2196/65178 (PMC11997524; doi:10.2196/65178)
Supplement: Multimedia Appendix 1 [file aging_v8i1e65178_app1.pdf]

No.	Category	DiagnosisNM	CurrentICD9ListTXT	CurrentICD10ListTXT	ICD9CD	ICD10CD
1	AD	Alzheimer disease	331	G30.9, F02.80	331	F02.80
2	AD	Alzheimer disease	331	G30.9, F02.80	331	G30.9
3	AD	Alzheimer disease	331	G30.9, F02.80	331	F02.80
4	AD	Alzheimer disease	331	G30.9, F02.80	331	G30.9
5	AD	Alzheimer disease type 3	331	G30.0, F02.80	331	F02.80
6	AD	Alzheimer disease type 3	331	G30.0, F02.80	331	G30.0
7	AD	Alzheimer's dementia	331.0, 294.10	G30.9, F02.80	331	F02.80
8	AD	Alzheimer's dementia	331.0, 294.10	G30.9, F02.80	294.1	F02.80
9	AD	Alzheimer's dementia	331.0, 294.10	G30.9, F02.80	331	G30.9
10	AD	Alzheimer's dementia	331.0, 294.10	G30.9, F02.80	294.1	G30.9
11	AD	Alzheimer's dementia of other onset with behavioral disturbance	331.0, 294.11	G30.8, F02.818	331	F02.818
12	AD	Alzheimer's dementia of other onset with behavioral disturbance	331.0, 294.11	G30.8, F02.818	294.11	F02.818
13	AD	Alzheimer's dementia of other onset with behavioral disturbance	331.0, 294.11	G30.8, F02.818	331	G30.8
14	AD	Alzheimer's dementia of other onset with behavioral disturbance	331.0, 294.11	G30.8, F02.818	294.11	G30.8
15	AD	Alzheimer's dementia of other onset, with agitation, unspecified dementia severity	331.0, 294.11	G30.8, F02.811	331	F02.811
16	AD	Alzheimer's dementia of other onset, with agitation, unspecified dementia severity	331.0, 294.11	G30.8, F02.811	294.11	F02.811
17	AD	Alzheimer's dementia of other onset, with agitation, unspecified dementia severity	331.0, 294.11	G30.8, F02.811	331	G30.8
18	AD	Alzheimer's dementia of other onset, with agitation, unspecified dementia severity	331.0, 294.11	G30.8, F02.811	294.11	G30.8
19	AD	Alzheimer's dementia of other onset, with psychotic disturbance, unspecified dementia severity	331.0, 294.11	G30.8, F02.82	331	F02.82
20	AD	Alzheimer's dementia of other onset, with psychotic disturbance, unspecified dementia severity	331.0, 294.11	G30.8, F02.82	294.11	F02.82
21	AD	Alzheimer's dementia of other onset, with psychotic disturbance, unspecified dementia severity	331.0, 294.11	G30.8, F02.82	331	G30.8
22	AD	Alzheimer's dementia of other onset, with psychotic disturbance, unspecified dementia severity	331.0, 294.11	G30.8, F02.82	294.11	G30.8
23	AD	Alzheimer's dementia with behavioral disturbance	331.0, 294.11	G30.9, F02.818	331	F02.818
24	AD	Alzheimer's dementia with behavioral disturbance	331.0, 294.11	G30.9, F02.818	294.11	F02.818
25	AD	Alzheimer's dementia with behavioral disturbance	331.0, 294.11	G30.9, F02.818	331	G30.9
26	AD	Alzheimer's dementia with behavioral disturbance	331.0, 294.11	G30.9, F02.818	294.11	G30.9
27	AD	Alzheimer's dementia with behavioral disturbance, unspecified timing of dementia onset	331.0, 294.11	G30.9, F02.81	331	F02.81
28	AD	Alzheimer's dementia with behavioral disturbance, unspecified timing of dementia onset	331.0, 294.11	G30.9, F02.81	294.11	F02.81
29	AD	Alzheimer's dementia with behavioral disturbance, unspecified timing of dementia onset	331.0, 294.11	G30.9, F02.81	331	G30.9
30	AD	Alzheimer's dementia with behavioral disturbance, unspecified timing of dementia onset	331.0, 294.11	G30.9, F02.81	294.11	G30.9
31	AD	Alzheimer's dementia without behavioral disturbance	331.0, 294.10	G30.9, F02.80	331	F02.80
32	AD	Alzheimer's dementia without behavioral disturbance	331.0, 294.10	G30.9, F02.80	294.1	F02.80
33	AD	Alzheimer's dementia without behavioral disturbance	331.0, 294.10	G30.9, F02.80	331	G30.9
34	AD	Alzheimer's dementia without behavioral disturbance	331.0, 294.10	G30.9, F02.80	294.1	G30.9
35	AD	Alzheimer's dementia without behavioral disturbance, unspecified timing of dementia onset	331.0, 294.10	G30.9, F02.80	331	F02.80
36	AD	Alzheimer's dementia without behavioral disturbance, unspecified timing of dementia onset	331.0, 294.10	G30.9, F02.80	294.1	F02.80
37	AD	Alzheimer's dementia without behavioral disturbance, unspecified timing of dementia onset	331.0, 294.10	G30.9, F02.80	331	G30.9
38	AD	Alzheimer's dementia without behavioral disturbance, unspecified timing of dementia onset	331.0, 294.10	G30.9, F02.80	294.1	G30.9
39	AD	Alzheimer's dementia, late onset	331	G30.1, F02.80	331	F02.80
40	AD	Alzheimer's dementia, late onset	331	G30.1, F02.80	331	G30.1
41	AD	Alzheimer's dementia, late onset, with behavioral disturbance	331.0, 294.11	G30.1, F02.818	331	F02.818
42	AD	Alzheimer's dementia, late onset, with behavioral disturbance	331.0, 294.11	G30.1, F02.818	294.11	F02.818

No.	Category	DiagnosisNM	CurrentICD9ListTXT	CurrentICD10ListTXT	ICD9CD	ICD10CD
43	AD	Alzheimer's dementia, late onset, with behavioral disturbance	331.0, 294.11	G30.1, F02.818	331	G30.1
44	AD	Alzheimer's dementia, late onset, with behavioral disturbance	331.0, 294.11	G30.1, F02.818	294.11	G30.1
45	AD	Alzheimer's dementia, unspecified dementia severity, unspecified timing of dementia onset, unspecified whether behavioral, psychotic, or mood disturbance or anxiety	331.0, 294.10	G30.9, F02.80	331	G30.9
46	AD	Alzheimer's dementia, unspecified dementia severity, unspecified timing of dementia onset, unspecified whether behavioral, psychotic, or mood disturbance or anxiety	331.0, 294.10	G30.9, F02.80	294.1	G30.9
47	AD	Alzheimer's dementia, unspecified dementia severity, unspecified timing of dementia onset, unspecified whether behavioral, psychotic, or mood disturbance or anxiety	331.0, 294.10	G30.9, F02.80	331	F02.80
48	AD	Alzheimer's dementia, unspecified dementia severity, unspecified timing of dementia onset, unspecified whether behavioral, psychotic, or mood disturbance or anxiety	331.0, 294.10	G30.9, F02.80	294.1	F02.80
49	AD	Alzheimer's disease	331	G30.9, F02.80	331	F02.80
50	AD	Alzheimer's disease	331	G30.9, F02.80	331	G30.9
51	AD	Alzheimer's disease	NULL	G30	NULL	G30
52	AD	Alzheimer's disease	331	G30.9, F02.80	331	G30.9
53	AD	Alzheimer's disease	331	G30.9, F02.80	331	F02.80
54	AD	Alzheimer's disease associated with mutation in APOE gene	331	G30.9, F02.80	331	F02.80
55	AD	Alzheimer's disease associated with mutation in APOE gene	331	G30.9, F02.80	331	G30.9
56	AD	Alzheimer's disease of other onset	331	G30.8, F02.80	331	F02.80
57	AD	Alzheimer's disease of other onset	331	G30.8, F02.80	331	G30.8
58	AD	Alzheimer's disease of other onset with behavioral disturbance	331.0, 294.11	G30.8, F02.818	331	F02.818
59	AD	Alzheimer's disease of other onset with behavioral disturbance	331.0, 294.11	G30.8, F02.818	294.11	F02.818
60	AD	Alzheimer's disease of other onset with behavioral disturbance	331.0, 294.11	G30.8, F02.818	331	G30.8
61	AD	Alzheimer's disease of other onset with behavioral disturbance	331.0, 294.11	G30.8, F02.818	294.11	G30.8
62	AD	Alzheimer's disease of other onset without behavioral disturbance	331.0, 294.10	G30.8, F02.80	331	F02.80
63	AD	Alzheimer's disease of other onset without behavioral disturbance	331.0, 294.10	G30.8, F02.80	294.1	F02.80
64	AD	Alzheimer's disease of other onset without behavioral disturbance	331.0, 294.10	G30.8, F02.80	331	G30.8
65	AD	Alzheimer's disease of other onset without behavioral disturbance	331.0, 294.10	G30.8, F02.80	294.1	G30.8
66	AD	Alzheimer's disease with behavioral disturbance	331.0, 294.11	G30.9, F02.818	331	F02.818
67	AD	Alzheimer's disease with behavioral disturbance	331.0, 294.11	G30.9, F02.818	294.11	F02.818
68	AD	Alzheimer's disease with behavioral disturbance	331.0, 294.11	G30.9, F02.818	331	G30.9
69	AD	Alzheimer's disease with behavioral disturbance	331.0, 294.11	G30.9, F02.818	294.11	G30.9
70	AD	Alzheimer's disease with behavioral disturbance	331.0, 294.11	G30.9, F02.818	294.11	F02.818
71	AD	Alzheimer's disease with behavioral disturbance	331.0, 294.11	G30.9, F02.818	331	F02.818
72	AD	Alzheimer's disease with behavioral disturbance	331.0, 294.11	G30.9, F02.818	331	G30.9
73	AD	Alzheimer's disease with behavioral disturbance	331.0, 294.11	G30.9, F02.818	294.11	G30.9
74	AD	Alzheimer's disease with late onset	NULL	G30.1	NULL	G30.1
75	AD	Alzheimer's disease with late onset	331.0, 294.10	G30.1, F02.80	331	F02.80
76	AD	Alzheimer's disease with late onset	331.0, 294.10	G30.1, F02.80	294.1	F02.80
77	AD	Alzheimer's disease with late onset	331.0, 294.10	G30.1, F02.80	331	G30.1
78	AD	Alzheimer's disease with late onset	331.0, 294.10	G30.1, F02.80	294.1	G30.1
79	AD	Alzheimer's disease with late onset	331.0, 294.10	G30.1, F02.80	294.1	G30.1
80	AD	Alzheimer's disease with late onset	331.0, 294.10	G30.1, F02.80	294.1	F02.80
81	AD	Alzheimer's disease with late onset	331.0, 294.10	G30.1, F02.80	331	F02.80
82	AD	Alzheimer's disease with late onset	331.0, 294.10	G30.1, F02.80	331	G30.1
83	AD	Alzheimer's disease with late onset (CODE)	331.0, 294.10	G30.1	331	G30.1
84	AD	Alzheimer's disease with late onset (CODE)	331.0, 294.10	G30.1	294.1	G30.1

No.	Category	DiagnosisNM	CurrentICD9ListTXT	CurrentICD10ListTXT	ICD9CD	ICD10CD
85	AD	Alzheimer's disease, unspecified	331	G30.9, F02.80	331	F02.80
86	AD	Alzheimer's disease, unspecified	331	G30.9, F02.80	331	G30.9
87	AD	Alzheimer's disease, unspecified	NULL	G30.9	NULL	G30.9
88	AD	Alzheimer's type dementia	331.0, 294.10	G30.9, F02.80	331	F02.80
89	AD	Alzheimer's type dementia	331.0, 294.10	G30.9, F02.80	294.1	F02.80
90	AD	Alzheimer's type dementia	331.0, 294.10	G30.9, F02.80	331	G30.9
91	AD	Alzheimer's type dementia	331.0, 294.10	G30.9, F02.80	294.1	G30.9
92	AD	Alzheimer's type dementia with late onset with behavioral disturbance	331.0, 294.11	G30.1, F02.818	331	F02.818
93	AD	Alzheimer's type dementia with late onset with behavioral disturbance	331.0, 294.11	G30.1, F02.818	294.11	F02.818
94	AD	Alzheimer's type dementia with late onset with behavioral disturbance	331.0, 294.11	G30.1, F02.818	331	G30.1
95	AD	Alzheimer's type dementia with late onset with behavioral disturbance	331.0, 294.11	G30.1, F02.818	294.11	G30.1
96	AD	Alzheimer's type dementia with late onset without behavioral disturbance	331.0, 294.10	G30.1, F02.80	331	F02.80
97	AD	Alzheimer's type dementia with late onset without behavioral disturbance	331.0, 294.10	G30.1, F02.80	294.1	F02.80
98	AD	Alzheimer's type dementia with late onset without behavioral disturbance	331.0, 294.10	G30.1, F02.80	331	G30.1
99	AD	Alzheimer's type dementia with late onset without behavioral disturbance	331.0, 294.10	G30.1, F02.80	294.1	G30.1
100	AD	Alzheimers disease	331	G30.9, F02.80	331	F02.80
101	AD	Alzheimers disease	331	G30.9, F02.80	331	G30.9
102	AD	Behavioral disturbance due to late onset Alzheimer dementia	331.0, 294.11	G30.1, F02.818	331	G30.1
103	AD	Behavioral disturbance due to late onset Alzheimer dementia	331.0, 294.11	G30.1, F02.818	294.11	G30.1
104	AD	Behavioral disturbance due to late onset Alzheimer dementia	331.0, 294.11	G30.1, F02.818	331	F02.818
105	AD	Behavioral disturbance due to late onset Alzheimer dementia	331.0, 294.11	G30.1, F02.818	294.11	F02.818
106	AD	Dementia due to Alzheimer's disease	331.0, 294.10	G30.9, F02.80	331	F02.80
107	AD	Dementia due to Alzheimer's disease	331.0, 294.10	G30.9, F02.80	294.1	F02.80
108	AD	Dementia due to Alzheimer's disease	331.0, 294.10	G30.9, F02.80	331	G30.9
109	AD	Dementia due to Alzheimer's disease	331.0, 294.10	G30.9, F02.80	294.1	G30.9
110	AD	Dementia in Alzheimer's disease	331.0, 294.10	G30.9, F02.80	331	F02.80
111	AD	Dementia in Alzheimer's disease	331.0, 294.10	G30.9, F02.80	294.1	F02.80
112	AD	Dementia in Alzheimer's disease	331.0, 294.10	G30.9, F02.80	331	G30.9
113	AD	Dementia in Alzheimer's disease	331.0, 294.10	G30.9, F02.80	294.1	G30.9
114	AD	Dementia in Alzheimer's disease with delusions	331.0, 294.10, 297.9	G30.9, F02.82	331	F02.82
115	AD	Dementia in Alzheimer's disease with delusions	331.0, 294.10, 297.9	G30.9, F02.82	294.1	F02.82
116	AD	Dementia in Alzheimer's disease with delusions	331.0, 294.10, 297.9	G30.9, F02.82	297.9	F02.82
117	AD	Dementia in Alzheimer's disease with delusions	331.0, 294.10, 297.9	G30.9, F02.82	331	G30.9
118	AD	Dementia in Alzheimer's disease with delusions	331.0, 294.10, 297.9	G30.9, F02.82	294.1	G30.9
119	AD	Dementia in Alzheimer's disease with delusions	331.0, 294.10, 297.9	G30.9, F02.82	297.9	G30.9
120	AD	Dementia in Alzheimer's disease with depression	331.0, 294.10, 311	G30.9, F02.83	331	F02.83
121	AD	Dementia in Alzheimer's disease with depression	331.0, 294.10, 311	G30.9, F02.83	294.1	F02.83
122	AD	Dementia in Alzheimer's disease with depression	331.0, 294.10, 311	G30.9, F02.83	311	F02.83
123	AD	Dementia in Alzheimer's disease with depression	331.0, 294.10, 311	G30.9, F02.83	331	G30.9
124	AD	Dementia in Alzheimer's disease with depression	331.0, 294.10, 311	G30.9, F02.83	294.1	G30.9
125	AD	Dementia in Alzheimer's disease with depression	331.0, 294.10, 311	G30.9, F02.83	311	G30.9
126	AD	Dementia in Alzheimer's disease with late onset	331.0, 294.10	G30.1, F02.80	331	F02.80

No.	Category	DiagnosisNM	CurrentICD9ListTXT	CurrentICD10ListTXT	ICD9CD	ICD10CD
127	AD	Dementia in Alzheimer's disease with late onset	331.0, 294.10	G30.1, F02.80	294.1	F02.80
128	AD	Dementia in Alzheimer's disease with late onset	331.0, 294.10	G30.1, F02.80	331	G30.1
129	AD	Dementia in Alzheimer's disease with late onset	331.0, 294.10	G30.1, F02.80	294.1	G30.1
130	AD	Dementia of the Alzheimer's type	331.0, 294.10	G30.9, F02.80	331	F02.80
131	AD	Dementia of the Alzheimer's type	331.0, 294.10	G30.9, F02.80	294.1	F02.80
132	AD	Dementia of the Alzheimer's type	331.0, 294.10	G30.9, F02.80	331	G30.9
133	AD	Dementia of the Alzheimer's type	331.0, 294.10	G30.9, F02.80	294.1	G30.9
134	AD	Dementia of the Alzheimer's type with early onset without behavioral disturbance	331.0, 294.10	G30.0, F02.80	331	F02.80
135	AD	Dementia of the Alzheimer's type with early onset without behavioral disturbance	331.0, 294.10	G30.0, F02.80	294.1	F02.80
136	AD	Dementia of the Alzheimer's type with early onset without behavioral disturbance	331.0, 294.10	G30.0, F02.80	331	G30.0
137	AD	Dementia of the Alzheimer's type with early onset without behavioral disturbance	331.0, 294.10	G30.0, F02.80	294.1	G30.0
138	AD	Dementia of the Alzheimer's type with late onset without behavioral disturbance	331.0, 294.10	G30.1, F02.80	331	F02.80
139	AD	Dementia of the Alzheimer's type with late onset without behavioral disturbance	331.0, 294.10	G30.1, F02.80	294.1	F02.80
140	AD	Dementia of the Alzheimer's type with late onset without behavioral disturbance	331.0, 294.10	G30.1, F02.80	331	G30.1
141	AD	Dementia of the Alzheimer's type with late onset without behavioral disturbance	331.0, 294.10	G30.1, F02.80	294.1	G30.1
142	AD	Dementia of the Alzheimer's type, with early onset, with depressive mood	331.0, 290.13	G30.0, F02.83	331	F02.83
143	AD	Dementia of the Alzheimer's type, with early onset, with depressive mood	331.0, 290.13	G30.0, F02.83	290.13	F02.83
144	AD	Dementia of the Alzheimer's type, with early onset, with depressive mood	331.0, 290.13	G30.0, F02.83	331	G30.0
145	AD	Dementia of the Alzheimer's type, with early onset, with depressive mood	331.0, 290.13	G30.0, F02.83	290.13	G30.0
146	AD	Dementia of the Alzheimer's type, with late onset, uncomplicated	331.0, 294.10	G30.1, F02.80	331	F02.80
147	AD	Dementia of the Alzheimer's type, with late onset, uncomplicated	331.0, 294.10	G30.1, F02.80	294.1	F02.80
148	AD	Dementia of the Alzheimer's type, with late onset, uncomplicated	331.0, 294.10	G30.1, F02.80	331	G30.1
149	AD	Dementia of the Alzheimer's type, with late onset, uncomplicated	331.0, 294.10	G30.1, F02.80	294.1	G30.1
150	AD	Dementia of the Alzheimer's type, with late onset, with delirium	331.0, 294.11	G30.1, F02.82	331	F02.82
151	AD	Dementia of the Alzheimer's type, with late onset, with delirium	331.0, 294.11	G30.1, F02.82	294.11	F02.82
152	AD	Dementia of the Alzheimer's type, with late onset, with delirium	331.0, 294.11	G30.1, F02.82	331	G30.1
153	AD	Dementia of the Alzheimer's type, with late onset, with delirium	331.0, 294.11	G30.1, F02.82	294.11	G30.1
154	AD	Dementia of the Alzheimer's type, with late onset, with delusions	331.0, 290.20	G30.1, F02.818	331	F02.818
155	AD	Dementia of the Alzheimer's type, with late onset, with delusions	331.0, 290.20	G30.1, F02.818	290.2	F02.818
156	AD	Dementia of the Alzheimer's type, with late onset, with delusions	331.0, 290.20	G30.1, F02.818	331	G30.1
157	AD	Dementia of the Alzheimer's type, with late onset, with delusions	331.0, 290.20	G30.1, F02.818	290.2	G30.1
158	AD	Dementia of the Alzheimer's type, with late onset, with depressed mood	331.0, 294.10, 311	G30.1, F02.83	331	F02.83
159	AD	Dementia of the Alzheimer's type, with late onset, with depressed mood	331.0, 294.10, 311	G30.1, F02.83	294.1	F02.83
160	AD	Dementia of the Alzheimer's type, with late onset, with depressed mood	331.0, 294.10, 311	G30.1, F02.83	311	F02.83
161	AD	Dementia of the Alzheimer's type, with late onset, with depressed mood	331.0, 294.10, 311	G30.1, F02.83	331	G30.1
162	AD	Dementia of the Alzheimer's type, with late onset, with depressed mood	331.0, 294.10, 311	G30.1, F02.83	294.1	G30.1
163	AD	Dementia of the Alzheimer's type, with late onset, with depressed mood	331.0, 294.10, 311	G30.1, F02.83	311	G30.1
164	AD	Dementia of the Alzheimer's type, with late onset, with depressive mood	331.0, 294.10, 311	G30.1, F02.83	331	F02.83
165	AD	Dementia of the Alzheimer's type, with late onset, with depressive mood	331.0, 294.10, 311	G30.1, F02.83	294.1	F02.83
166	AD	Dementia of the Alzheimer's type, with late onset, with depressive mood	331.0, 294.10, 311	G30.1, F02.83	311	F02.83
167	AD	Dementia of the Alzheimer's type, with late onset, with depressive mood	331.0, 294.10, 311	G30.1, F02.83	331	G30.1
168	AD	Dementia of the Alzheimer's type, with late onset, with depressive mood	331.0, 294.10, 311	G30.1, F02.83	294.1	G30.1

No.	Category	DiagnosisNM	CurrentICD9ListTXT	CurrentICD10ListTXT	ICD9CD	ICD10CD
169	AD	Dementia of the Alzheimer's type, with late onset, with depressive mood	331.0, 294.10, 311	G30.1, F02.83	311	G30.1
170	AD	Dementia, Alzheimer's, with behavior disturbance	331.0, 294.11	G30.9, F02.818	331	F02.818
171	AD	Dementia, Alzheimer's, with behavior disturbance	331.0, 294.11	G30.9, F02.818	294.11	F02.818
172	AD	Dementia, Alzheimer's, with behavior disturbance	331.0, 294.11	G30.9, F02.818	331	G30.9
173	AD	Dementia, Alzheimer's, with behavior disturbance	331.0, 294.11	G30.9, F02.818	294.11	G30.9
174	AD	Depressed mood in Alzheimer's disease	331.0, 294.10, 799.29	G30.9, F02.80, R45.89	331	R45.89
175	AD	Depressed mood in Alzheimer's disease	331.0, 294.10, 799.29	G30.9, F02.80, R45.89	294.1	R45.89
176	AD	Depressed mood in Alzheimer's disease	331.0, 294.10, 799.29	G30.9, F02.80, R45.89	799.29	R45.89
177	AD	Depressed mood in Alzheimer's disease	331.0, 294.10, 799.29	G30.9, F02.80, R45.89	331	F02.80
178	AD	Depressed mood in Alzheimer's disease	331.0, 294.10, 799.29	G30.9, F02.80, R45.89	294.1	F02.80
179	AD	Depressed mood in Alzheimer's disease	331.0, 294.10, 799.29	G30.9, F02.80, R45.89	799.29	F02.80
180	AD	Depressed mood in Alzheimer's disease	331.0, 294.10, 799.29	G30.9, F02.80, R45.89	331	G30.9
181	AD	Depressed mood in Alzheimer's disease	331.0, 294.10, 799.29	G30.9, F02.80, R45.89	294.1	G30.9
182	AD	Depressed mood in Alzheimer's disease	331.0, 294.10, 799.29	G30.9, F02.80, R45.89	799.29	G30.9
183	AD	Late onset Alzheimer disease	331.0, 294.10	G30.1, F02.80	331	F02.80
184	AD	Late onset Alzheimer disease	331.0, 294.10	G30.1, F02.80	294.1	F02.80
185	AD	Late onset Alzheimer disease	331.0, 294.10	G30.1, F02.80	331	G30.1
186	AD	Late onset Alzheimer disease	331.0, 294.10	G30.1, F02.80	294.1	G30.1
187	AD	Late onset Alzheimer disease	331.0, 294.10	G30.1, F02.80	294.1	G30.1
188	AD	Late onset Alzheimer disease	331.0, 294.10	G30.1, F02.80	294.1	F02.80
189	AD	Late onset Alzheimer disease	331.0, 294.10	G30.1, F02.80	331	F02.80
190	AD	Late onset Alzheimer disease	331.0, 294.10	G30.1, F02.80	331	G30.1
191	AD	Late onset Alzheimer's dementia with behavioral disturbance	331.0, 294.11	G30.1, F02.818	331	F02.818
192	AD	Late onset Alzheimer's dementia with behavioral disturbance	331.0, 294.11	G30.1, F02.818	294.11	F02.818
193	AD	Late onset Alzheimer's dementia with behavioral disturbance	331.0, 294.11	G30.1, F02.818	331	G30.1
194	AD	Late onset Alzheimer's dementia with behavioral disturbance	331.0, 294.11	G30.1, F02.818	294.11	G30.1
195	AD	Late onset Alzheimer's dementia with other behavioral disturbance, unspecified dementia severity	331.0, 294.11	G30.1, F02.818	331	F02.818
196	AD	Late onset Alzheimer's dementia with other behavioral disturbance, unspecified dementia severity	331.0, 294.11	G30.1, F02.818	294.11	F02.818
197	AD	Late onset Alzheimer's dementia with other behavioral disturbance, unspecified dementia severity	331.0, 294.11	G30.1, F02.818	331	G30.1
198	AD	Late onset Alzheimer's dementia with other behavioral disturbance, unspecified dementia severity	331.0, 294.11	G30.1, F02.818	294.11	G30.1
199	AD	Late onset Alzheimer's dementia without behavioral disturbance	331.0, 294.10	G30.1, F02.80	331	F02.80
200	AD	Late onset Alzheimer's dementia without behavioral disturbance	331.0, 294.10	G30.1, F02.80	294.1	F02.80
201	AD	Late onset Alzheimer's dementia without behavioral disturbance	331.0, 294.10	G30.1, F02.80	331	G30.1
202	AD	Late onset Alzheimer's dementia without behavioral disturbance	331.0, 294.10	G30.1, F02.80	294.1	G30.1
203	AD	Late onset Alzheimer's dementia without behavioral disturbance, psychotic disturbance, mood disturbance, or anxiety, unspecified dementia severity	331.0, 294.10	G30.1, F02.80	294.1	G30.1
204	AD	Late onset Alzheimer's dementia without behavioral disturbance, psychotic disturbance, mood disturbance, or anxiety, unspecified dementia severity	331.0, 294.10	G30.1, F02.80	294.1	F02.80
205	AD	Late onset Alzheimer's dementia without behavioral disturbance, psychotic disturbance, mood disturbance, or anxiety, unspecified dementia severity	331.0, 294.10	G30.1, F02.80	331	F02.80
206	AD	Late onset Alzheimer's dementia without behavioral disturbance, psychotic disturbance, mood disturbance, or anxiety, unspecified dementia severity	331.0, 294.10	G30.1, F02.80	331	G30.1
207	AD	Late onset Alzheimer's disease with behavioral disturbance	331.0, 294.11	G30.1, F02.818	331	F02.818
208	AD	Late onset Alzheimer's disease with behavioral disturbance	331.0, 294.11	G30.1, F02.818	294.11	F02.818
209	AD	Late onset Alzheimer's disease with behavioral disturbance	331.0, 294.11	G30.1, F02.818	331	G30.1
210	AD	Late onset Alzheimer's disease with behavioral disturbance	331.0, 294.11	G30.1, F02.818	294.11	G30.1

No.	Category	DiagnosisNM	CurrentICD9ListTXT	CurrentICD10ListTXT	ICD9CD	ICD10CD
211	AD	Late onset Alzheimer's disease without behavioral disturbance	331.0, 294.10	G30.1, F02.80	331	F02.80
212	AD	Late onset Alzheimer's disease without behavioral disturbance	331.0, 294.10	G30.1, F02.80	294.1	F02.80
213	AD	Late onset Alzheimer's disease without behavioral disturbance	331.0, 294.10	G30.1, F02.80	331	G30.1
214	AD	Late onset Alzheimer's disease without behavioral disturbance	331.0, 294.10	G30.1, F02.80	294.1	G30.1
215	AD	Major neurocognitive disorder due to Alzheimer's disease	331.0, 294.10	G30.9, F02.80	331	F02.80
216	AD	Major neurocognitive disorder due to Alzheimer's disease	331.0, 294.10	G30.9, F02.80	294.1	F02.80
217	AD	Major neurocognitive disorder due to Alzheimer's disease	331.0, 294.10	G30.9, F02.80	331	G30.9
218	AD	Major neurocognitive disorder due to Alzheimer's disease	331.0, 294.10	G30.9, F02.80	294.1	G30.9
219	AD	Major neurocognitive disorder due to Alzheimer's disease, possible	294.2	F03.90	294.2	F03.90
220	AD	Major neurocognitive disorder due to Alzheimer's disease, probable, with behavioral disturbance	294.2	F03.91	294.2	F03.91
221	AD	Major neurocognitive disorder due to Alzheimer's disease, probable, without behavioral disturbance	294.2	F03.90	294.2	F03.90
222	AD	Major neurocognitive disorder due to possible Alzheimer's disease	294.2	F03.90	294.2	F03.90
223	AD	Major neurocognitive disorder due to possible Alzheimer's disease	294.2	F03.90	294.2	F03.90
224	AD	Major neurocognitive disorder due to possible Alzheimer's disease, without behavioral disturbance	294.2	F03.90	294.2	F03.90
225	AD	Major neurocognitive disorder due to possible frontotemporal lobar degeneration	294.2	F03.90	294.2	F03.90
226	AD	Major neurocognitive disorder due to possible frontotemporal lobar degeneration, without behavioral disturbance	294.2	F03.90	294.2	F03.90
227	AD	Major neurocognitive disorder due to probable Alzheimer's disease, without behavioral disturbance	294.2	F03.90	294.2	F03.90
228	AD	Major neurocognitive disorder, due to Alzheimer's disease, without behavioral disturbance, mild	331.0, 294.9	G30.9, F02.A0	331	F02.A0
229	AD	Major neurocognitive disorder, due to Alzheimer's disease, without behavioral disturbance, mild	331.0, 294.9	G30.9, F02.A0	294.9	F02.A0
230	AD	Major neurocognitive disorder, due to Alzheimer's disease, without behavioral disturbance, mild	331.0, 294.9	G30.9, F02.A0	331	G30.9
231	AD	Major neurocognitive disorder, due to Alzheimer's disease, without behavioral disturbance, mild	331.0, 294.9	G30.9, F02.A0	294.9	G30.9
232	AD	Major neurocognitive disorder, due to Alzheimer's disease, without behavioral disturbance, moderate	331.0, 294.9	G30.9, F02.B0	331	F02.B0
233	AD	Major neurocognitive disorder, due to Alzheimer's disease, without behavioral disturbance, moderate	331.0, 294.9	G30.9, F02.B0	294.9	F02.B0
234	AD	Major neurocognitive disorder, due to Alzheimer's disease, without behavioral disturbance, moderate	331.0, 294.9	G30.9, F02.B0	331	G30.9
235	AD	Major neurocognitive disorder, due to Alzheimer's disease, without behavioral disturbance, moderate	331.0, 294.9	G30.9, F02.B0	294.9	G30.9
236	AD	Mild Alzheimer's dementia without behavioral disturbance, psychotic disturbance, mood disturbance, or anxiety, unspecified timing of dementia onset	331.0, 294.10	G30.9, F02.A0	331	F02.A0
237	AD	Mild Alzheimer's dementia without behavioral disturbance, psychotic disturbance, mood disturbance, or anxiety, unspecified timing of dementia onset	331.0, 294.10	G30.9, F02.A0	294.1	F02.A0
238	AD	Mild Alzheimer's dementia without behavioral disturbance, psychotic disturbance, mood disturbance, or anxiety, unspecified timing of dementia onset	331.0, 294.10	G30.9, F02.A0	331	G30.9
239	AD	Mild Alzheimer's dementia without behavioral disturbance, psychotic disturbance, mood disturbance, or anxiety, unspecified timing of dementia onset	331.0, 294.10	G30.9, F02.A0	294.1	G30.9
240	AD	Mild late onset Alzheimer's dementia with anxiety	331.0, 294.11	G30.1, F02.A4	331	F02.A4
241	AD	Mild late onset Alzheimer's dementia with anxiety	331.0, 294.11	G30.1, F02.A4	294.11	F02.A4
242	AD	Mild late onset Alzheimer's dementia with anxiety	331.0, 294.11	G30.1, F02.A4	331	G30.1
243	AD	Mild late onset Alzheimer's dementia with anxiety	331.0, 294.11	G30.1, F02.A4	294.11	G30.1
244	AD	Mild late onset Alzheimer's dementia with other behavioral disturbance	331.0, 294.11	G30.1, F02.A18	331	F02.A18
245	AD	Mild late onset Alzheimer's dementia with other behavioral disturbance	331.0, 294.11	G30.1, F02.A18	294.11	F02.A18
246	AD	Mild late onset Alzheimer's dementia with other behavioral disturbance	331.0, 294.11	G30.1, F02.A18	331	G30.1
247	AD	Mild late onset Alzheimer's dementia with other behavioral disturbance	331.0, 294.11	G30.1, F02.A18	294.11	G30.1
248	AD	Mild late onset Alzheimer's dementia with psychotic disturbance	331.0, 294.11	G30.1, F02.A2	331	G30.1
249	AD	Mild late onset Alzheimer's dementia with psychotic disturbance	331.0, 294.11	G30.1, F02.A2	294.11	G30.1
250	AD	Mild late onset Alzheimer's dementia with psychotic disturbance	331.0, 294.11	G30.1, F02.A2	331	F02.A2
251	AD	Mild late onset Alzheimer's dementia with psychotic disturbance	331.0, 294.11	G30.1, F02.A2	294.11	F02.A2
252	AD	Mild late onset Alzheimer's dementia without behavioral disturbance, psychotic disturbance, mood disturbance, or anxiety	331.0, 294.10	G30.1, F02.A0	331	F02.A0

No.	Category	DiagnosisNM	CurrentICD9ListTXT	CurrentICD10ListTXT	ICD9CD	ICD10CD
253	AD	Mild late onset Alzheimer's dementia without behavioral disturbance, psychotic disturbance, mood disturbance, or anxiety	331.0, 294.10	G30.1, F02.A0	294.1	F02.A0
254	AD	Mild late onset Alzheimer's dementia without behavioral disturbance, psychotic disturbance, mood disturbance, or anxiety	331.0, 294.10	G30.1, F02.A0	331	G30.1
255	AD	Mild late onset Alzheimer's dementia without behavioral disturbance, psychotic disturbance, mood disturbance, or anxiety	331.0, 294.10	G30.1, F02.A0	294.1	G30.1
256	AD	Mild major neurocognitive disorder due to Alzheimer's disease with behavioral disturbance	331.0, 294.9	G30.9, F02.A18	331	F02.A18
257	AD	Mild major neurocognitive disorder due to Alzheimer's disease with behavioral disturbance	331.0, 294.9	G30.9, F02.A18	294.9	F02.A18
258	AD	Mild major neurocognitive disorder due to Alzheimer's disease with behavioral disturbance	331.0, 294.9	G30.9, F02.A18	331	G30.9
259	AD	Mild major neurocognitive disorder due to Alzheimer's disease with behavioral disturbance	331.0, 294.9	G30.9, F02.A18	294.9	G30.9
260	AD	Mild major neurocognitive disorder due to Alzheimer's disease without behavioral disturbance	331.0, 294.9	G30.9, F02.A0	331	F02.A0
261	AD	Mild major neurocognitive disorder due to Alzheimer's disease without behavioral disturbance	331.0, 294.9	G30.9, F02.A0	294.9	F02.A0
262	AD	Mild major neurocognitive disorder due to Alzheimer's disease without behavioral disturbance	331.0, 294.9	G30.9, F02.A0	331	G30.9
263	AD	Mild major neurocognitive disorder due to Alzheimer's disease without behavioral disturbance	331.0, 294.9	G30.9, F02.A0	294.9	G30.9
264	AD	Mild neurocognitive disorder due to Alzheimer's disease	331	G30.9, F06.70	331	F06.70
265	AD	Mild neurocognitive disorder due to Alzheimer's disease	331	G30.9, F06.70	331	G30.9
266	AD	Mild possible major neurocognitive disorder due to Alzheimer's disease	IMO0001	IMO0001	IMO0001	IMO0001
267	AD	Mild probable major neurocognitive disorder due to Alzheimer's disease without behavioral disturbance	294.2	F03.90	294.2	F03.90
268	AD	Mixed Alzheimer's and vascular dementia	331.0, 294.10, 290.40	G30.9, F01.50, F02.80	331	F02.80
269	AD	Mixed Alzheimer's and vascular dementia	331.0, 294.10, 290.40	G30.9, F01.50, F02.80	294.1	F02.80
270	AD	Mixed Alzheimer's and vascular dementia	331.0, 294.10, 290.40	G30.9, F01.50, F02.80	290.4	F02.80
271	AD	Mixed Alzheimer's and vascular dementia	331.0, 294.10, 290.40	G30.9, F01.50, F02.80	331	F01.50
272	AD	Mixed Alzheimer's and vascular dementia	331.0, 294.10, 290.40	G30.9, F01.50, F02.80	294.1	F01.50
273	AD	Mixed Alzheimer's and vascular dementia	331.0, 294.10, 290.40	G30.9, F01.50, F02.80	290.4	F01.50
274	AD	Mixed Alzheimer's and vascular dementia	331.0, 294.10, 290.40	G30.9, F01.50, F02.80	331	G30.9
275	AD	Mixed Alzheimer's and vascular dementia	331.0, 294.10, 290.40	G30.9, F01.50, F02.80	294.1	G30.9
276	AD	Mixed Alzheimer's and vascular dementia	331.0, 294.10, 290.40	G30.9, F01.50, F02.80	290.4	G30.9
277	AD	Mixed Alzheimer's and vascular dementia with behavior disturbances	331.0, 294.11, 290.40	G30.9, F01.518, F02.818	331	F02.818
278	AD	Mixed Alzheimer's and vascular dementia with behavior disturbances	331.0, 294.11, 290.40	G30.9, F01.518, F02.818	294.11	F02.818
279	AD	Mixed Alzheimer's and vascular dementia with behavior disturbances	331.0, 294.11, 290.40	G30.9, F01.518, F02.818	290.4	F02.818
280	AD	Mixed Alzheimer's and vascular dementia with behavior disturbances	331.0, 294.11, 290.40	G30.9, F01.518, F02.818	331	F01.518
281	AD	Mixed Alzheimer's and vascular dementia with behavior disturbances	331.0, 294.11, 290.40	G30.9, F01.518, F02.818	294.11	F01.518
282	AD	Mixed Alzheimer's and vascular dementia with behavior disturbances	331.0, 294.11, 290.40	G30.9, F01.518, F02.818	290.4	F01.518
283	AD	Mixed Alzheimer's and vascular dementia with behavior disturbances	331.0, 294.11, 290.40	G30.9, F01.518, F02.818	331	G30.9
284	AD	Mixed Alzheimer's and vascular dementia with behavior disturbances	331.0, 294.11, 290.40	G30.9, F01.518, F02.818	294.11	G30.9
285	AD	Mixed Alzheimer's and vascular dementia with behavior disturbances	331.0, 294.11, 290.40	G30.9, F01.518, F02.818	290.4	G30.9
286	AD	Moderate Alzheimer's dementia with mood disturbance, unspecified timing of dementia onset	331.0, 294.11	G30.9, F02.B3	331	F02.B3
287	AD	Moderate Alzheimer's dementia with mood disturbance, unspecified timing of dementia onset	331.0, 294.11	G30.9, F02.B3	294.11	F02.B3
288	AD	Moderate Alzheimer's dementia with mood disturbance, unspecified timing of dementia onset	331.0, 294.11	G30.9, F02.B3	331	G30.9
289	AD	Moderate Alzheimer's dementia with mood disturbance, unspecified timing of dementia onset	331.0, 294.11	G30.9, F02.B3	294.11	G30.9
290	AD	Moderate Alzheimer's dementia with other behavioral disturbance, unspecified timing of dementia onset	331.0, 294.11	G30.9, F02.B18	331	G30.9
291	AD	Moderate Alzheimer's dementia with other behavioral disturbance, unspecified timing of dementia onset	331.0, 294.11	G30.9, F02.B18	294.11	G30.9
292	AD	Moderate Alzheimer's dementia with other behavioral disturbance, unspecified timing of dementia onset	331.0, 294.11	G30.9, F02.B18	331	F02.B18
293	AD	Moderate Alzheimer's dementia with other behavioral disturbance, unspecified timing of dementia onset	331.0, 294.11	G30.9, F02.B18	294.11	F02.B18
294	AD	Moderate late onset Alzheimer's dementia with agitation	331.0, 294.11	G30.1, F02.B11	331	F02.B11

No.	Category	DiagnosisNM	CurrentICD9ListTXT	CurrentICD10ListTXT	ICD9CD	ICD10CD
295	AD	Moderate late onset Alzheimer's dementia with agitation	331.0, 294.11	G30.1, F02.B11	294.11	F02.B11
296	AD	Moderate late onset Alzheimer's dementia with agitation	331.0, 294.11	G30.1, F02.B11	331	G30.1
297	AD	Moderate late onset Alzheimer's dementia with agitation	331.0, 294.11	G30.1, F02.B11	294.11	G30.1
298	AD	Moderate late onset Alzheimer's dementia with anxiety	331.0, 294.11	G30.1, F02.B4	294.11	F02.B4
299	AD	Moderate late onset Alzheimer's dementia with anxiety	331.0, 294.11	G30.1, F02.B4	294.11	G30.1
300	AD	Moderate late onset Alzheimer's dementia with anxiety	331.0, 294.11	G30.1, F02.B4	331	G30.1
301	AD	Moderate late onset Alzheimer's dementia with anxiety	331.0, 294.11	G30.1, F02.B4	331	F02.B4
302	AD	Moderate late onset Alzheimer's dementia with other behavioral disturbance	331.0, 294.11	G30.1, F02.B18	294.11	G30.1
303	AD	Moderate late onset Alzheimer's dementia with other behavioral disturbance	331.0, 294.11	G30.1, F02.B18	294.11	F02.B18
304	AD	Moderate late onset Alzheimer's dementia with other behavioral disturbance	331.0, 294.11	G30.1, F02.B18	331	G30.1
305	AD	Moderate late onset Alzheimer's dementia with other behavioral disturbance	331.0, 294.11	G30.1, F02.B18	331	F02.B18
306	AD	Moderate late onset Alzheimer's dementia without behavioral disturbance, psychotic disturbance, mood disturbance, or anxiety	331.0, 294.10	G30.1, F02.B0	331	F02.B0
307	AD	Moderate late onset Alzheimer's dementia without behavioral disturbance, psychotic disturbance, mood disturbance, or anxiety	331.0, 294.10	G30.1, F02.B0	294.1	F02.B0
308	AD	Moderate late onset Alzheimer's dementia without behavioral disturbance, psychotic disturbance, mood disturbance, or anxiety	331.0, 294.10	G30.1, F02.B0	331	G30.1
309	AD	Moderate late onset Alzheimer's dementia without behavioral disturbance, psychotic disturbance, mood disturbance, or anxiety	331.0, 294.10	G30.1, F02.B0	294.1	G30.1
310	AD	Moderate late onset Alzheimer's dementia without behavioral disturbance, psychotic disturbance, mood disturbance, or anxiety	331.0, 294.10	G30.1, F02.B0	294.1	G30.1
311	AD	Moderate late onset Alzheimer's dementia without behavioral disturbance, psychotic disturbance, mood disturbance, or anxiety	331.0, 294.10	G30.1, F02.B0	331	F02.B0
312	AD	Moderate late onset Alzheimer's dementia without behavioral disturbance, psychotic disturbance, mood disturbance, or anxiety	331.0, 294.10	G30.1, F02.B0	294.1	F02.B0
313	AD	Moderate late onset Alzheimer's dementia without behavioral disturbance, psychotic disturbance, mood disturbance, or anxiety	331.0, 294.10	G30.1, F02.B0	331	G30.1
314	AD	Moderate major neurocognitive disorder due to Alzheimer's disease without behavioral disturbance	331.0, 294.9	G30.9, F02.B0	331	F02.B0
315	AD	Moderate major neurocognitive disorder due to Alzheimer's disease without behavioral disturbance	331.0, 294.9	G30.9, F02.B0	294.9	F02.B0
316	AD	Moderate major neurocognitive disorder due to Alzheimer's disease without behavioral disturbance	331.0, 294.9	G30.9, F02.B0	331	G30.9
317	AD	Moderate major neurocognitive disorder due to Alzheimer's disease without behavioral disturbance	331.0, 294.9	G30.9, F02.B0	294.9	G30.9
318	AD	Moderate major neurocognitive disorder due to probable Alzheimer's disease, with behavioral disturbance	294.2	F03.91	294.2	F03.91
319	AD	Moderate possible major neurocognitive disorder due to Alzheimer's disease	IMO0001	IMO0001	IMO0001	IMO0001
320	AD	Moderate probable major neurocognitive disorder due to Alzheimer's disease with behavioral disturbance	294.2	F03.91	294.2	F03.91
321	AD	Moderate probable major neurocognitive disorder due to Alzheimer's disease without behavioral disturbance	294.2	F03.90	294.2	F03.90
322	AD	Other Alzheimer's disease	331	G30.8, F02.80	331	F02.80
323	AD	Other Alzheimer's disease	331	G30.8, F02.80	331	G30.8
324	AD	Other Alzheimer's disease	NULL	G30.8	NULL	G30.8
325	AD	Possible major neurocognitive disorder due to Alzheimer's disease	294.2	F03.90	294.2	F03.90
326	AD	Primary degenerative dementia of Alzheimer type	331.0, 294.10	G30.9, F02.80	331	F02.80
327	AD	Primary degenerative dementia of Alzheimer type	331.0, 294.10	G30.9, F02.80	294.1	F02.80
328	AD	Primary degenerative dementia of Alzheimer type	331.0, 294.10	G30.9, F02.80	331	G30.9
329	AD	Primary degenerative dementia of Alzheimer type	331.0, 294.10	G30.9, F02.80	294.1	G30.9
330	AD	Primary degenerative dementia of the Alzheimer type, presenile onset	331	G30.0, F02.80	331	F02.80
331	AD	Primary degenerative dementia of the Alzheimer type, presenile onset	331	G30.0, F02.80	331	G30.0
332	AD	Primary degenerative dementia of the Alzheimer type, presenile onset, with delusions	331.0, 294.11, 297.9	G30.0, F02.82	331	F02.82
333	AD	Primary degenerative dementia of the Alzheimer type, presenile onset, with delusions	331.0, 294.11, 297.9	G30.0, F02.82	294.11	F02.82
334	AD	Primary degenerative dementia of the Alzheimer type, presenile onset, with delusions	331.0, 294.11, 297.9	G30.0, F02.82	297.9	F02.82
335	AD	Primary degenerative dementia of the Alzheimer type, presenile onset, with delusions	331.0, 294.11, 297.9	G30.0, F02.82	331	G30.0
336	AD	Primary degenerative dementia of the Alzheimer type, presenile onset, with delusions	331.0, 294.11, 297.9	G30.0, F02.82	294.11	G30.0

No.	Category	DiagnosisNM	CurrentICD9ListTXT	CurrentICD10ListTXT	ICD9CD	ICD10CD
337	AD	Primary degenerative dementia of the Alzheimer type, presenile onset, with delusions	331.0, 294.11, 297.9	G30.0, F02.82	297.9	G30.0
338	AD	Primary degenerative dementia of the Alzheimer type, presenile onset, with depression	331.0, 290.13	G30.0, F02.83	331	F02.83
339	AD	Primary degenerative dementia of the Alzheimer type, presenile onset, with depression	331.0, 290.13	G30.0, F02.83	290.13	F02.83
340	AD	Primary degenerative dementia of the Alzheimer type, presenile onset, with depression	331.0, 290.13	G30.0, F02.83	331	G30.0
341	AD	Primary degenerative dementia of the Alzheimer type, presenile onset, with depression	331.0, 290.13	G30.0, F02.83	290.13	G30.0
342	AD	Primary degenerative dementia of the Alzheimer type, senile onset, uncomplicated	331.0, 294.10	G30.1, F02.80	331	F02.80
343	AD	Primary degenerative dementia of the Alzheimer type, senile onset, uncomplicated	331.0, 294.10	G30.1, F02.80	294.1	F02.80
344	AD	Primary degenerative dementia of the Alzheimer type, senile onset, uncomplicated	331.0, 294.10	G30.1, F02.80	331	G30.1
345	AD	Primary degenerative dementia of the Alzheimer type, senile onset, uncomplicated	331.0, 294.10	G30.1, F02.80	294.1	G30.1
346	AD	Primary degenerative dementia of the Alzheimer type, senile onset, with delirium	331.0, 294.11	G30.1, F02.82	331	F02.82
347	AD	Primary degenerative dementia of the Alzheimer type, senile onset, with delirium	331.0, 294.11	G30.1, F02.82	294.11	F02.82
348	AD	Primary degenerative dementia of the Alzheimer type, senile onset, with delirium	331.0, 294.11	G30.1, F02.82	331	G30.1
349	AD	Primary degenerative dementia of the Alzheimer type, senile onset, with delirium	331.0, 294.11	G30.1, F02.82	294.11	G30.1
350	AD	Primary degenerative dementia of the Alzheimer type, senile onset, with delusions	331.0, 290.20	G30.1, F02.818	331	F02.818
351	AD	Primary degenerative dementia of the Alzheimer type, senile onset, with delusions	331.0, 290.20	G30.1, F02.818	290.2	F02.818
352	AD	Primary degenerative dementia of the Alzheimer type, senile onset, with delusions	331.0, 290.20	G30.1, F02.818	331	G30.1
353	AD	Primary degenerative dementia of the Alzheimer type, senile onset, with delusions	331.0, 290.20	G30.1, F02.818	290.2	G30.1
354	AD	Probable major neurocognitive disorder due to Alzheimer's disease with behavioral disturbance	294.2	F03.91	294.2	F03.91
355	AD	Probable major neurocognitive disorder due to Alzheimer's disease without behavioral disturbance	294.2	F03.90	294.2	F03.90
356	AD	Senile dementia of Alzheimer's type	331.0, 294.10	G30.1, F02.80	331	F02.80
357	AD	Senile dementia of Alzheimer's type	331.0, 294.10	G30.1, F02.80	294.1	F02.80
358	AD	Senile dementia of Alzheimer's type	331.0, 294.10	G30.1, F02.80	331	G30.1
359	AD	Senile dementia of Alzheimer's type	331.0, 294.10	G30.1, F02.80	294.1	G30.1
360	AD	Severe Alzheimer's dementia, unspecified timing of dementia onset, unspecified whether behavioral, psychotic, or mood disturbance or anxiety	331.0, 294.10	G30.9, F02.C0	331	G30.9
361	AD	Severe Alzheimer's dementia, unspecified timing of dementia onset, unspecified whether behavioral, psychotic, or mood disturbance or anxiety	331.0, 294.10	G30.9, F02.C0	294.1	G30.9
362	AD	Severe Alzheimer's dementia, unspecified timing of dementia onset, unspecified whether behavioral, psychotic, or mood disturbance or anxiety	331.0, 294.10	G30.9, F02.C0	331	F02.C0
363	AD	Severe Alzheimer's dementia, unspecified timing of dementia onset, unspecified whether behavioral, psychotic, or mood disturbance or anxiety	331.0, 294.10	G30.9, F02.C0	294.1	F02.C0
364	AD	Severe major neurocognitive disorder due to Alzheimer's disease with behavioral disturbance	331.0, 294.11	G30.9, F02.C18	331	F02.C18
365	AD	Severe major neurocognitive disorder due to Alzheimer's disease with behavioral disturbance	331.0, 294.11	G30.9, F02.C18	294.11	F02.C18
366	AD	Severe major neurocognitive disorder due to Alzheimer's disease with behavioral disturbance	331.0, 294.11	G30.9, F02.C18	331	G30.9
367	AD	Severe major neurocognitive disorder due to Alzheimer's disease with behavioral disturbance	331.0, 294.11	G30.9, F02.C18	294.11	G30.9
368	AD	Sporadic Alzheimer's disease	331	G30.9, F02.80	331	F02.80
369	AD	Sporadic Alzheimer's disease	331	G30.9, F02.80	331	G30.9
370	AD	Uncomplicated late onset Alzheimer's dementia	331	G30.1, F02.80	331	F02.80
371	AD	Uncomplicated late onset Alzheimer's dementia	331	G30.1, F02.80	331	G30.1
372	Dementia Unspecified	Advanced dementia	294.2	F03.C0	294.2	F03.C0
373	Dementia Unspecified	Age-related cognitive decline	294.9	R41.81	294.9	R41.81
374	Dementia Unspecified	Age-related memory disorder	780.93	R41.3	780.93	R41.3
375	Dementia Unspecified	Agitation due to dementia	294.21	F03.911	294.21	F03.911
376	Dementia Unspecified	Amnesia	780.93	R41.3	780.93	R41.3
377	Dementia Unspecified	Amnesia memory loss	780.93	R41.3	780.93	R41.3
378	Dementia Unspecified	Amnestic disorder	294.8	R41.3	294.8	R41.3

No.	Category	DiagnosisNM	CurrentICD9ListTXT	CurrentICD10ListTXT	ICD9CD	ICD10CD
379	Dementia Unspecified	Cognitive and behavioral changes	799.59, 312.9	R41.89, R46.89	312.9	R41.89
380	Dementia Unspecified	Cognitive and behavioral changes	799.59, 312.9	R41.89, R46.89	312.9	R46.89
381	Dementia Unspecified	Cognitive and behavioral changes	799.59, 312.9	R41.89, R46.89	799.59	R41.89
382	Dementia Unspecified	Cognitive and behavioral changes	799.59, 312.9	R41.89, R46.89	799.59	R46.89
383	Dementia Unspecified	Cognitive and neurobehavioral dysfunction following brain injury, sequela	907	S06.9X0S, G31.89	907	G31.89
384	Dementia Unspecified	Cognitive and neurobehavioral dysfunction following brain injury, sequela	907	S06.9X0S, G31.89	907	S06.9X0S
385	Dementia Unspecified	Cognitive change	799.59	R41.89	799.59	R41.89
386	Dementia Unspecified	Cognitive changes	799.59	R41.89	799.59	R41.89
387	Dementia Unspecified	Cognitive communication deficit	799.52	R41.841	799.52	R41.841
388	Dementia Unspecified	Cognitive communication disorder	315.32	R41.841	315.32	R41.841
389	Dementia Unspecified	Cognitive complaints	799.59	R41.9	799.59	R41.9
390	Dementia Unspecified	Cognitive complaints with normal exam	799.59	R41.9	799.59	R41.9
391	Dementia Unspecified	Cognitive decline	294.9	R41.89	294.9	R41.89
392	Dementia Unspecified	Cognitive deficits	294.9	R41.89	294.9	R41.89
393	Dementia Unspecified	Cognitive disorder	294.9	F09	294.9	F09
394	Dementia Unspecified	Cognitive dysfunction	294.9	F09	294.9	F09
395	Dementia Unspecified	Cognitive dysfunction associated with depression	294.9, 311	F09, F32.A	294.9	F32.A
396	Dementia Unspecified	Cognitive dysfunction associated with depression	294.9, 311	F09, F32.A	311	F09
397	Dementia Unspecified	Cognitive dysfunction associated with depression	294.9, 311	F09, F32.A	294.9	F09
398	Dementia Unspecified	Cognitive dysfunction associated with depression	294.9, 311	F09, F32.A	311	F32.A
399	Dementia Unspecified	Cognitive impairment	294.9	R41.89	294.9	R41.89
400	Dementia Unspecified	Complaints of memory disturbance	780.93	R41.3	780.93	R41.3
401	Dementia Unspecified	Creutzfeldt Jakob disease	46.19	A81.00	46.19	A81.00
402	Dementia Unspecified	Dementia	294.2	F03.90	294.2	F03.90
403	Dementia Unspecified	Dementia arising in the senium and presenium	290.0, 290.10	F03.90	290	F03.90
404	Dementia Unspecified	Dementia arising in the senium and presenium	290.0, 290.10	F03.90	290.1	F03.90
405	Dementia Unspecified	Dementia associated with Jakob-Creutzfeldt disease	046.19, 294.10	A81.00, F02.80	46.19	F02.80
406	Dementia Unspecified	Dementia associated with Jakob-Creutzfeldt disease	046.19, 294.10	A81.00, F02.80	294.1	A81.00
407	Dementia Unspecified	Dementia associated with Jakob-Creutzfeldt disease	046.19, 294.10	A81.00, F02.80	46.19	A81.00
408	Dementia Unspecified	Dementia associated with Jakob-Creutzfeldt disease	046.19, 294.10	A81.00, F02.80	294.1	F02.80
409	Dementia Unspecified	Dementia associated with other underlying disease with behavioral disturbance	294.8, 294.11	F02.818	294.11	F02.818
410	Dementia Unspecified	Dementia associated with other underlying disease with behavioral disturbance	294.8, 294.11	F02.818	294.8	F02.818
411	Dementia Unspecified	Dementia associated with other underlying disease without behavioral disturbance	294.1	F02.80	294.1	F02.80
412	Dementia Unspecified	Dementia due to another medical condition	294.1	F02.80	294.1	F02.80
413	Dementia Unspecified	Dementia due to general medical condition, without behavioral disturbance	294.1	F02.80	294.1	F02.80
414	Dementia Unspecified	Dementia due to HIV infection without behavioral disturbance	042, 294.10	B20, F02.80	294.1	B20
415	Dementia Unspecified	Dementia due to HIV infection without behavioral disturbance	042, 294.10	B20, F02.80	294.1	F02.80
416	Dementia Unspecified	Dementia due to HIV infection without behavioral disturbance	042, 294.10	B20, F02.80	042	F02.80
417	Dementia Unspecified	Dementia due to HIV infection without behavioral disturbance	042, 294.10	B20, F02.80	042	B20
418	Dementia Unspecified	Dementia due to medical condition	294.1	F02.80	294.1	F02.80
419	Dementia Unspecified	Dementia in conditions classified elsewhere with behavioral disturbance	294.11	F02.818	294.11	F02.818
420	Dementia Unspecified	Dementia with behavioral disturbance	294.21	F03.918	294.21	F03.918

No.	Category	DiagnosisNM	CurrentICD9ListTXT	CurrentICD10ListTXT	ICD9CD	ICD10CD
421	Dementia Unspecified	Dementia with behavioral disturbance, unspecified dementia type	294.21	F03.91	294.21	F03.91
422	Dementia Unspecified	Dementia without behavioral disturbance, unspecified dementia type	294.2	F03.90	294.2	F03.90
423	Dementia Unspecified	Dementia, primary degenerative, senile	290	F03.90	290	F03.90
424	Dementia Unspecified	Dementia, with behavioral disturbance	294.21	F03.91	294.21	F03.91
425	Dementia Unspecified	Dementia, without behavioral disturbance	294.2	F03.90	294.2	F03.90
426	Dementia Unspecified	Disinhibited behavior due to dementia	294.21	F03.918	294.21	F03.918
427	Dementia Unspecified	Familial dementia	294.8	F03.90	294.8	F03.90
428	Dementia Unspecified	Frontal lobe syndrome	310	F07.0	310	F07.0
429	Dementia Unspecified	History of short term memory loss	V12.49	Z87.898	V12.49	Z87.898
430	Dementia Unspecified	Impaired cognition	294.9	R41.89	294.9	R41.89
431	Dementia Unspecified	Major neurocognitive disorder	294.2	F03.90	294.2	F03.90
432	Dementia Unspecified	Memory change	780.93	R41.3	780.93	R41.3
433	Dementia Unspecified	Memory changes	780.93	R41.3	780.93	R41.3
434	Dementia Unspecified	Memory deficit	780.93	R41.3	780.93	R41.3
435	Dementia Unspecified	Memory deficits	780.93	R41.3	780.93	R41.3
436	Dementia Unspecified	Memory disorder	780.93	R41.3	780.93	R41.3
437	Dementia Unspecified	Memory disturbance	780.93	R41.3	780.93	R41.3
438	Dementia Unspecified	Memory impairment	780.93	R41.3	780.93	R41.3
439	Dementia Unspecified	Memory impairment of gradual onset	780.93	R41.3	780.93	R41.3
440	Dementia Unspecified	Memory loss	780.93	R41.3	780.93	R41.3
441	Dementia Unspecified	Memory loss due to medical condition	780.93	R41.3	780.93	R41.3
442	Dementia Unspecified	Memory loss of unknown cause	780.93	R41.3	780.93	R41.3
443	Dementia Unspecified	Mild dementia	294.2	F03.A0	294.2	F03.A0
444	Dementia Unspecified	Mild dementia without behavioral disturbance, psychotic disturbance, mood disturbance, or anxiety, unspecified dementia type	294.1	F03.A0	294.1	F03.A0
445	Dementia Unspecified	Mixed dementia	331.0, 294.10, 290.40	G30.9, F01.50, F02.80	294.1	G30.9
446	Dementia Unspecified	Mixed dementia	331.0, 294.10, 290.40	G30.9, F01.50, F02.80	290.4	F01.50
447	Dementia Unspecified	Mixed dementia	331.0, 294.10, 290.40	G30.9, F01.50, F02.80	290.4	F02.80
448	Dementia Unspecified	Mixed dementia	331.0, 294.10, 290.40	G30.9, F01.50, F02.80	294.1	F01.50
449	Dementia Unspecified	Mixed dementia	331.0, 294.10, 290.40	G30.9, F01.50, F02.80	294.1	F02.80
450	Dementia Unspecified	Mixed dementia	331.0, 294.10, 290.40	G30.9, F01.50, F02.80	331	G30.9
451	Dementia Unspecified	Mixed dementia	331.0, 294.10, 290.40	G30.9, F01.50, F02.80	290.4	G30.9
452	Dementia Unspecified	Mixed dementia	331.0, 294.10, 290.40	G30.9, F01.50, F02.80	331	F01.50
453	Dementia Unspecified	Mixed dementia	331.0, 294.10, 290.40	G30.9, F01.50, F02.80	331	F02.80
454	Dementia Unspecified	Moderate dementia	294.2	F03.B0	294.2	F03.B0
455	Dementia Unspecified	Moderate dementia with behavioral disturbance	294.21	F03.B18	294.21	F03.B18
456	Dementia Unspecified	Moderate dementia with other behavioral disturbance, unspecified dementia type	294.21	F03.B18	294.21	F03.B18
457	Dementia Unspecified	Moderate dementia without behavioral disturbance, psychotic disturbance, mood disturbance, or anxiety, unspecified dementia type	294.2	F03.B0	294.2	F03.B0
458	Dementia Unspecified	Multi-infarct dementia, unspecified dementia severity, unspecified whether behavioral, psychotic, or mood disturbance or anxiety	290.4	F01.50	290.4	F01.50
459	Dementia Unspecified	Multifactorial cognitive dysfunction	294.9	F09	294.9	F09
460	Dementia Unspecified	Neurodegenerative cognitive impairment	331.9	G31.9	331.9	G31.9
461	Dementia Unspecified	Neurodegenerative dementia with behavioral disturbance	294.21	F03.918	294.21	F03.918
462	Dementia Unspecified	Neurodegenerative dementia without behavioral disturbance	294.2	F03.90	294.2	F03.90

No.	Category	DiagnosisNM	CurrentICD9ListTXT	CurrentICD10ListTXT	ICD9CD	ICD10CD
463	Dementia Unspecified	Neurodegenerative dementia, with behavioral disturbance	294.21	F03.918	294.21	F03.918
464	Dementia Unspecified	Presenile dementia	290.1	F03.90	290.1	F03.90
465	Dementia Unspecified	Presenile dementia without behavioral disturbance	290.1	F03.90	290.1	F03.90
466	Dementia Unspecified	Primary degenerative dementia	290.9	F03.90	290.9	F03.90
467	Dementia Unspecified	Prion disease	46.79	A81.9	46.79	A81.9
468	Dementia Unspecified	Rapidly progressive dementia	294.2	F03.90	294.2	F03.90
469	Dementia Unspecified	Semantic dementia	331.19, 294.10	G31.09, F02.80	331.19	F02.80
470	Dementia Unspecified	Semantic dementia	331.19, 294.10	G31.09, F02.80	294.1	F02.80
471	Dementia Unspecified	Semantic dementia	331.19, 294.10	G31.09, F02.80	331.19	G31.09
472	Dementia Unspecified	Semantic dementia	331.19, 294.10	G31.09, F02.80	294.1	G31.09
473	Dementia Unspecified	Semantic dementia with behavioral disturbance	331.19, 294.11	G31.09, F02.818	331.19	G31.09
474	Dementia Unspecified	Semantic dementia with behavioral disturbance	331.19, 294.11	G31.09, F02.818	294.11	G31.09
475	Dementia Unspecified	Semantic dementia with behavioral disturbance	331.19, 294.11	G31.09, F02.818	331.19	F02.818
476	Dementia Unspecified	Semantic dementia with behavioral disturbance	331.19, 294.11	G31.09, F02.818	294.11	F02.818
477	Dementia Unspecified	Semantic dementia without behavioral disturbance	331.19, 294.10	G31.09, F02.80	294.1	F02.80
478	Dementia Unspecified	Semantic dementia without behavioral disturbance	331.19, 294.10	G31.09, F02.80	331.19	G31.09
479	Dementia Unspecified	Semantic dementia without behavioral disturbance	331.19, 294.10	G31.09, F02.80	294.1	G31.09
480	Dementia Unspecified	Semantic dementia without behavioral disturbance	331.19, 294.10	G31.09, F02.80	331.19	F02.80
481	Dementia Unspecified	Semantic dementia, with behavioral disturbance	331.19, 294.11	F02.81	331.19	F02.81
482	Dementia Unspecified	Semantic dementia, with behavioral disturbance	331.19, 294.11	F02.81	294.11	F02.81
483	Dementia Unspecified	Semantic memory disorder	780.93	R41.89	780.93	R41.89
484	Dementia Unspecified	Senile dementia with delusional features with behavioral disturbance	290.2	F03.92, F03.918	290.2	F03.92
485	Dementia Unspecified	Senile dementia with delusional features with behavioral disturbance	290.2	F03.92, F03.918	290.2	F03.918
486	Dementia Unspecified	Senile dementia, uncomplicated	290	F03.90	290	F03.90
487	Dementia Unspecified	Severe dementia associated with other underlying disease, with other behavioral disturbance	294.11	F02.C18	294.11	F02.C18
488	Dementia Unspecified	Subcortical dementia	294.2	F03.90	294.2	F03.90
489	Dementia Unspecified	Transient global amnesia	437.7	G45.4	437.7	G45.4
490	Dementia Unspecified	Uncomplicated presenile dementia	290.1	F03.90	290.1	F03.90
491	FTD	Amyotrophic lateral sclerosis with frontotemporal dementia	335.20, 331.19, 294.10	G12.21, G31.09, F02.80	335.2	F02.80
492	FTD	Amyotrophic lateral sclerosis with frontotemporal dementia	335.20, 331.19, 294.10	G12.21, G31.09, F02.80	331.19	F02.80
493	FTD	Amyotrophic lateral sclerosis with frontotemporal dementia	335.20, 331.19, 294.10	G12.21, G31.09, F02.80	294.1	F02.80
494	FTD	Amyotrophic lateral sclerosis with frontotemporal dementia	335.20, 331.19, 294.10	G12.21, G31.09, F02.80	335.2	G31.09
495	FTD	Amyotrophic lateral sclerosis with frontotemporal dementia	335.20, 331.19, 294.10	G12.21, G31.09, F02.80	331.19	G31.09
496	FTD	Amyotrophic lateral sclerosis with frontotemporal dementia	335.20, 331.19, 294.10	G12.21, G31.09, F02.80	294.1	G31.09
497	FTD	Amyotrophic lateral sclerosis with frontotemporal dementia	335.20, 331.19, 294.10	G12.21, G31.09, F02.80	335.2	G12.21
498	FTD	Amyotrophic lateral sclerosis with frontotemporal dementia	335.20, 331.19, 294.10	G12.21, G31.09, F02.80	331.19	G12.21
499	FTD	Amyotrophic lateral sclerosis with frontotemporal dementia	335.20, 331.19, 294.10	G12.21, G31.09, F02.80	294.1	G12.21
500	FTD	Behavioral variant frontotemporal dementia	331.19	G31.09, F02.818	331.19	F02.818
501	FTD	Behavioral variant frontotemporal dementia	331.19	G31.09, F02.818	331.19	G31.09
502	FTD	C9ORF72-related amyotrophic lateral sclerosis and frontotemporal dementia	335.20, 331.19, 294.10	G12.21, G31.09, F02.80	335.2	G12.21
503	FTD	C9ORF72-related amyotrophic lateral sclerosis and frontotemporal dementia	335.20, 331.19, 294.10	G12.21, G31.09, F02.80	331.19	G12.21
504	FTD	C9ORF72-related amyotrophic lateral sclerosis and frontotemporal dementia	335.20, 331.19, 294.10	G12.21, G31.09, F02.80	294.1	G12.21

No.	Category	DiagnosisNM	CurrentICD9ListTXT	CurrentICD10ListTXT	ICD9CD	ICD10CD
505	FTD	C9ORF72-related amyotrophic lateral sclerosis and frontotemporal dementia	335.20, 331.19, 294.10	G12.21, G31.09, F02.80	335.2	G31.09
506	FTD	C9ORF72-related amyotrophic lateral sclerosis and frontotemporal dementia	335.20, 331.19, 294.10	G12.21, G31.09, F02.80	331.19	G31.09
507	FTD	C9ORF72-related amyotrophic lateral sclerosis and frontotemporal dementia	335.20, 331.19, 294.10	G12.21, G31.09, F02.80	294.1	G31.09
508	FTD	C9ORF72-related amyotrophic lateral sclerosis and frontotemporal dementia	335.20, 331.19, 294.10	G12.21, G31.09, F02.80	335.2	F02.80
509	FTD	C9ORF72-related amyotrophic lateral sclerosis and frontotemporal dementia	335.20, 331.19, 294.10	G12.21, G31.09, F02.80	331.19	F02.80
510	FTD	C9ORF72-related amyotrophic lateral sclerosis and frontotemporal dementia	335.20, 331.19, 294.10	G12.21, G31.09, F02.80	294.1	F02.80
511	FTD	Dementia due to Pick's disease with behavioral disturbance	331.11, 294.11	G31.01, F02.818	331.11	F02.818
512	FTD	Dementia due to Pick's disease with behavioral disturbance	331.11, 294.11	G31.01, F02.818	294.11	F02.818
513	FTD	Dementia due to Pick's disease with behavioral disturbance	331.11, 294.11	G31.01, F02.818	331.11	G31.01
514	FTD	Dementia due to Pick's disease with behavioral disturbance	331.11, 294.11	G31.01, F02.818	294.11	G31.01
515	FTD	Dementia due to Pick's disease without behavioral disturbance	331.11, 294.10	G31.01, F02.80	331.11	F02.80
516	FTD	Dementia due to Pick's disease without behavioral disturbance	331.11, 294.10	G31.01, F02.80	294.1	F02.80
517	FTD	Dementia due to Pick's disease without behavioral disturbance	331.11, 294.10	G31.01, F02.80	331.11	G31.01
518	FTD	Dementia due to Pick's disease without behavioral disturbance	331.11, 294.10	G31.01, F02.80	294.1	G31.01
519	FTD	Dementia of frontal lobe type	331.19	G31.09, F02.80	331.19	F02.80
520	FTD	Dementia of frontal lobe type	331.19	G31.09, F02.80	331.19	G31.09
521	FTD	Dementia, frontotemporal	331.19	G31.09, F02.80	331.19	F02.80
522	FTD	Dementia, frontotemporal	331.19	G31.09, F02.80	331.19	G31.09
523	FTD	Frontal dementia	331.19	G31.09, F02.80	331.19	F02.80
524	FTD	Frontal dementia	331.19	G31.09, F02.80	331.19	G31.09
525	FTD	Frontal lobe and executive function deficit	NULL	R41.844	NULL	R41.844
526	FTD	Frontal lobe and executive function deficit	799.55	R41.844	799.55	R41.844
527	FTD	Frontal lobe dementia	331.19	G31.09, F02.80	331.19	F02.80
528	FTD	Frontal lobe dementia	331.19	G31.09, F02.80	331.19	G31.09
529	FTD	Fronto-temporal dementia	331.19	G31.09, F02.80	331.19	F02.80
530	FTD	Fronto-temporal dementia	331.19	G31.09, F02.80	331.19	G31.09
531	FTD	Frontotemporal brain disease	331.19	G31.09, F02.80	331.19	F02.80
532	FTD	Frontotemporal brain disease	331.19	G31.09, F02.80	331.19	G31.09
533	FTD	Frontotemporal dementia	331.19	G31.09, F02.80	331.19	F02.80
534	FTD	Frontotemporal dementia	331.19	G31.09, F02.80	331.19	G31.09
535	FTD	Frontotemporal dementia	NULL	G31.0	NULL	G31.0
536	FTD	Frontotemporal dementia	331.1	NULL	331.1	NULL
537	FTD	Frontotemporal dementia associated with mutation in MAPT gene	331.19	G31.09, F02.80	331.19	F02.80
538	FTD	Frontotemporal dementia associated with mutation in MAPT gene	331.19	G31.09, F02.80	331.19	G31.09
539	FTD	Frontotemporal dementia associated with mutation in PGRN gene	331.19	G31.09, F02.80	331.19	F02.80
540	FTD	Frontotemporal dementia associated with mutation in PGRN gene	331.19	G31.09, F02.80	331.19	G31.09
541	FTD	Frontotemporal dementia with behavioral disturbance	331.19, 294.11	G31.09, F02.818	331.19	F02.818
542	FTD	Frontotemporal dementia with behavioral disturbance	331.19, 294.11	G31.09, F02.818	294.11	F02.818
543	FTD	Frontotemporal dementia with behavioral disturbance	331.19, 294.11	G31.09, F02.818	331.19	G31.09
544	FTD	Frontotemporal dementia with behavioral disturbance	331.19, 294.11	G31.09, F02.818	294.11	G31.09
545	FTD	Frontotemporal dementia with motor neuron disease	335.20, 331.19	G31.09, F02.80, G12.20	335.2	G12.20
546	FTD	Frontotemporal dementia with motor neuron disease	335.20, 331.19	G31.09, F02.80, G12.20	331.19	G12.20

No.	Category	DiagnosisNM	CurrentICD9ListTXT	CurrentICD10ListTXT	ICD9CD	ICD10CD
547	FTD	Frontotemporal dementia with motor neuron disease	335.20, 331.19	G31.09, F02.80, G12.20	335.2	F02.80
548	FTD	Frontotemporal dementia with motor neuron disease	335.20, 331.19	G31.09, F02.80, G12.20	331.19	F02.80
549	FTD	Frontotemporal dementia with motor neuron disease	335.20, 331.19	G31.09, F02.80, G12.20	335.2	G31.09
550	FTD	Frontotemporal dementia with motor neuron disease	335.20, 331.19	G31.09, F02.80, G12.20	331.19	G31.09
551	FTD	Frontotemporal dementia without behavioral disturbance	331.19, 294.10	G31.09, F02.80	331.19	F02.80
552	FTD	Frontotemporal dementia without behavioral disturbance	331.19, 294.10	G31.09, F02.80	294.1	F02.80
553	FTD	Frontotemporal dementia without behavioral disturbance	331.19, 294.10	G31.09, F02.80	331.19	G31.09
554	FTD	Frontotemporal dementia without behavioral disturbance	331.19, 294.10	G31.09, F02.80	294.1	G31.09
555	FTD	Frontotemporal lobar degeneration	331.19, 294.10	G31.09, F02.80	331.19	F02.80
556	FTD	Frontotemporal lobar degeneration	331.19, 294.10	G31.09, F02.80	294.1	F02.80
557	FTD	Frontotemporal lobar degeneration	331.19, 294.10	G31.09, F02.80	331.19	G31.09
558	FTD	Frontotemporal lobar degeneration	331.19, 294.10	G31.09, F02.80	294.1	G31.09
559	FTD	Frontotemporal lobar degeneration	331.19, 294.10	G31.09, F02.80	331.19	F02.80
560	FTD	Frontotemporal lobar degeneration	331.19, 294.10	G31.09, F02.80	294.1	G31.09
561	FTD	Frontotemporal lobar degeneration	331.19, 294.10	G31.09, F02.80	294.1	F02.80
562	FTD	Frontotemporal lobar degeneration	331.19, 294.10	G31.09, F02.80	331.19	G31.09
563	FTD	FTD with MND (frontotemporal dementia with motor neuron disease)	335.20, 331.19	G31.09, F02.80, G12.20	335.2	G12.20
564	FTD	FTD with MND (frontotemporal dementia with motor neuron disease)	335.20, 331.19	G31.09, F02.80, G12.20	331.19	G12.20
565	FTD	FTD with MND (frontotemporal dementia with motor neuron disease)	335.20, 331.19	G31.09, F02.80, G12.20	335.2	F02.80
566	FTD	FTD with MND (frontotemporal dementia with motor neuron disease)	335.20, 331.19	G31.09, F02.80, G12.20	331.19	F02.80
567	FTD	FTD with MND (frontotemporal dementia with motor neuron disease)	335.20, 331.19	G31.09, F02.80, G12.20	335.2	G31.09
568	FTD	FTD with MND (frontotemporal dementia with motor neuron disease)	335.20, 331.19	G31.09, F02.80, G12.20	331.19	G31.09
569	FTD	GRN-related frontotemporal dementia	331.19	G31.09, F02.80	331.19	F02.80
570	FTD	GRN-related frontotemporal dementia	331.19	G31.09, F02.80	331.19	G31.09
571	FTD	Left temporal atrophy variant of frontotemporal dementia	331.19, 294.10	G31.09, F02.80	331.19	F02.80
572	FTD	Left temporal atrophy variant of frontotemporal dementia	331.19, 294.10	G31.09, F02.80	294.1	F02.80
573	FTD	Left temporal atrophy variant of frontotemporal dementia	331.19, 294.10	G31.09, F02.80	331.19	G31.09
574	FTD	Left temporal atrophy variant of frontotemporal dementia	331.19, 294.10	G31.09, F02.80	294.1	G31.09
575	FTD	Major frontotemporal neurocognitive disorder, possible	IMO0001	IMO0001	IMO0001	IMO0001
576	FTD	Major frontotemporal neurocognitive disorder, probable, with behavioral disturbance	IMO0001	IMO0001	IMO0001	IMO0001
577	FTD	Major neurocognitive disorder due to possible frontotemporal lobar degeneration, with behavioral disturbance	294.2	F03.91	294.2	F03.91
578	FTD	Major neurocognitive disorder due to probable frontotemporal lobar degeneration, with behavioral disturbance	294.2	F03.91	294.2	F03.91
579	FTD	MAPT-related frontotemporal dementia	331.19	G31.09, F02.80	331.19	F02.80
580	FTD	MAPT-related frontotemporal dementia	331.19	G31.09, F02.80	331.19	G31.09
581	FTD	Mild frontotemporal neurocognitive disorder	331.83	G31.09, F02.A0	331.83	F02.A0
582	FTD	Mild frontotemporal neurocognitive disorder	331.83	G31.09, F02.A0	331.83	G31.09
583	FTD	Mild major neurocognitive disorder due to frontotemporal lobar degeneration with behavioral disturbance	331.9, 294.9	G31.09, F02.A18	331.9	F02.A18
584	FTD	Mild major neurocognitive disorder due to frontotemporal lobar degeneration with behavioral disturbance	331.9, 294.9	G31.09, F02.A18	294.9	F02.A18
585	FTD	Mild major neurocognitive disorder due to frontotemporal lobar degeneration with behavioral disturbance	331.9, 294.9	G31.09, F02.A18	331.9	G31.09
586	FTD	Mild major neurocognitive disorder due to frontotemporal lobar degeneration with behavioral disturbance	331.9, 294.9	G31.09, F02.A18	294.9	G31.09
587	FTD	Mild major neurocognitive disorder due to frontotemporal lobar degeneration without behavioral disturbance	331.9, 294.9	G31.09, F02.A0	331.9	F02.A0
588	FTD	Mild major neurocognitive disorder due to frontotemporal lobar degeneration without behavioral disturbance	331.9, 294.9	G31.09, F02.A0	294.9	F02.A0

No.	Category	DiagnosisNM	CurrentICD9ListTXT	CurrentICD10ListTXT	ICD9CD	ICD10CD
589	FTD	Mild major neurocognitive disorder due to frontotemporal lobar degeneration without behavioral disturbance	331.9, 294.9	G31.09, F02.A0	331.9	G31.09
590	FTD	Mild major neurocognitive disorder due to frontotemporal lobar degeneration without behavioral disturbance	331.9, 294.9	G31.09, F02.A0	294.9	G31.09
591	FTD	Mild possible major frontotemporal neurocognitive disorder	IMO0001	IMO0001	IMO0001	IMO0001
592	FTD	Mild probable major frontotemporal neurocognitive disorder with behavioral disturbance	IMO0001	IMO0001	IMO0001	IMO0001
593	FTD	Moderate possible major frontotemporal neurocognitive disorder	IMO0001	IMO0001	IMO0001	IMO0001
594	FTD	Other frontotemporal dementia	331.19	G31.09, F02.80	331.19	F02.80
595	FTD	Other frontotemporal dementia	331.19	G31.09, F02.80	331.19	G31.09
596	FTD	Other frontotemporal dementia	331.19	G31.09, F02.80	331.19	F02.80
597	FTD	Other frontotemporal dementia	331.19	G31.09, F02.80	331.19	G31.09
598	FTD	Other frontotemporal dementia (CODE)	331.19	G31.09	331.19	G31.09
599	FTD	Other frontotemporal dementia with behavioral disturbance	331.19, 294.11	G31.09, F02.818	331.19	F02.818
600	FTD	Other frontotemporal dementia with behavioral disturbance	331.19, 294.11	G31.09, F02.818	294.11	F02.818
601	FTD	Other frontotemporal dementia with behavioral disturbance	331.19, 294.11	G31.09, F02.818	331.19	G31.09
602	FTD	Other frontotemporal dementia with behavioral disturbance	331.19, 294.11	G31.09, F02.818	294.11	G31.09
603	FTD	Other frontotemporal dementia without behavioral disturbance	331.19, 294.10	G31.09, F02.80	331.19	F02.80
604	FTD	Other frontotemporal dementia without behavioral disturbance	331.19, 294.10	G31.09, F02.80	294.1	F02.80
605	FTD	Other frontotemporal dementia without behavioral disturbance	331.19, 294.10	G31.09, F02.80	331.19	G31.09
606	FTD	Other frontotemporal dementia without behavioral disturbance	331.19, 294.10	G31.09, F02.80	294.1	G31.09
607	FTD	Pick's disease	331.11	G31.01, F02.80	331.11	F02.80
608	FTD	Pick's disease	331.11	G31.01, F02.80	331.11	G31.01
609	FTD	Pick's disease	NULL	G31.01	NULL	G31.01
610	FTD	Pick's disease	331.11	G31.01, F02.80	331.11	F02.80
611	FTD	Pick's disease	331.11	G31.01, F02.80	331.11	G31.01
612	FTD	Possible major frontotemporal neurocognitive disorder	IMO0001	IMO0001	IMO0001	IMO0001
613	FTD	Probable major frontotemporal neurocognitive disorder with behavioral disturbance	IMO0001	IMO0001	IMO0001	IMO0001
614	FTD	Right temporal atrophy variant of frontotemporal dementia	331.19, 294.10	G31.09, F02.80	331.19	F02.80
615	FTD	Right temporal atrophy variant of frontotemporal dementia	331.19, 294.10	G31.09, F02.80	294.1	F02.80
616	FTD	Right temporal atrophy variant of frontotemporal dementia	331.19, 294.10	G31.09, F02.80	331.19	G31.09
617	FTD	Right temporal atrophy variant of frontotemporal dementia	331.19, 294.10	G31.09, F02.80	294.1	G31.09
618	LBD	Alzheimer's disease with Parkinson's disease	331.0, 332.0	G30.9, G20, F02.80	331	F02.80
619	LBD	Alzheimer's disease with Parkinson's disease	331.0, 332.0	G30.9, G20, F02.80	332	F02.80
620	LBD	Alzheimer's disease with Parkinson's disease	331.0, 332.0	G30.9, G20, F02.80	331	G20
621	LBD	Alzheimer's disease with Parkinson's disease	331.0, 332.0	G30.9, G20, F02.80	332	G20
622	LBD	Alzheimer's disease with Parkinson's disease	331.0, 332.0	G30.9, G20, F02.80	331	G30.9
623	LBD	Alzheimer's disease with Parkinson's disease	331.0, 332.0	G30.9, G20, F02.80	332	G30.9
624	LBD	Autosomal dominant Lewy body dementia	331.82	G31.83, F02.80	331.82	F02.80
625	LBD	Autosomal dominant Lewy body dementia	331.82	G31.83, F02.80	331.82	G31.83
626	LBD	Dementia due to Parkinson's disease with behavioral disturbance	332.0, 294.11	G20, F02.818	332	F02.818
627	LBD	Dementia due to Parkinson's disease with behavioral disturbance	332.0, 294.11	G20, F02.818	294.11	F02.818
628	LBD	Dementia due to Parkinson's disease with behavioral disturbance	332.0, 294.11	G20, F02.818	332	G20
629	LBD	Dementia due to Parkinson's disease with behavioral disturbance	332.0, 294.11	G20, F02.818	294.11	G20
630	LBD	Dementia due to Parkinson's disease without behavioral disturbance	332.0, 294.10	G20, F02.80	332	F02.80

No.	Category	DiagnosisNM	CurrentICD9ListTXT	CurrentICD10ListTXT	ICD9CD	ICD10CD
631	LBD	Dementia due to Parkinson's disease without behavioral disturbance	332.0, 294.10	G20, F02.80	294.1	F02.80
632	LBD	Dementia due to Parkinson's disease without behavioral disturbance	332.0, 294.10	G20, F02.80	332	G20
633	LBD	Dementia due to Parkinson's disease without behavioral disturbance	332.0, 294.10	G20, F02.80	294.1	G20
634	LBD	Dementia in Parkinson's plus syndrome	331.82, 294.10	G20, F02.80	331.82	F02.80
635	LBD	Dementia in Parkinson's plus syndrome	331.82, 294.10	G20, F02.80	294.1	F02.80
636	LBD	Dementia in Parkinson's plus syndrome	331.82, 294.10	G20, F02.80	331.82	G20
637	LBD	Dementia in Parkinson's plus syndrome	331.82, 294.10	G20, F02.80	294.1	G20
638	LBD	Dementia with Lewy bodies	331.82	G31.83, F02.80	331.82	F02.80
639	LBD	Dementia with Lewy bodies	331.82	G31.83, F02.80	331.82	G31.83
640	LBD	Dementia with Lewy bodies	331.82	G31.83, F02.80	331.82	F02.80
641	LBD	Dementia with Lewy bodies	331.82	G31.83, F02.80	331.82	G31.83
642	LBD	Dementia with Lewy bodies (CODE)	331.82	G31.83	331.82	G31.83
643	LBD	Dementia with parkinsonism	331.82, 294.10	G20, F02.80	331.82	F02.80
644	LBD	Dementia with parkinsonism	331.82, 294.10	G20, F02.80	294.1	F02.80
645	LBD	Dementia with parkinsonism	331.82, 294.10	G20, F02.80	331.82	G20
646	LBD	Dementia with parkinsonism	331.82, 294.10	G20, F02.80	294.1	G20
647	LBD	Dementia with parkinsonism	331.82, 294.10	G20.C, F02.80	294.1	G20.C
648	LBD	Dementia with parkinsonism	331.82, 294.10	G20.C, F02.80	331.82	G20.C
649	LBD	Dementia with parkinsonism	331.82, 294.10	G20.C, F02.80	294.1	F02.80
650	LBD	Dementia with parkinsonism	331.82, 294.10	G20.C, F02.80	331.82	F02.80
651	LBD	Dementia, Lewy body with behavior disturbance	331.82, 294.11	G31.83, F02.818	331.82	F02.818
652	LBD	Dementia, Lewy body with behavior disturbance	331.82, 294.11	G31.83, F02.818	294.11	F02.818
653	LBD	Dementia, Lewy body with behavior disturbance	331.82, 294.11	G31.83, F02.818	331.82	G31.83
654	LBD	Dementia, Lewy body with behavior disturbance	331.82, 294.11	G31.83, F02.818	294.11	G31.83
655	LBD	Lewy body dementia	331.82	G31.83, F02.80	331.82	F02.80
656	LBD	Lewy body dementia	331.82	G31.83, F02.80	331.82	G31.83
657	LBD	Lewy body dementia with behavioral disturbance	331.82, 294.11	G31.83, F02.818	331.82	F02.818
658	LBD	Lewy body dementia with behavioral disturbance	331.82, 294.11	G31.83, F02.818	294.11	F02.818
659	LBD	Lewy body dementia with behavioral disturbance	331.82, 294.11	G31.83, F02.818	331.82	G31.83
660	LBD	Lewy body dementia with behavioral disturbance	331.82, 294.11	G31.83, F02.818	294.11	G31.83
661	LBD	Lewy body dementia with other behavioral disturbance, unspecified dementia severity	331.82, 294.11	G31.83, F02.818	294.11	F02.818
662	LBD	Lewy body dementia with other behavioral disturbance, unspecified dementia severity	331.82, 294.11	G31.83, F02.818	294.11	G31.83
663	LBD	Lewy body dementia with other behavioral disturbance, unspecified dementia severity	331.82, 294.11	G31.83, F02.818	331.82	F02.818
664	LBD	Lewy body dementia with other behavioral disturbance, unspecified dementia severity	331.82, 294.11	G31.83, F02.818	331.82	G31.83
665	LBD	Lewy body dementia without behavioral disturbance	331.82, 294.10	G31.83, F02.80	331.82	F02.80
666	LBD	Lewy body dementia without behavioral disturbance	331.82, 294.10	G31.83, F02.80	294.1	F02.80
667	LBD	Lewy body dementia without behavioral disturbance	331.82, 294.10	G31.83, F02.80	331.82	G31.83
668	LBD	Lewy body dementia without behavioral disturbance	331.82, 294.10	G31.83, F02.80	294.1	G31.83
669	LBD	Lewy body dementia, unspecified dementia severity, unspecified whether behavioral, psychotic, or mood disturbance or anxiety	331.82	G31.83, F02.80	331.82	F02.80
670	LBD	Lewy body dementia, unspecified dementia severity, unspecified whether behavioral, psychotic, or mood disturbance or anxiety	331.82	G31.83, F02.80	331.82	G31.83
671	LBD	Lewy body disease	331.82	G31.83, F02.80	331.82	F02.80
672	LBD	Lewy body disease	331.82	G31.83, F02.80	331.82	G31.83

No.	Category	DiagnosisNM	CurrentICD9ListTXT	CurrentICD10ListTXT	ICD9CD	ICD10CD
673	LBD	Lewy body Parkinson disease	332.0, 331.82	G31.83, F02.80	331.82	F02.80
674	LBD	Lewy body Parkinson disease	332.0, 331.82	G31.83, F02.80	332	G31.83
675	LBD	Lewy body Parkinson disease	332.0, 331.82	G31.83, F02.80	331.82	G31.83
676	LBD	Lewy body Parkinson disease	332.0, 331.82	G31.83, F02.80	332	F02.80
677	LBD	Lewy body Parkinson's disease	332.0, 331.82	G31.83, F02.80	331.82	F02.80
678	LBD	Lewy body Parkinson's disease	332.0, 331.82	G31.83, F02.80	331.82	G31.83
679	LBD	Lewy body Parkinson's disease	332.0, 331.82	G31.83, F02.80	332	F02.80
680	LBD	Lewy body Parkinson's disease	332.0, 331.82	G31.83, F02.80	332	G31.83
681	LBD	Major neurocognitive disorder with Lewy bodies, probable, without behavioral disturbance	294.2	F03.90	294.2	F03.90
682	LBD	Mild major neurocognitive disorder with probable Lewy bodies, with behavioral disturbance	294.2	F03.91	294.2	F03.91
683	LBD	Mild major neurocognitive disorder with probable Lewy bodies, without behavioral disturbance	294.2	F03.90	294.2	F03.90
684	LBD	Mild neurocognitive disorder with Lewy bodies	331.83	G31.83, F02.A0	331.83	F02.A0
685	LBD	Mild neurocognitive disorder with Lewy bodies	331.83	G31.83, F02.A0	331.83	G31.83
686	LBD	Mild possible major neurocognitive disorder with Lewy bodies	IMO0001	IMO0001	IMO0001	IMO0001
687	LBD	Mild probable major neurocognitive disorder with Lewy bodies without behavioral disturbance	294.2	F03.90	294.2	F03.90
688	LBD	Motor fluctuations related to medication use in Parkinson's disease	332.0, E936.4	G20.A2, T42.8X5A	332	T42.8X5A
689	LBD	Motor fluctuations related to medication use in Parkinson's disease	332.0, E936.4	G20.A2, T42.8X5A	E936.4	T42.8X5A
690	LBD	Motor fluctuations related to medication use in Parkinson's disease	332.0, E936.4	G20.A2, T42.8X5A	332	G20.A2
691	LBD	Motor fluctuations related to medication use in Parkinson's disease	332.0, E936.4	G20.A2, T42.8X5A	E936.4	G20.A2
692	LBD	Multiple system atrophy, Parkinson variant	333	G23.2	333	G23.2
693	LBD	Neurocognitive disorder with Lewy bodies (CODE)	331.82, 294.10	G31.83	331.82	G31.83
694	LBD	Neurocognitive disorder with Lewy bodies (CODE)	331.82, 294.10	G31.83	294.1	G31.83
695	LBD	Neuroleptic-induced parkinsonism	332.1, E939.3	G21.11, T43.505A	332.1	T43.505A
696	LBD	Neuroleptic-induced parkinsonism	332.1, E939.3	G21.11, T43.505A	332.1	G21.11
697	LBD	Neuroleptic-induced parkinsonism	332.1, E939.3	G21.11, T43.505A	E939.3	G21.11
698	LBD	Neuroleptic-induced parkinsonism	332.1, E939.3	G21.11, T43.505A	E939.3	T43.505A
699	LBD	Other secondary parkinsonism	332.1	G21.8	332.1	G21.8
700	LBD	Parkinson disease	332	G20.A1	332	G20.A1
701	LBD	Parkinson-dementia complex of Guam	332.1	G12.21, F02.80, G20	332.1	G20
702	LBD	Parkinson-dementia complex of Guam	332.1	G12.21, F02.80, G20	332.1	F02.80
703	LBD	Parkinson-dementia complex of Guam	332.1	G12.21, F02.80, G20	332.1	G12.21
704	LBD	Parkinson's disease	NULL	G20	NULL	G20
705	LBD	Parkinson's disease	332	NULL	332	NULL
706	LBD	Parkinson's disease	332	G20.A1	332	G20.A1
707	LBD	Parkinson's disease dementia	332.0, 294.10	G20, F02.80	332	F02.80
708	LBD	Parkinson's disease dementia	332.0, 294.10	G20, F02.80	294.1	F02.80
709	LBD	Parkinson's disease dementia	332.0, 294.10	G20, F02.80	332	G20
710	LBD	Parkinson's disease dementia	332.0, 294.10	G20, F02.80	294.1	G20
711	LBD	Parkinson's disease dementia	332.0, 294.10	G20.A1, F02.80	294.1	G20.A1
712	LBD	Parkinson's disease dementia	332.0, 294.10	G20.A1, F02.80	294.1	F02.80
713	LBD	Parkinson's disease dementia	332.0, 294.10	G20.A1, F02.80	332	F02.80
714	LBD	Parkinson's disease dementia	332.0, 294.10	G20.A1, F02.80	332	G20.A1

No.	Category	DiagnosisNM	CurrentICD9ListTXT	CurrentICD10ListTXT	ICD9CD	ICD10CD
715	LBD	Parkinson's disease without dyskinesia or fluctuating manifestations	332	G20.A1	332	G20.A1
716	LBD	Parkinson's disease without dyskinesia, with fluctuating manifestations	332	G20.A2	332	G20.A2
717	LBD	Parkinson's disease, Lewy body	332.0, 331.82	G31.83, F02.80	331.82	F02.80
718	LBD	Parkinson's disease, Lewy body	332.0, 331.82	G31.83, F02.80	332	G31.83
719	LBD	Parkinson's disease, Lewy body	332.0, 331.82	G31.83, F02.80	331.82	G31.83
720	LBD	Parkinson's disease, Lewy body	332.0, 331.82	G31.83, F02.80	332	F02.80
721	LBD	Parkinson's disease, unspecified whether dyskinesia present, unspecified whether manifestations fluctuate	332	G20.A1	332	G20.A1
722	LBD	Parkinson's plus syndrome	332	G20.C	332	G20.C
723	LBD	Parkinson's syndrome	332	G20.A1	332	G20.A1
724	LBD	Parkinsonian features	781	R29.818	781	R29.818
725	LBD	Parkinsonian tremor	332	G20.C	332	G20.C
726	LBD	Parkinsonism	332	G20.C	332	G20.C
727	LBD	Parkinsonism due to drug	332.1, E980.5	G21.19	332.1	G21.19
728	LBD	Parkinsonism due to drug	332.1, E980.5	G21.19	E980.5	G21.19
729	LBD	Parkinsonism of lower half of body	332	G20.C	332	G20.C
730	LBD	Parkinsonism with orthostatic hypotension	333	G20.C, I95.1	333	I95.1
731	LBD	Parkinsonism with orthostatic hypotension	333	G20.C, I95.1	333	G20.C
732	LBD	Parkinsonism, unspecified Parkinsonism type	332	G20.C	332	G20.C
733	LBD	Parkinsons	332	G20.A1	332	G20.A1
734	LBD	Parkinsons disease	332	G20.A1	332	G20.A1
735	LBD	Primary parkinsonism	332	G20.C	332	G20.C
736	LBD	Secondary parkinsonism due to other external agents	332.1	G21.2	332.1	G21.2
737	LBD	Secondary parkinsonism, unspecified secondary Parkinsonism type	332.1	G21.9	332.1	G21.9
738	LBD	Vascular parkinsonism	332	G21.4	332	G21.4
739	MCI	Amnestic MCI (mild cognitive impairment with memory loss)	331.83	G31.84	331.83	G31.84
740	MCI	Cognitive impairment, mild, so stated	331.83	G31.84	331.83	G31.84
741	MCI	MCI (mild cognitive impairment)	331.83	G31.84	331.83	G31.84
742	MCI	MCI (mild cognitive impairment) with memory loss	331.83	G31.84	331.83	G31.84
743	MCI	Mild cognitive disorder	294.9	F09	294.9	F09
744	MCI	Mild cognitive disorder	294.9	F09	294.9	F09
745	MCI	Mild cognitive impairment	331.83	G31.84	331.83	G31.84
746	MCI	Mild cognitive impairment with memory loss	331.83	G31.84	331.83	G31.84
747	MCI	Mild cognitive impairment, so stated	331.83	G31.84	331.83	G31.84
748	MCI	Mild neurocognitive disorder	331.83	G31.84	331.83	G31.84
749	MCI	Minimal cognitive impairment	331.83	G31.84	331.83	G31.84
750	MCI	Minor neurocognitive disorder	294.9	G31.84	294.9	G31.84
751	Other	Alzheimer's disease with early onset	331	G30.0, F02.80	331	F02.80
752	Other	Alzheimer's disease with early onset	331	G30.0, F02.80	331	G30.0
753	Other	Alzheimer's disease with early onset (CODE)	331	G30.0	331	G30.0
754	Other	Atypical progressive supranuclear palsy	333	G23.1	333	G23.1
755	Other	Cerebral amyloid angiopathy	NULL	I68.0	NULL	I68.0
756	Other	Cerebral amyloid angiopathy	277.39, 437.9	E85.4, I68.0	277.39	I68.0

No.	Category	DiagnosisNM	CurrentICD9ListTXT	CurrentICD10ListTXT	ICD9CD	ICD10CD
757	Other	Cerebral amyloid angiopathy	277.39, 437.9	E85.4, I68.0	437.9	I68.0
758	Other	Cerebral amyloid angiopathy	277.39, 437.9	E85.4, I68.0	277.39	E85.4
759	Other	Cerebral amyloid angiopathy	277.39, 437.9	E85.4, I68.0	437.9	E85.4
760	Other	Cognitive and neurobehavioral dysfunction	294.9	F09	294.9	F09
761	Other	Complaint of memory disorder without observed objective memory deficit	780.93	R41.3	780.93	R41.3
762	Other	Corticobasal degeneration	331.6	G31.85	331.6	G31.85
763	Other	Corticobasal syndrome	334.2	G31.85	334.2	G31.85
764	Other	Dementia in Alzheimer's disease with early onset	331.0, 294.10	G30.0, F02.80	331	G30.0
765	Other	Dementia in Alzheimer's disease with early onset	331.0, 294.10	G30.0, F02.80	294.1	G30.0
766	Other	Dementia in Alzheimer's disease with early onset	331.0, 294.10	G30.0, F02.80	331	F02.80
767	Other	Dementia in Alzheimer's disease with early onset	331.0, 294.10	G30.0, F02.80	294.1	F02.80
768	Other	Dementia in Alzheimer's disease with early onset with behavioral disturbance	331.0, 294.11	G30.0, F02.818	294.11	F02.818
769	Other	Dementia in Alzheimer's disease with early onset with behavioral disturbance	331.0, 294.11	G30.0, F02.818	331	F02.818
770	Other	Dementia in Alzheimer's disease with early onset with behavioral disturbance	331.0, 294.11	G30.0, F02.818	331	G30.0
771	Other	Dementia in Alzheimer's disease with early onset with behavioral disturbance	331.0, 294.11	G30.0, F02.818	294.11	G30.0
772	Other	Dementia in Alzheimer's disease with early onset without behavioral disturbance	331.0, 294.10	G30.0, F02.80	294.1	G30.0
773	Other	Dementia in Alzheimer's disease with early onset without behavioral disturbance	331.0, 294.10	G30.0, F02.80	294.1	F02.80
774	Other	Dementia in Alzheimer's disease with early onset without behavioral disturbance	331.0, 294.10	G30.0, F02.80	331	F02.80
775	Other	Dementia in Alzheimer's disease with early onset without behavioral disturbance	331.0, 294.10	G30.0, F02.80	331	G30.0
776	Other	Dementia in Alzheimer's disease with early onset, without behavioral disturbance	331.0, 294.10	F02.80	331	F02.80
777	Other	Dementia in Alzheimer's disease with early onset, without behavioral disturbance	331.0, 294.10	F02.80	294.1	F02.80
778	Other	Dementia in corticobasal degeneration	331.6, 294.10	G31.85, F02.80	294.1	F02.80
779	Other	Dementia in corticobasal degeneration	331.6, 294.10	G31.85, F02.80	294.1	G31.85
780	Other	Dementia in corticobasal degeneration	331.6, 294.10	G31.85, F02.80	331.6	F02.80
781	Other	Dementia in corticobasal degeneration	331.6, 294.10	G31.85, F02.80	331.6	G31.85
782	Other	Dementia in progressive supranuclear ophthalmoplegia	333.0, 294.10	G23.1, F02.80	294.1	F02.80
783	Other	Dementia in progressive supranuclear ophthalmoplegia	333.0, 294.10	G23.1, F02.80	333	F02.80
784	Other	Dementia in progressive supranuclear ophthalmoplegia	333.0, 294.10	G23.1, F02.80	333	G23.1
785	Other	Dementia in progressive supranuclear ophthalmoplegia	333.0, 294.10	G23.1, F02.80	294.1	G23.1
786	Other	Dementia with visual impairment due to posterior cerebral cortical atrophy	290.10, 369.9	G31.9, F02.80, H54.7	290.1	G31.9
787	Other	Dementia with visual impairment due to posterior cerebral cortical atrophy	290.10, 369.9	G31.9, F02.80, H54.7	369.9	G31.9
788	Other	Dementia with visual impairment due to posterior cerebral cortical atrophy	290.10, 369.9	G31.9, F02.80, H54.7	290.1	F02.80
789	Other	Dementia with visual impairment due to posterior cerebral cortical atrophy	290.10, 369.9	G31.9, F02.80, H54.7	290.1	H54.7
790	Other	Dementia with visual impairment due to posterior cerebral cortical atrophy	290.10, 369.9	G31.9, F02.80, H54.7	369.9	F02.80
791	Other	Dementia with visual impairment due to posterior cerebral cortical atrophy	290.10, 369.9	G31.9, F02.80, H54.7	369.9	H54.7
792	Other	Early onset Alzheimer's dementia without behavioral disturbance	331.0, 294.10	G30.0, F02.80	331	G30.0
793	Other	Early onset Alzheimer's dementia without behavioral disturbance	331.0, 294.10	G30.0, F02.80	294.1	G30.0
794	Other	Early onset Alzheimer's dementia without behavioral disturbance	331.0, 294.10	G30.0, F02.80	331	F02.80
795	Other	Early onset Alzheimer's dementia without behavioral disturbance	331.0, 294.10	G30.0, F02.80	294.1	F02.80
796	Other	Early onset Alzheimer's dementia without behavioral disturbance, psychotic disturbance, mood disturbance, or anxiety, unspecified dementia severity	331.0, 294.10	G30.0, F02.80	331	G30.0
797	Other	Early onset Alzheimer's dementia without behavioral disturbance, psychotic disturbance, mood disturbance, or anxiety, unspecified dementia severity	331.0, 294.10	G30.0, F02.80	294.1	G30.0
798	Other	Early onset Alzheimer's dementia without behavioral disturbance, psychotic disturbance, mood disturbance, or anxiety, unspecified dementia severity	331.0, 294.10	G30.0, F02.80	331	F02.80

No.	Category	DiagnosisNM	CurrentICD9ListTXT	CurrentICD10ListTXT	ICD9CD	ICD10CD
799	Other	Early onset Alzheimer's dementia without behavioral disturbance, psychotic disturbance, mood disturbance, or anxiety, unspecified dementia severity	331.0, 294.10	G30.0, F02.80	294.1	F02.80
800	Other	Early onset Alzheimer's disease with behavioral disturbance	331.0, 294.11	G30.0, F02.818	294.11	F02.818
801	Other	Early onset Alzheimer's disease with behavioral disturbance	331.0, 294.11	G30.0, F02.818	331	F02.818
802	Other	Early onset Alzheimer's disease with behavioral disturbance	331.0, 294.11	G30.0, F02.818	294.11	G30.0
803	Other	Early onset Alzheimer's disease with behavioral disturbance	331.0, 294.11	G30.0, F02.818	331	G30.0
804	Other	History of progressive supranuclear palsy	V12.49	Z86.69	V12.49	Z86.69
805	Other	Idiopathic normal pressure hydrocephalus	331.5	G91.2	331.5	G91.2
806	Other	Mild early onset Alzheimer's dementia with anxiety	331.0, 294.11	G30.0, F02.A4	294.11	G30.0
807	Other	Mild early onset Alzheimer's dementia with anxiety	331.0, 294.11	G30.0, F02.A4	331	F02.A4
808	Other	Mild early onset Alzheimer's dementia with anxiety	331.0, 294.11	G30.0, F02.A4	294.11	F02.A4
809	Other	Mild early onset Alzheimer's dementia with anxiety	331.0, 294.11	G30.0, F02.A4	331	G30.0
810	Other	Normal pressure hydrocephalus	331.5	G91.2	331.5	G91.2
811	Other	Normal pressure hydrocephalus syndrome	331.5	G91.2	331.5	G91.2
812	Other	NPH (normal pressure hydrocephalus)	331.5	G91.2	331.5	G91.2
813	Other	Posterior Cortical Atrophy	331.9	G31.9	331.9	G31.9
814	Other	Posterior cortical atrophy	331.9	G31.9	331.9	G31.9
815	Other	Primary progressive aphasia	784.3	G31.01, F02.80	784.3	F02.80
816	Other	Primary progressive aphasia	784.3	G31.01, F02.80	784.3	G31.01
817	Other	Progressive aphasia in Alzheimer's disease	331.0, 784.3	G30.9, R47.01	331	R47.01
818	Other	Progressive aphasia in Alzheimer's disease	331.0, 784.3	G30.9, R47.01	784.3	R47.01
819	Other	Progressive aphasia in Alzheimer's disease	331.0, 784.3	G30.9, R47.01	331	G30.9
820	Other	Progressive aphasia in Alzheimer's disease	331.0, 784.3	G30.9, R47.01	784.3	G30.9
821	Other	Progressive supranuclear palsy	333	G23.1	333	G23.1
822	Other	Pseudodementia	799.59	R41.89	799.59	R41.89
823	Other	PSP (progressive supranuclear palsy)	333	G23.1	333	G23.1
824	Other	Secondary normal pressure hydrocephalus	331.3	G91.0	331.3	G91.0
825	Other	Subjective cognitive impairment	799.59	R41.89	799.59	R41.89
826	Other	Subjective memory complaints	780.93	R41.89	780.93	R41.89
827	VCI	Alterations of sensations, late effect of cerebrovascular disease(438.6)	438.6	I69.998, R20.9	438.6	R20.9
828	VCI	Alterations of sensations, late effect of cerebrovascular disease(438.6)	438.6	I69.998, R20.9	438.6	I69.998
829	VCI	Aphasia as late effect of cerebrovascular accident	438.11	I69.320	438.11	I69.320
830	VCI	Cerebral microvascular disease	437.8	I67.89	437.8	I67.89
831	VCI	Cerebral vascular disease	437.9	I67.9	437.9	I67.9
832	VCI	Cerebrovascular disease	437.9	I67.9	437.9	I67.9
833	VCI	Cerebrovascular disease, unspecified	NULL	I67.9	NULL	I67.9
834	VCI	Cerebrovascular disease, unspecified	437.9	I67.9	437.9	I67.9
835	VCI	Cerebrovascular small vessel disease	437.9	I67.9	437.9	I67.9
836	VCI	Cognitive deficits as late effect of cerebrovascular disease	438	I69.919	438	I69.919
837	VCI	Cognitive deficits following cerebrovascular disease	438	I69.919	438	I69.919
838	VCI	Cognitive deficits, late effect of cerebrovascular disease	438	I69.919	438	I69.919
839	VCI	Dementia, vascular	290.4	F01.50	290.4	F01.50
840	VCI	Dementia, vascular, mixed	290.4	F01.50	290.4	F01.50

No.	Category	DiagnosisNM	CurrentICD9ListTXT	CurrentICD10ListTXT	ICD9CD	ICD10CD
841	VCI	Dementia, vascular, mixed, with behavioral disturbance	290.41	F01.518	290.41	F01.518
842	VCI	Dementia, vascular, mixed, without behavioral disturbance	290.4	F01.50	290.4	F01.50
843	VCI	DM (diabetes mellitus) type II controlled peripheral vascular disorder	250.70, 443.81	E11.51	250.7	E11.51
844	VCI	DM (diabetes mellitus) type II controlled peripheral vascular disorder	250.70, 443.81	E11.51	443.81	E11.51
845	VCI	Major neurocognitive disorder due to vascular disease, without behavioral disturbance, severe	459.9, 294.9	F01.C0	459.9	F01.C0
846	VCI	Major neurocognitive disorder due to vascular disease, without behavioral disturbance, severe	459.9, 294.9	F01.C0	294.9	F01.C0
847	VCI	Major neurocognitive disorder probably due to vascular disease, without behavioral disturbance	294.2	F03.90	294.2	F03.90
848	VCI	Memory deficit after cerebrovascular disease	438.0, 780.93	I69.911	438	I69.911
849	VCI	Memory deficit after cerebrovascular disease	438.0, 780.93	I69.911	780.93	I69.911
850	VCI	Mild vascular dementia with anxiety	290.4	F01.A4	290.4	F01.A4
851	VCI	Mild vascular neurocognitive disorder	459.9, 294.9	I99.9, F06.70	459.9	F06.70
852	VCI	Mild vascular neurocognitive disorder	459.9, 294.9	I99.9, F06.70	294.9	F06.70
853	VCI	Mild vascular neurocognitive disorder	459.9, 294.9	I99.9, F06.70	459.9	I99.9
854	VCI	Mild vascular neurocognitive disorder	459.9, 294.9	I99.9, F06.70	294.9	I99.9
855	VCI	Mixed cortical and subcortical vascular dementia with behavioral disturbance	290.41	F01.518	290.41	F01.518
856	VCI	Mixed cortical and subcortical vascular dementia without behavioral disturbance	290.4	F01.50	290.4	F01.50
857	VCI	Mixed cortical and subcortical vascular dementia, without behavioral disturbance	290.4	F01.50	290.4	F01.50
858	VCI	Mixed vascular and neurodegenerative dementia	290.40, 290.8	F01.50	290.4	F01.50
859	VCI	Mixed vascular and neurodegenerative dementia	290.40, 290.8	F01.50	290.8	F01.50
860	VCI	Mixed vascular and neurodegenerative dementia with behavioral disturbance	290.40, 294.11	F01.518	290.4	F01.518
861	VCI	Mixed vascular and neurodegenerative dementia with behavioral disturbance	290.40, 294.11	F01.518	294.11	F01.518
862	VCI	Mixed vascular and neurodegenerative dementia without behavioral disturbance	290.40, 294.10	F01.50	290.4	F01.50
863	VCI	Mixed vascular and neurodegenerative dementia without behavioral disturbance	290.40, 294.10	F01.50	294.1	F01.50
864	VCI	Mixed vascular and neurodegenerative dementia, with behavioral disturbance	290.40, 294.11	F01.518	290.4	F01.518
865	VCI	Mixed vascular and neurodegenerative dementia, with behavioral disturbance	290.40, 294.11	F01.518	294.11	F01.518
866	VCI	Mixed vascular and neurodegenerative dementia, without behavioral disturbance	290.40, 294.10	F01.50	290.4	F01.50
867	VCI	Mixed vascular and neurodegenerative dementia, without behavioral disturbance	290.40, 294.10	F01.50	294.1	F01.50
868	VCI	Moderate mild vascular neurocognitive disorder	331.83	F01.B0	331.83	F01.B0
869	VCI	Moderate vascular dementia without behavioral disturbance, psychotic disturbance, mood disturbance, or anxiety	290.4	F01.B0	290.4	F01.B0
870	VCI	Mood disorder due to cerebrovascular accident	IMO0002	IMO0002	IMO0002	IMO0002
871	VCI	Multi-infarct dementia, without behavioral disturbance	290.4	F01.50	290.4	F01.50
872	VCI	Other late effects of cerebrovascular disease(438.89)	438.89	I69.998	438.89	I69.998
873	VCI	Other vascular headache	784	G44.1	784	G44.1
874	VCI	Probable major vascular neurocognitive disorder	IMO0001	IMO0001	IMO0001	IMO0001
875	VCI	Small vessel disease, cerebrovascular	437.9	I67.9	437.9	I67.9
876	VCI	Speech and language deficits, late effect of cerebrovascular disease	438.1	I69.928	438.1	I69.928
877	VCI	Speech and language deficits, late effect of cerebrovascular disease	438.1	NULL	438.1	NULL
878	VCI	Subcortical vascular dementia	290.4	F01.50	290.4	F01.50
879	VCI	Subcortical vascular dementia without behavioral disturbance	290.4	F01.50	290.4	F01.50
880	VCI	Subcortical vascular dementia, with behavioral disturbance	290.41	F01.518	290.41	F01.518
881	VCI	VAD (vascular dementia)	290.4	F01.50	290.4	F01.50
882	VCI	VAD (vascular dementia), with behavioral disturbance	290.4	F01.51	290.4	F01.51

No.	Category	DiagnosisNM	CurrentICD9ListTXT	CurrentICD10ListTXT	ICD9CD	ICD10CD
883	VCI	Vascular abnormality of brain	747.81	Q28.3	747.81	Q28.3
884	VCI	Vascular dementia of acute onset with behavioral disturbance	290.41	F01.518	290.41	F01.518
885	VCI	Vascular dementia of acute onset without behavioral disturbance	290.4	F01.50	290.4	F01.50
886	VCI	Vascular dementia with behavior disturbance	290.4	F01.518	290.4	F01.518
887	VCI	Vascular dementia with behavioral disturbance	290.4	F01.518	290.4	F01.518
888	VCI	Vascular dementia with delirium	290.41	F01.50, F05	290.41	F05
889	VCI	Vascular dementia with delirium	290.41	F01.50, F05	290.41	F01.50
890	VCI	Vascular dementia with delirium	290.41	F01.50, F05	290.41	F05
891	VCI	Vascular dementia with delirium	290.41	F01.50, F05	290.41	F01.50
892	VCI	Vascular dementia with delusions	290.42	F01.52	290.42	F01.52
893	VCI	Vascular dementia with depressed mood	290.43	F01.53	290.43	F01.53
894	VCI	Vascular dementia with paranoia	290.42	F01.52	290.42	F01.52
895	VCI	Vascular dementia without behavioral disturbance	290.4	F01.50	290.4	F01.50
896	VCI	Vascular dementia without behavioral disturbance, psychotic disturbance, mood disturbance, or anxiety, unspecified dementia severity	290.4	F01.50	290.4	F01.50
897	VCI	Vascular dementia, uncomplicated	290.4	F01.50	290.4	F01.50
898	VCI	Vascular dementia, with behavioral disturbance	290.4	F01.51	290.4	F01.51
899	VCI	Vascular dementia, without behavioral disturbance	290.4	F01.50	290.4	F01.50
900	VCI	Visual cortex disorder due to vascular disorder, right	377.72	H47.641	377.72	H47.641
