# Supplementary material for: Unsupervised Deep Learning of Electronic Health Records to Characterize Heterogeneity Across Alzheimer Disease and Related Dementias: Cross-Sectional Study
Source: JMIR Aging. 2025 Mar 31;8:e65178. doi: 10.2196/65178 (PMC11997524; doi:10.2196/65178)
Supplement: Multimedia Appendix 2 [file aging_v8i1e65178_app2.docx]

**Supplement**


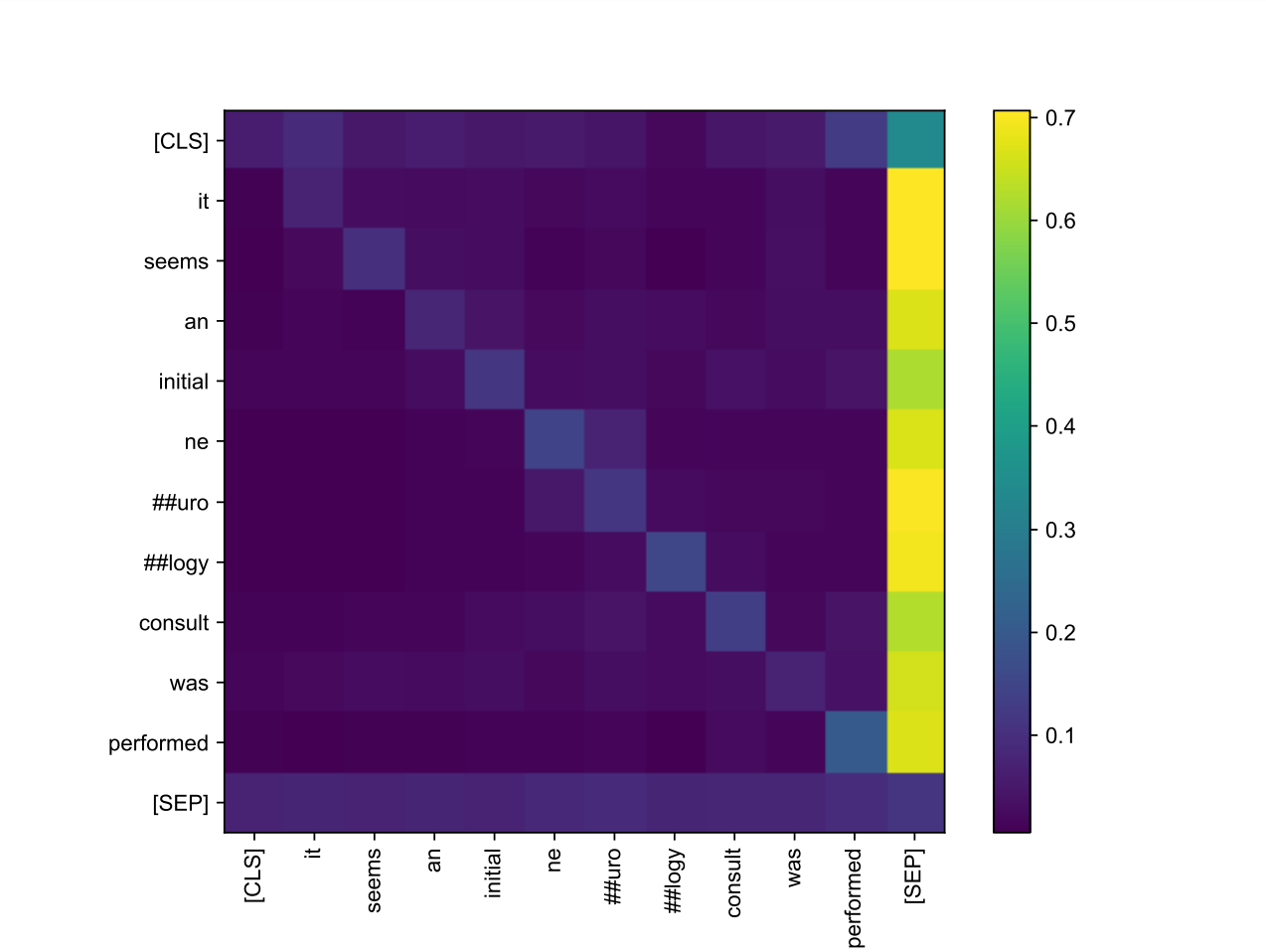


**Figure S1. Attention heatmap of final layer for example input sequence, averaged over all 12 attention heads.** This attention matrix exhibits high attention weights to the [SEP] token for most tokens, and slightly higher row-wise entropy (and therefore higher weight in averaging) for ‘initial’ and ‘consult’.


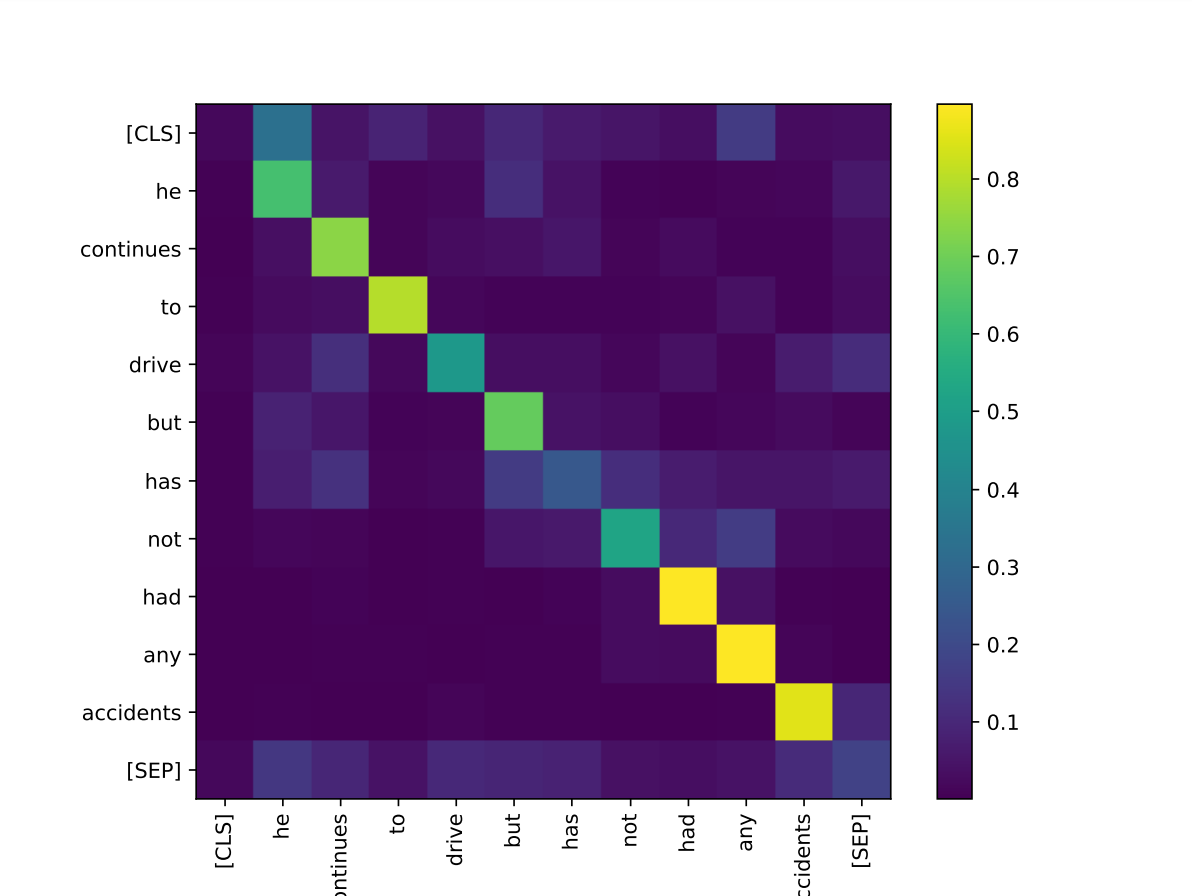


**Figure S2. Attention heatmap of final layer for example input sequence, for head 3 alone.**

Most tokens attend heavily to themselves, indicating relatively low row-wise entropy. Examples of tokens with higher row-wise entropy that attend to multiple tokens include ‘drive’, ‘has’, and ‘not’ - embeddings from these tokens would receive a higher weight when performing attention-weighted averaging.


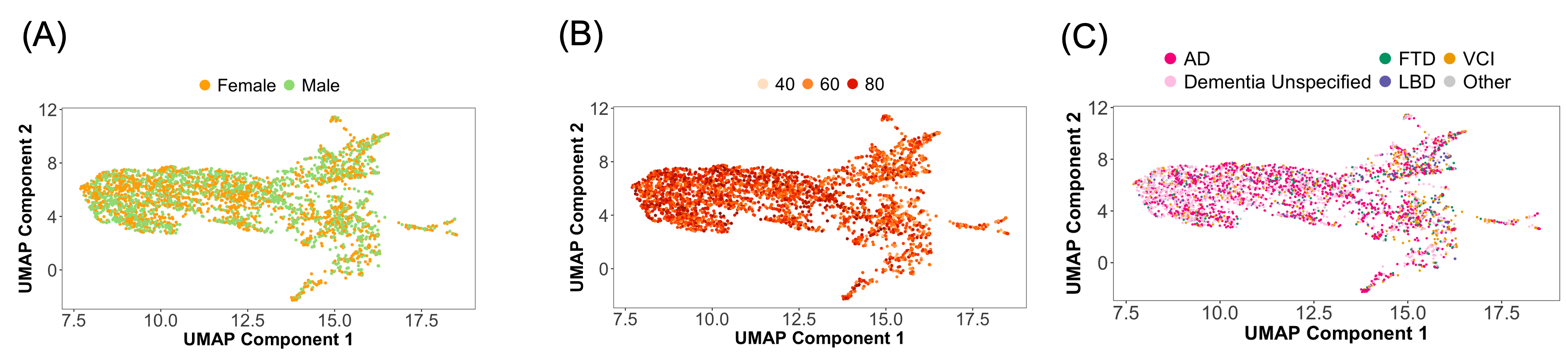


**Figure S3. UMAP visualization of ICD embeddings characterized by (A) Sex, (B) Age of onset, and (C) ADRD diagnosis.**


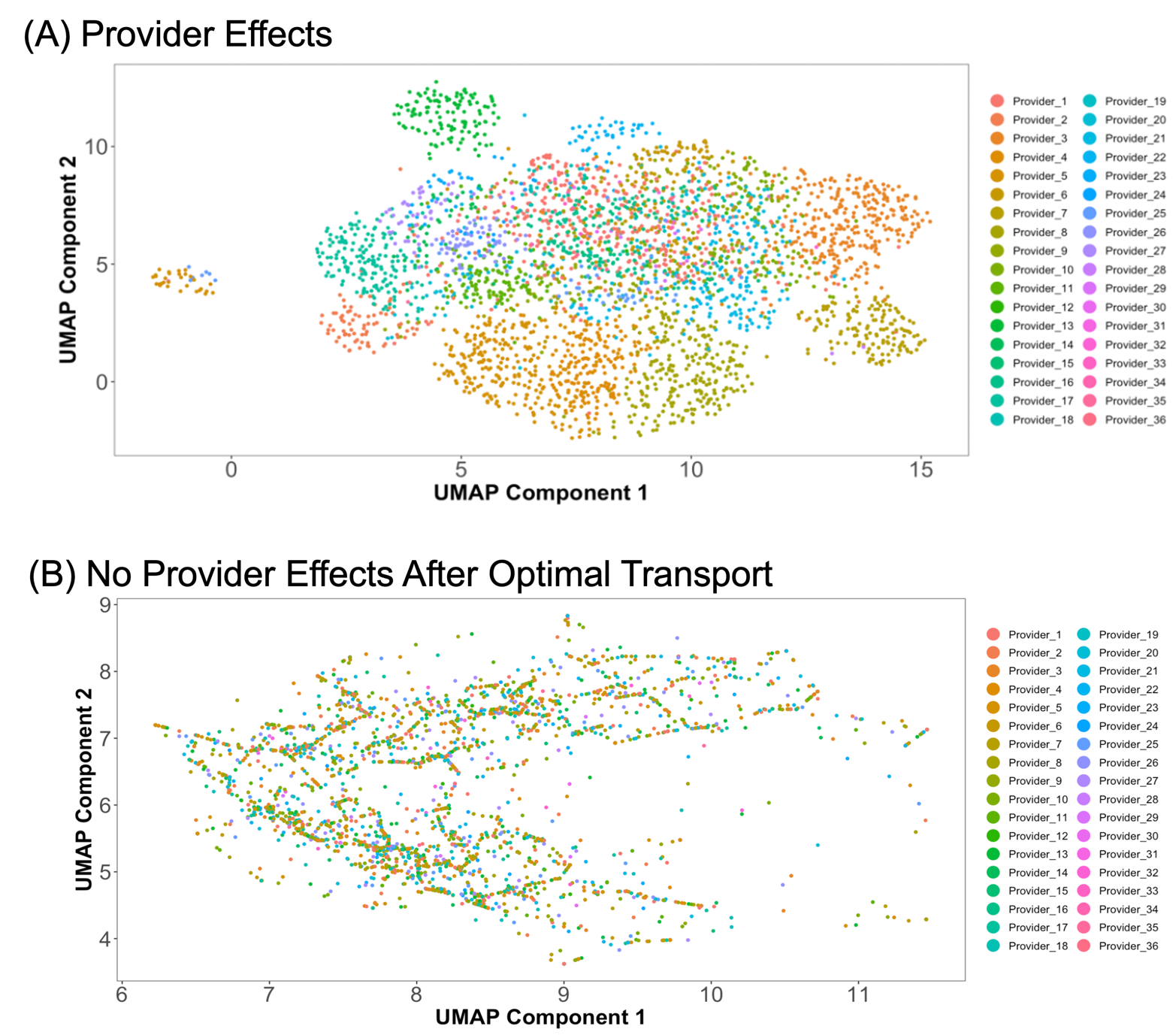


**Figure S4: UMAP Projections of Note Embeddings Characterized by Providers.**

(A) The embeddings before applying optimal transport, highlighting the visible provider effects. (B) The embeddings after optimal transport, demonstrating the mitigation of provider effects. Provider names have been de-identified to maintain confidentiality.


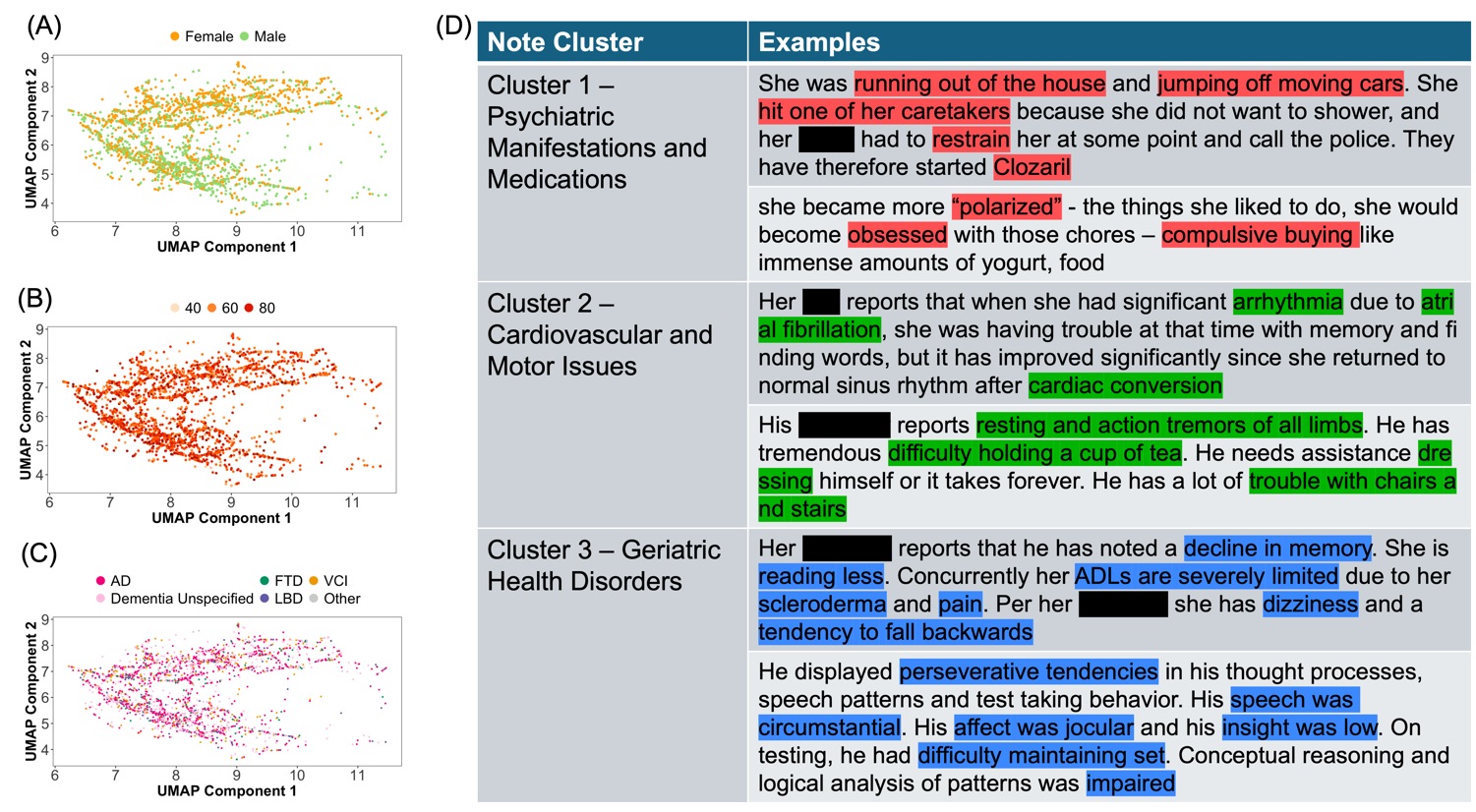


**Figure S5**. **UMAP visualization of note embeddings characterized by (A) Sex (B) Age of onset, (C) ADRD diagnoses, and (D) Sentence examples from each note cluster, with topic related terms and phrases highlighted.**
